# Similar Habitat Gradients Across Native and Invasive Ranges Elicit Divergent Evolutionary Responses

**DOI:** 10.1002/ece3.74407

**Published:** 2026-09-23

**Authors:** Kelton Cheung, Richard J. Edwards, Jayna L. DeVore, Simon Ducatez, Cameron M. Hudson, Richard Shine, Lee A. Rollins

**Affiliations:** ^1^ Evolution & Ecology Research Centre, School of Biological, Earth & Environmental Sciences University of New South Wales Sydney New South Wales Australia; ^2^ Evolution & Ecology Research Centre, School of Biotechnology & Biomolecular Sciences University of New South Wales Sydney New South Wales Australia; ^3^ UWA Oceans Institute The University of Western Australia Perth Western Australia Australia; ^4^ Univ. Polynésie Française UMR SECOPOL (UPF, IRD, IFREMER, ILM) BP 6570 Faa'a Tahiti French Polynesia; ^5^ Institut de Recherche Pour le Développement (IRD) UMR SECOPOL (UPF, IRD, IFREMER, ILM) BP 6570 Faa'a Tahiti French Polynesia; ^6^ Department of Aquatic Ecology, Eawag Swiss Federal Institute of Aquatic Science and Technology Zürich Switzerland; ^7^ School of Natural Sciences Macquarie University Sydney New South Wales Australia

**Keywords:** amphibian, cane toad, DArT‐seq, environmentally associated selection, morphology, *Rhinella marina*

## Abstract

Knowledge about an invasive species' biology within its native range illuminates the underlying evolutionary processes that occur following introduction. Cane toads ( *Rhinella marina* ) were introduced to coastal Queensland in 1935 and continue to expand their range across Australia. Despite low genetic diversity, they have accelerated their expansion across novel, arid environments as they travelled westward, differing from their eastern Australian conspecifics in morphology, physiology, and behaviour. The recent discovery of two ecotypes (from wetter ‘rainforest’ and drier ‘coastal’ areas) in the native range raises the question of how much of this geographical variation in Australia is due to (a) selection following introduction as toads encountered environmental variation, or (b) sorting of native range alleles into the environments similar to those in the native range. We used morphological data of 296 native range toads to demonstrate body‐size differences between ecotypes. Then we used genetic data of 93 native and invasive toads to investigate whether native‐range ecotypes are genetically distinct and whether patterns of selection for wetter versus drier environments in native and invasive populations were similar. We found moderate population structure within the native range across space and environments. When contrasting environmental gradients in the native versus invasive ranges, we identified distinct, non‐overlapping sets of outlier SNPs. Nearly 80% of outlier SNPs detected in the invasive range were polymorphic in the native range, suggesting that genetic patterns in invasive toads have been primarily driven by standing variation from the native range. This study provides insights into evolutionary patterns occurring during biological invasion, demonstrating distinct evolutionary responses to similar environments.

## Graphical abstract

We used morphological and genetic data of native and invasive cane toads to demonstrate 1) body‐size differences between ecotypes, 2) whether native‐range ecotypes are genetically distinct and 3) whether patterns of selection for wetter versus drier environments in native and invasive populations were similar. We found moderate population structure within the native range across space and environments. When contrasting environmental gradients in the native versus invasive ranges, we identified distinct, non‐overlapping sets of outlier SNPs. Nearly 80% of outlier SNPs detected in the invasive range were polymorphic in the native range, suggesting that genetic patterns in invasive toads have been primarily driven by standing variation from the native range.

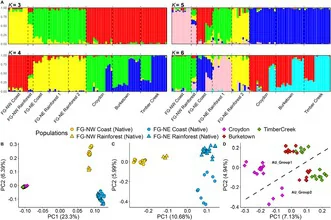

## 1 Introduction

During range expansion, novel environments may impose selective regimes that can lead to phenotypic (Schluter 2000) and genomic (Naciri and Linder 2020) divergence across populations. Alternatively, under comparable selective regimes, similar phenotypes may evolve independently, a phenomenon called repeated evolution (Cerca 2023). Studying these outcomes offers important insights into the predictability of evolution. The ability of invaders to rapidly adapt to novel environments provides a unique opportunity to understand mechanisms underlying selection. Post‐introduction adaptation may be facilitated by selection on *de novo* mutations that offer fitness advantages (Dlugosch et al. 2015), or by introgression and admixture following introductions from multiple genetically differentiated source populations that enhance genetic combinations for adaptation. Selection also may act on standing genetic variation that was present in the native range prior to introduction (Battlay et al. 2023; Dogantzis et al. 2024; Thia 2022).

However, studying the underlying genetic and ecological processes of repeated evolution in situ poses a major challenge because evolutionary changes are typically slow in pace. Also, similar traits may not always reflect independent adaptation but instead the reuse of standing genetic variation, either inherited from a common ancestor or exchanged via gene flow (Smadja and Butlin 2011). These complications mean that examples of repeated evolution in native, wild populations remain relatively scarce, including three‐spined stickleback (Jones et al. 2012; Magalhaes et al. 2021), Galápagos beetles (Vangestel et al. 2024) and cavefish (Herman et al. 2018). In contrast, biological invasions serve as invaluable, natural experiments that can be used to address these challenges. Independent introductions from often‐known source populations create contemporary natural experiments in which selection can be observed directly when species encounter novel environments.

The cane toad ( *Rhinella marina* , formerly *Bufo marinus* ) invasion in Australia exemplifies the potential of a model system for studying repeated evolution. Cane toads are native to South America and occupy a wide range of habitats from tropical Amazonian rainforests to true savannah to sandy beaches (Rivera et al. 2022; Zug and Zug 1979). They have been introduced to and moved between multiple Caribbean islands, and their complex introduction history is well documented (Easteal 1981; Zug and Zug 1979). All toad lineages that were eventually introduced to Australia via Hawai'i apparently originated in French Guiana and Guyana (Easteal 1981; Slade and Moritz 1998). In at least one area from where toads that founded the Australian introduction were sourced (French Guiana), toads occur in the rainforest and on sandy beaches (DeVore et al. 2021). It is likely that toads from multiple native collection sites were admixed in the Caribbean islands prior to their introduction to Hawai'i, potentially increasing their genetic diversity (e.g., Kolbe et al. 2004) and thus their adaptive potential. In 1935, 101 cane toads were taken from Honolulu, Hawai'i to Gordonvale, Queensland in northern Australia, bred, and their offspring were released, marking one of the worst anthropogenic biological control actions in human history (Turvey 2013). There was only a single introduction to Australia and no hybridisation with native fauna has been reported.

Recent phylogenetic and ecological studies suggest that two ecotypes, rainforest and coastal, are present in native French Guianan and Guyanese populations (DeVore et al. 2021; Mittan‐Moreau et al. 2022). Toads from tropical rainforest sites in French Guiana differ from those in coastal sites in terms of mean snout‐to‐vent length (SVL), mass, and body condition (DeVore et al. 2021). Also, movement patterns, foraging, and sheltering behaviours differ between coastal and rainforest populations; rainforest toads change shelters more often but travel shorter distances for foraging than do coastal toads (DeVore et al. 2021). These differences may represent local adaptation, but whether they are plastic or genetically encoded is unknown. The mitochondrial genomes of rainforest and coastal toads in French Guiana do not differ (Cheung, Amos, et al. 2024). The nuclear genomes of these two groups have not been explicitly studied, but a phylogenetic study including two rainforest toads did show that these individuals differed from those collected on the coast (Mittan‐Moreau et al. 2022). However, historical records lack the specific localities from where toads were collected in the native range, so it is unknown whether both ecotypes were introduced to Caribbean Islands, and subsequently to Australia.

In nearly a century since their introduction to Australia, cane toads have expanded their range from tropical Queensland (QLD) to arid Western Australia (WA) (Shine 2018). Radio‐tracking of free‐ranging cane toads in Australia demonstrated a substantial increase in daily movement in those from more westerly populations; in QLD, toads moved approximately 20 m per day whereas in WA, they moved more than 200 m per day on average (Shine et al. 2021). Range‐core (QLD) and range‐edge (WA) conspecifics display differences in immune function, dispersal ability, morphology and behaviour (Alford et al. 2009; Brown et al. 2015; Gruber et al. 2018, 2017b; Hudson et al. 2018; Phillips et al. 2010). Common‐garden experiments indicate that some of these phenotypic traits are heritable (Brown et al. 2015; Gruber, Brown, et al. 2017; Hudson et al. 2016, 2018). Additionally, genetic differences exist between range‐core and range‐edge toads in Australia (Selechnik et al. 2019).

Toads experienced aridity gradients in the native and Australian invasive range. In French Guiana, thermal and rainfall conditions between rainforests and the coast differ only subtly but rainforest substrates tend to have more standing water whereas sandy beaches drain quickly and are exposed to frequent and extreme salinity changes (Ducatez et al. 2025), leaving reduced and patchier access to fresh water as compared to the rainforest (DeVore et al. 2021). In Australia, this gradient is more pronounced: tropical rainforests in Queensland are wetter (high mean annual rainfall (above 1000 mm per year) (Meteorology 2025)) than the areas where toads have expanded to the west (fresh water is limited in the dry season [less than 200 mm to none per year] Brown et al. 2011; Brusch et al. 2019). These differences are likely to impact toads, which require frequent access to fresh water (Mathwin et al. 2021). These aridity gradients make cane toads an excellent model to study repeated evolution in response to environmental similarities across their native and invasive ranges.

In this study, we investigated patterns of morphological and genetic differentiation within the cane toad's native range, and genetic patterns in the Australian invasive range. We hypothesised that changes across the invasive range result from repartitioning of native range alleles associated with specific environmental conditions. To do this, we first compared the morphology of native toads from rainforest and coastal regions. We then characterised genetic patterns in the native range and specifically looked for genetic differences between rainforest and coastal toads. Next, we investigated whether invasive toads in Australia appear to originate from rainforest and/or coastal native populations. We then asked whether polymorphisms in invasive‐range toads result from novel or standing genetic variation from the native range. Finally, we investigated whether genetic differences associated with environment in the invasive range mirror those in the native range. This study enhances our understanding of the genetic structure and diversity of cane toads in their native and introduced ranges and provides insights into the role of evolution during biological invasions.

## 2 Methods

### 2.1 Morphological Data Collection and Analysis

**FIGURE 1:**
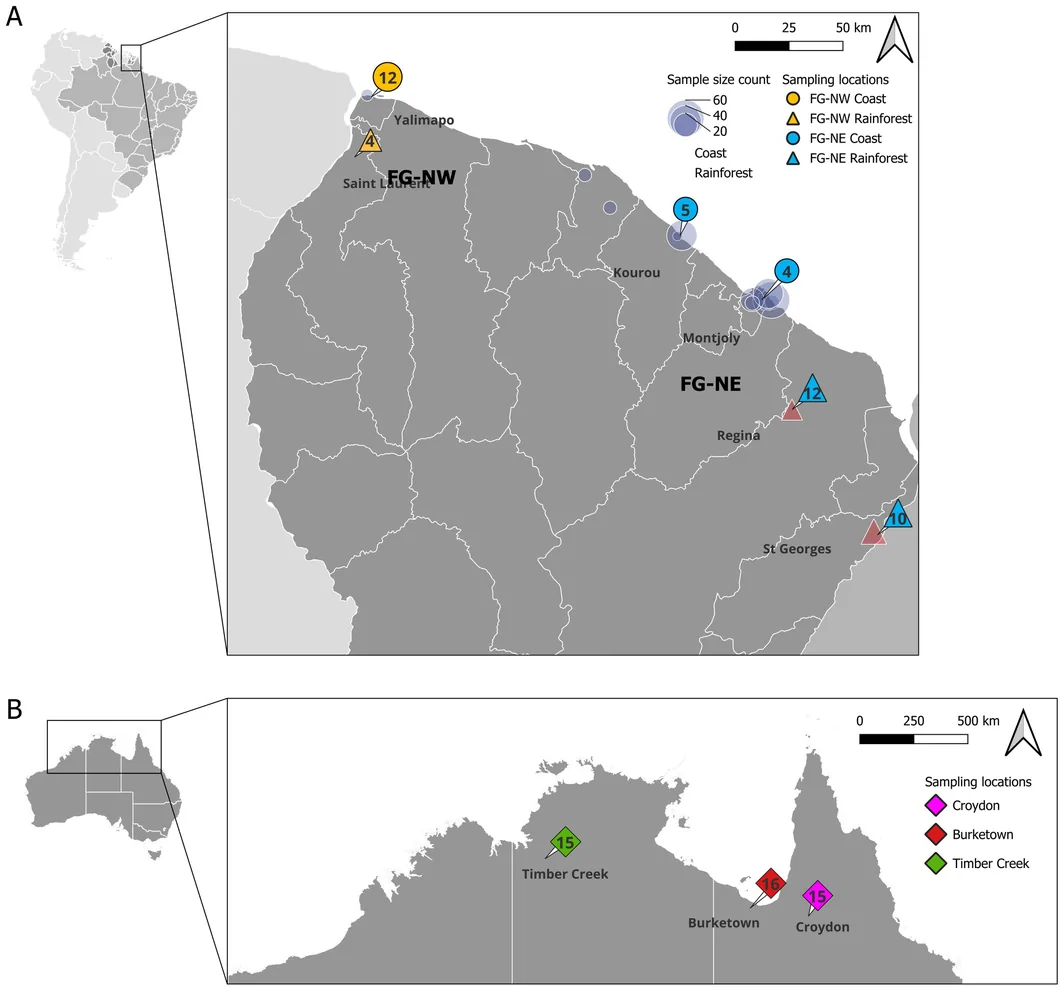
Sampling locations of cane toads ( *Rhinella marina* ) in (A) French Guiana and (B) Australia. Samples for morphological analyses are proportionally labelled using circles and triangles with white outlines (pale red: Rainforest; pale blue: Coast). Samples for DArTSeq collected within close proximity (within 1 km) are grouped and labelled with balloons. Numbers inside the balloons indicate the genetic sample size for that site. Different colours (blue: FG‐NW; yellow: FG‐NE) refer to two separate rainforest versus coastal contrasts (triangle: Rainforest; circle: Coast) in French Guiana.

We hand‐collected all toads detected with a headlamp during night walks across the northeast region of French Guiana, including rainforest and coastal sites. A total of 50 toads from rainforest areas and 236 toads from coastal areas were collected (Figure 1A, Table S1). All rainforest sites were at least 20 km from the coast. Sites were classified as coastal if they were located within 5 km from the coast. We used Vernier callipers (+/1 0.1 mm) to measure snout‐vent length (SVL), arm length (humerus, radioulna), and leg length (tibiofibula, femur) of each toad. Sex was determined using secondary sexual characteristics (Kelehear and Shine 2019). A chi‐squared test was used to compare sex ratios between rainforest and coastal sites.

For morphological analysis, due to the small number of females found in the NE rainforest (*N* = 6), we focused on data from males (*N* = 210) only. To normalise the data, all morphometrics including SVL were ln‐transformed. To assess body size differences between environments, we compared ln SVL between toads from different environments with a Wilcoxon rank‐sum test and a Fligner–Killeen test. To compare limb morphology between environments, we performed linear regressions for arm length and leg length (ln‐transformed) individually, with environment as the predictor and ln SVL as a covariate to standardise the measurement to body size. These models were run using the *lm* function in the R package *stats*. We extracted residual scores from the ln‐ln regressions and tested for environmental differences with Wilcoxon rank‐sum tests.

### 2.2 Genetic Data

We collected cane toad toe clips and preserved them in ethanol from five sites across the northwest (NW) and northeast (NE) of French Guiana, including rainforest and coastal sites (female, *N* = 13; male, *N* = 26; juvenile/unknown sex, *N* = 8; Figure 1A, Table S2). In northern Australia, we collected muscle tissue from toads collected at Croydon (range‐core, wetter), Burketown (range‐core, drier) and Timber Creek (range‐edge, drier) (female, *N* = 24; male, *N* = 22; Figure 1B, Table S2). The Australian toads were collected immediately following euthanasia and preserved in RNALater (QIAGEN) before being transported to permanent storage in −80°C.

We extracted genomic DNA (gDNA) from toe and muscle tissue using a Puregene Tissue Kit (QIAGEN) according to the manufacturer's protocol. The extracted gDNA was quantified using a Qubit dsDNA Kit (Thermo Fisher Scientific). Two μg of gDNA of each sample were submitted for high‐throughput DNA genotyping using DArTSeq technology at Diversity Arrays Technology Pty Ltd. (Canberra, Australia). Samples were digested using *PstI* and *SphI* restriction enzymes and compatible adaptors were ligated to the overhangs. Digested DNA was then amplified in 30 rounds of PCR under the following reaction conditions: 94°C for 1 min; 30 cycles of 94°C for 20 s, 58°C for 30 s and 72°C for 45 s; 72°C for 7 mins. Equimolar amounts of amplified product were combined and subjected to 100 cycles of single reads sequencing on NovaSeq X+ (Illumina).

Raw single‐end sequencing reads were quality checked using FastQC v0.12.1. (http://www.bioinformatics.babraham.ac.uk/projects/fastqc/). Low quality bases, adapters, the first 20 bases from 5′ end and the last 5 bases from 3′ end were removed from each read using fastp v0.23.4 (Chen et al. 2018) using the following commands: ‘‐‐trim_front1 20 ‐‐trim_tail1 5 ‐‐cut_right ‐‐cut_mean_quality 30’. Cleaned reads were then visually assessed by FastQC v0.12.1 (Andrews 2010) and MultiQC v1.17 (Ewels et al. 2016). Reads for each individual were mapped to aRhiMar1.3 genome assembly (GCA_900303285.2; Cheung, Rollins, et al. 2024) using bwa v2.2.1 (Vasimuddin et al. 2019) with default parameters and were then sorted by genomic coordinates using SAMtools v1.20 (Danecek et al. 2021). Furthermore, only mapped reads with mapping quality at least 20 were retained for downstream analyses. Individuals with less than 70% mapping rate were discarded. Details of raw and filtered reads are provided in Table S4.

The filtered BAM files were used for variant calling using FreeBayes v1.3.6 (Marth 2012). We only retained biallelic SNPs and filtered the variants using VCFtools v0.1.14 (Danecek et al. 2011) according to these parameters: minimum QUAL of 30, minimum read depth of 5, maximum read depth of 50, minimum mean read depth of 5, and minor allelic count of 5. We followed the recommendation from Hemstrom et al. (2024) to perform a global filtering on 70% of missingness and population‐based filtering on 70% in each population (native and invasive) and found that the overall population genetics matrices were highly similar between two filtering scenarios. Therefore, we used the variant‐calling file from the global filtering regime for downstream analyses. A total of 58,987 SNPs remained (hereafter referred to as the ‘Full’ dataset).

### 2.3 Population Genetic Analyses

We estimated the number of genetic clusters (*K*) among all samples in the Master dataset using sNMF (Sparse Nonnegative Matrix Factorisation) (Frichot et al. 2014) implemented in R package *LEA* (Frichot and François 2015). We tested *K* = 1–10, with 100 repetitions for each *K*. We selected the run with the lowest entropy values to identify the best *K*. To examine the robustness of sNMF to regularisation parameters (*α*), we ran analyses with *α* = 10, 100 and 1000. The lowest entropy scores were obtained with *α* = 100 (Table S5).

To investigate the population structure of both native and invasive cane toads, we used Principal Component Analysis (PCA) implemented in R package *PCAdapt*, which performs well when data from admixed individuals are analysed (Luu et al. 2017; Privé et al. 2020). We used the Full dataset for all values of *K* between 1 and 10 and the best *K* value was selected based on Cattell's rule as the optimum number of axes retained by scree plot. The scatter plot was created in R package *ggplot2* (Wickham 2016) with the first three components. Genetic diversity measures (observed heterozygosity [*Ho*], expected heterozygosity [*He*]) and pairwise genetic differentiation (*Fst*) were calculated using the *populations* function in Stacks v2.65 (Catchen et al. 2013), and allelic richness (*A*_*r*_) was calculated using R package *hierfstat* (Goudet 2005).

To investigate the role of geographic factors in shaping spatial genetic differentiation in the native populations, we subsetted all native samples from the Full dataset, referred to as the ‘Native’ dataset hereafter. We calculated isolation by distance (*IBD*) for each genetic cluster determined by PCA and sNMF. Geographical distances were calculated by Euclidean distance and genetic distances were measured by pairwise *Fst* between clusters. The correlation between geographical distance and genetic distance was evaluated by multiple matrix regression with randomisation (MMRR) with 10,000 permutation procedures (Wang 2013) using R package *ecodist* (Goslee and Urban 2007; Oksanen et al. 2023).

### 2.4 Relationship Between Morphological and Genetic Data

We investigated the relationship between SVL and the eigenvalues from the PC axis that separated individuals from rainforest versus coast sites based on their genetic data using MMRR with 10,000 permutations. Pairwise geographical distance was included in the regression to control the effect of strong population structure within the native range. FG‐NW rainforest samples were excluded because we lacked data on the associated morphological measures.

### 2.5 Selection Analyses

To investigate the loci associated with environments in the native range and invasive range in Australia, we used BayPass v2.41 (Olazcuaga et al. 2020) to identify: (1) SNPs that separated toads collected from the rainforest versus coast in French Guiana (‘outlier set 1’) and (2) SNPs that separated invasive range toads from Croydon (most easterly site) versus Timber Creek (most westerly site) (‘outlier set 2’). Because we identified substructure at each site in the invasive range (see results below), we also conducted post hoc testing to investigate whether these subgroupings were driven by sequencing artefacts or sex, and examined the SNPs that formed these subgroups (‘outlier set 3’).

BayPass is based on the BayEnv (Gunther and Coop 2013) model and aims to identify genetic markers associated with population‐specific covariables, accounting for population structure caused by demographic history. For ecotype divergence, we used the environments (outlier set 1; Native: Coast = 1 and Rainforest = −1) or sampling sites (outlier set 2; Invasive: Croydon = −1 and Timber Creek = 1) as the binary covariable. For investigation of substructure within Australian sites (post hoc comparison), we used the substructure groupings as the binary covariable (AU_Group1 = 1 and AU_Group2 = −1). We calculated the association of SNP with contrast covariates as contrast statistics *C*_*2*_ and Bayes Factor (*BF*). To calibrate the *C*_*2*_ statistics with non‐normalised *p*‐values, we generated pseudo‐observed datasets (*POD*s) from the calculated correlation matrix (Ω) via simulation of 10,000 SNPs. The top 1% of *C*_2_ in the PODs was set as the threshold for the actual dataset to determine significant associations. We also checked the convergence and reproducibility of MCMC estimates by repeating BayPass three times using different seeds. We used Jeffrey's rule to determine the strength of the *BF* association (*dB*): strong (10 ≤ *dB* < 15), very strong (15 ≤ *dB* < 20) and decisive (*dB* ≥ 20), according to the BayPass v2.41 manual, and with *C*_*2*_ statistics above the threshold. Outliers with *BF* ≥ 10 in all three independent BayPass runs were kept for downstream analyses.

### 2.6 Outlier SNP Comparisons Across the Native and Invasive Ranges

The founders of the Australian introduction could have carried the alleles that confer a selective advantage in either wetter or drier environments, or both, due to the complex introduction history. If alleles from both environments were introduced to Australia, their prevalence across the invasive range should change along the environmental gradient from the original Queensland introduction sites towards more arid regions to the west (e.g., loci that confer a selective advantage in drier environments will increase in frequency with increasing distance from the introduction sites). To assess whether the allelic frequency changes across the aridity gradient in the native range were associated with those across the aridity gradient in the invasive range, we first used outlier set 1 to extract the reference allelic frequency (e.g., with respect to the reference genome of an invasive toad) of the outliers identified from BayPass in all native and invasive samples according to the population assignment used in BayPass using VCFtools v0.1.14. The degree of change in allelic frequency was obtained by subtracting the allelic frequency between groupings (between the rainforest and coastal populations in the native range and between Croydon to Timber Creek populations in the invasive range). We then calculated Spearman's rank correlation between frequency differences at each outlier. This method allowed us to investigate whether loci associated with environmental differences in the native range displayed similar patterns in the invasive range. We plotted changes in allele frequencies of the SNPs identified in the BayPass analysis from the native ranges versus those from the invasive range, where we expected that if increases in allele frequencies occurred across both ranges, the allele should appear in the top right quadrant of the plot. Likewise, if an allele decreased in frequency across both ranges, it should appear in the bottom left quadrant. Alleles that appeared in the top left and bottom right of the plot would indicate that they changed in different directions across the two ranges. We also plotted the BayPass SNPs in PCA space to determine if alleles from similar environments from both ranges clustered together.

We repeated these analyses with outlier set 2 to determine if loci that appear to be under selection in Australia also appear to cluster by environment in the native range. Here, we extracted the change in allele frequencies for these SNPs as described above and plotted the reference allele frequency for each location. If patterns of selection were similar across ranges, we expected to see similar allelic patterns at these loci. We also plotted these SNPs in PCA space to determine if alleles from similar environments from both ranges clustered together.

### 2.7 Annotation of Outlier SNPs and Associated Genes

To identify genes associated with putative loci under selection in rainforest or coastal environments and between range‐core and range‐edge populations in the invasive range, we used BEDTools v2.30.0 (Quinlan 2014) to extract flanking regions 10 kb away from the outlier SNPs. Gene names were extracted from the DAVID database (Sherman et al. 2022). To see whether genetic patterns in the invasive range in Australia repeated the patterns in the native range, we compared the putative SNPs and nearby genes identified in selection analyses in the native range (outlier set 1 and associated genes) to those identified in the invasive range (outlier set 2 and associated genes).

## 3 Results

### 3.1 Morphology in the Native Range

**FIGURE 2:**
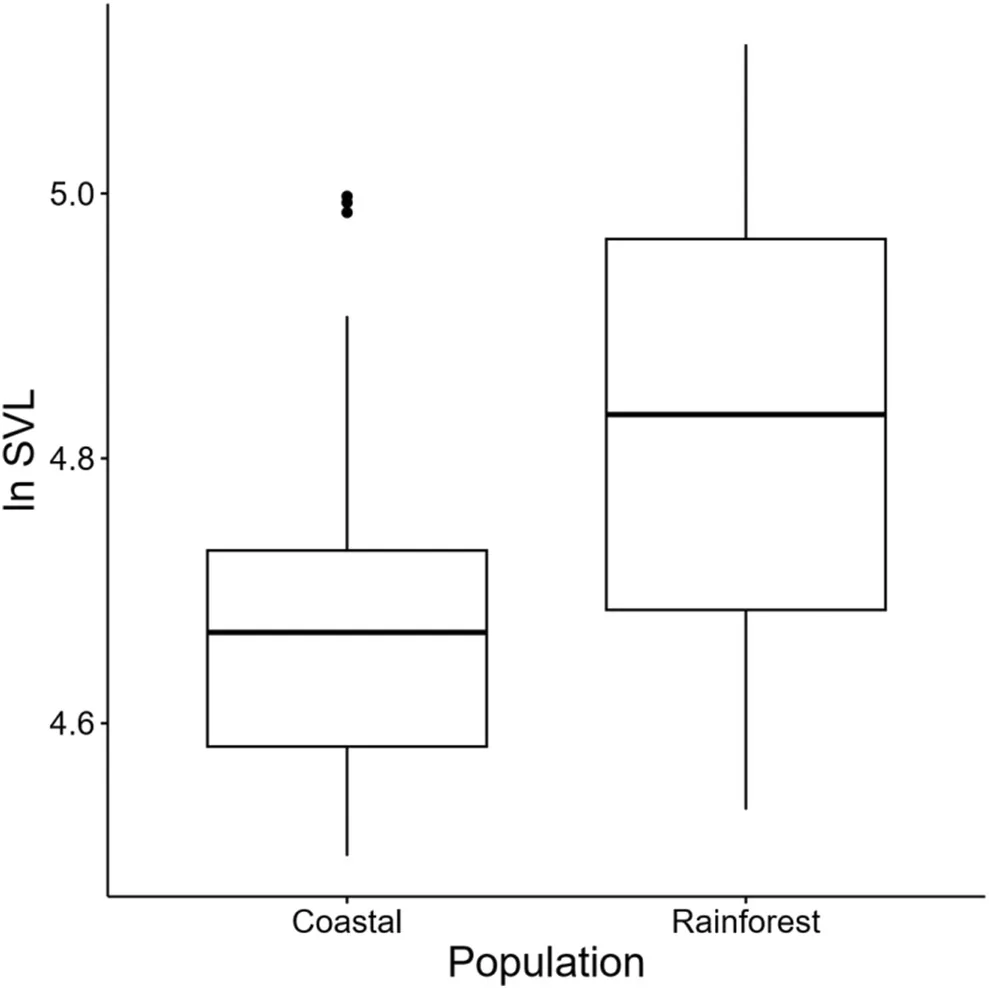
Boxplot comparing the snout‐vent length (SVL) of 210 native male cane toads ( *Rhinella marina* ) collected in coastal and rainforest environments in the northeast of French Guiana. Boxes represent interquartile range (IQR); horizontal line represents the median; whiskers extend to values within 1.5 × IQR, and points beyond the range are shown as outliers.

Both sex ratio and mean body size differed between rainforest and coastal toads in the native range. Rainforest sites had more males (6.7 males per female) than coastal sites (2.2 males per female) (Pearson Chi‐squared test = 4.345; *p* = 0.037). Males from rainforest sites had greater mean SVL (rainforest male mean SVL = 4.82; coastal male mean SVL = 4.67; *p* < 0.0001) and greater variance in body size (rainforest male SD = 0.107; coastal male SD = 0.161; Fligner‐Killeen Chi‐squared = 16.988, df = 1, *p* < 0.0001; Figure 2) than those from coastal sites. None of the relative lengths in humerus, radioulnar, femur and tibia were significantly different between rainforest and coastal male toads (Figure S1, Table S3).

### 3.2 Population Genetic Patterns

After sequencing, we obtained an average of 7.08 million reads per individual before quality filtering, and 6.92 million reads per individual after (Table S4). After mapping, we removed three individuals due to low mapping rates, leaving 90 individuals from the native (*N* = 44) and invasive (*N* = 46) ranges for analyses. A total of 58,987 SNPs with no more than 30% missing data, a minor allele count of 5, and a maximum read depth of 50 were retained in the Full dataset after variant filtering.

**FIGURE 3:**
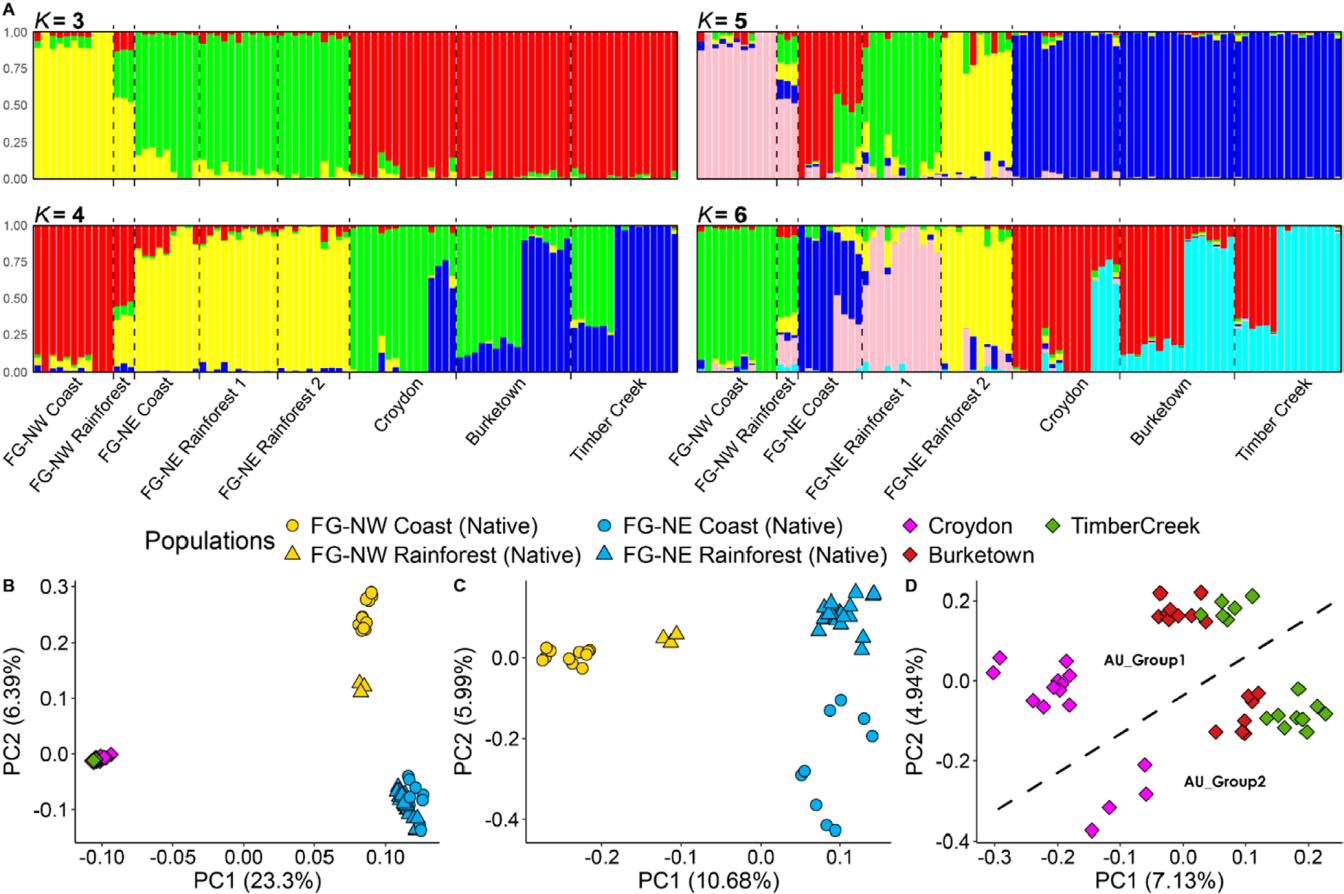
Population structure of native (*N* = 44) and invasive (*N* = 46) cane toads ( *Rhinella marina* ). (A) sNMF (sparse non‐negative matrix factorization) analysis computed in LEA for *K* = 3–6, based on 58,897 SNPs. Each individual is represented by a vertical bar and ordered according to sampling locations shown on the *x*‐axis. (B) Principal Component Analysis (PCA) using all toads, (C) PCA based on a subset of 44 native toads sampled in French Guiana, and (D) PCA based on a subset of 46 invasive toads sampled in Australia (subgroups separated by diagonal). Brackets indicate percentage variance explained by each PC. In (B–D), coloured symbols (yellow: French Guiana, Northwest (FG‐NW); blue: French Guiana, Northeast (FG‐NE); purple: Croydon; red: Burketown and green: Timber Creek) refer to sampling locations shown in Figure 1. Native toads from the rainforest and coast are denoted by triangles and circles, respectively. All invasive toads are denoted by diamonds.

We found population structure within the native and invasive ranges. In the sNMF analysis, the lowest entropy value was obtained in *K* = 3 (Table S5). French Guiana (native range) samples differed from Australian (invasive range) samples (Figure 3A, *K* = 3). Within French Guiana, individuals from FG‐NW formed a separate genetic cluster with some degree of admixture from those from FG‐NE. FG‐NW Rainforest individuals were differentiated from FG‐NW Coastal individuals. For *K* = 4, two genetic groups were identified in Australia, with their relative proportions shifting with longitude. Higher values of *K* revealed subtle substructure in the native range. For *K* = 5, substructure was noticeable within FG‐NE. Individuals from the FG‐NE Rainforest were separated from those from FG‐NE Coast. Within FG‐NE Rainforest, the two sampling sites differed.

Genetic differentiation between individuals sampled in rainforest versus coastal environments in the native range was evident in the PCA plots (Figure 3B,C, Table S6). In the PCA containing all individuals, native and invasive cane toads formed distinct genetic clusters, congruent with the sNMF analysis in *K* = 3 (Figure 3A). PC1 separated native from invasive toads and explained 23.3% of the variance. In the PCA including only native range toads, FG‐NE and FG‐NW were separated along the PC1 axis, which explained 10.7% of the variance (Figure 3C, region denoted by colour). Rainforest and coastal samples in French Guiana were also separated in Figure 3C (environment denoted by shape). We detected a significant relationship between the geographical distance between collection sites in French Guiana and the genetic distance (*β* = 7.25, *p* = 0.0001) using the MMRR (Figure S2).

In the invasive range, individuals from the more westerly sites (Burketown and Timber Creek) clustered together, while Croydon samples were distinct (Figure 3D). The PC2 axis divided individuals from all three sites into two discrete clusters; the admixture plot also indicated this separation (Figure 3A, *K* = 4 and 6; this separation was investigated with post hoc analyses described below). However, samples from the three invasive sampling sites were distinct in the PC1 vs. PC3 plot (Figure S3).

Levels of genetic differentiation (pairwise *Fst*) between French Guiana and Australia ranged from 0.130 to 0.151 (Table 1). Sites within FG‐NW and FG‐NE regions were more similar to each other (FG‐NE pairwise *Fst*: 0.020–0.031; FG‐NW pairwise *Fst*: 0.023) than were those between regions (pairwise *Fst*: 0.056–0.063), except for the FG‐NW Rainforest (pairwise *Fst*: 0.027–0.032). Toads from the invasive sampling sites exhibited lower levels of differentiation (pairwise *Fst*: 0.014–0.027), despite greater geographic distances between them compared to the native range sites. The *Ho*, *He* and *Ar* indicated that native range populations were more genetically diverse than invasive populations (Table 2). The FG‐NW Rainforest had slightly lower genetic diversity measures than the rest of the native sites, likely an artefact of low sample size (*N* = 3), which can affect these estimates (Allendorf et al. 2022). Within invasive sites, most of the diversity estimates decreased with distance from the site of introduction in Australia (from Croydon moving westward to Timber Creek) (Table 2).

**TABLE 1:** Pairwise *Fst* comparison between toads ( *Rhinella marina* ) collected at sampling sites within (A) French Guiana and (B) Australia.

	FG‐NW rainforest	FG‐NE coast	FG‐NE rainforest 1	FG‐NE rainforest 2	Croydon	Burketown	Timber creek
FG‐NW Coast	0.023	0.059	0.056	0.063	0.142	0.147	0.151
FG‐NW Rainforest		0.029	0.027	0.032	0.133	0.137	0.147
FG‐NE Coast			0.027	0.031	0.142	0.148	0.151
FG‐NE Rainforest 1				0.020	0.130	0.134	0.137
FG‐NE Rainforest 2					0.139	0.144	0.147
Croydon						0.018	0.027
Burketown							0.014

**TABLE 2:** Population statistics of cane toad ( *Rhinella marina* ) populations sampled in French Guiana and Australia.

	N	*Ar*	*Ho*	*He*
FG‐NW Coast	11	1.212 ± 0.001	0.201 ± 0.001	0.202 ± 0.001
FG‐NW Rainforest	3	1.205 ± 0.001	0.194 ± 0.001	0.157 ± 0.001
FG‐NE Coast	9	1.239 ± 0.001	0.214 ± 0.001	0.224 ± 0.001
FG‐NE Rainforest 1	11	1.231 ± 0.001	0.214 ± 0.001	0.221 ± 0.001
FG‐NE Rainforest 2	10	1.229 ± 0.001	0.211 ± 0.001	0.216 ± 0.001
Croydon	15	1.136 ± 0.001	0.13 ± 0.001	0.131 ± 0.001
Burketown	16	1.130 ± 0.001	0.129 ± 0.001	0.126 ± 0.001
Timber Creek	15	1.122 ± 0.001	0.12 ± 0.001	0.118 ± 0.001

Abbreviations: *Ar*, allelic richness; *He*, expected heterozygosity; *Ho*, observed heterozygosity; *N*, number of samples.

### 3.3 Relationship Between Genetics and Morphology

After accounting for the geographical genetic structure using MMRR, we found no significant relationship between individuals' scores on Figure 3's PC2 axis (based on genetic data that separated FG‐NE rainforest toads from FG‐NE coastal toads) and SVL (*β* = −0.172, *p* > 0.05) (Figure S4).

### 3.4 Selection Analyses

If, in the native range, loci identified as under selection in wetter versus drier environments existed, and these loci were retained after translocation, we expected to observe sorting of these loci along the invasion trajectory in Australia (i.e., a higher prevalence of wet environment associated alleles in range‐core populations and a higher prevalence of dry environment associated alleles in range‐edge populations). To investigate this possibility, we utilised BayPass to identify SNPs correlated with the environments in the native range and in Australia. For each outlier set, we only considered SNPs that were consistently identified as outliers in three BayPass MCMC runs.

**FIGURE 4:**
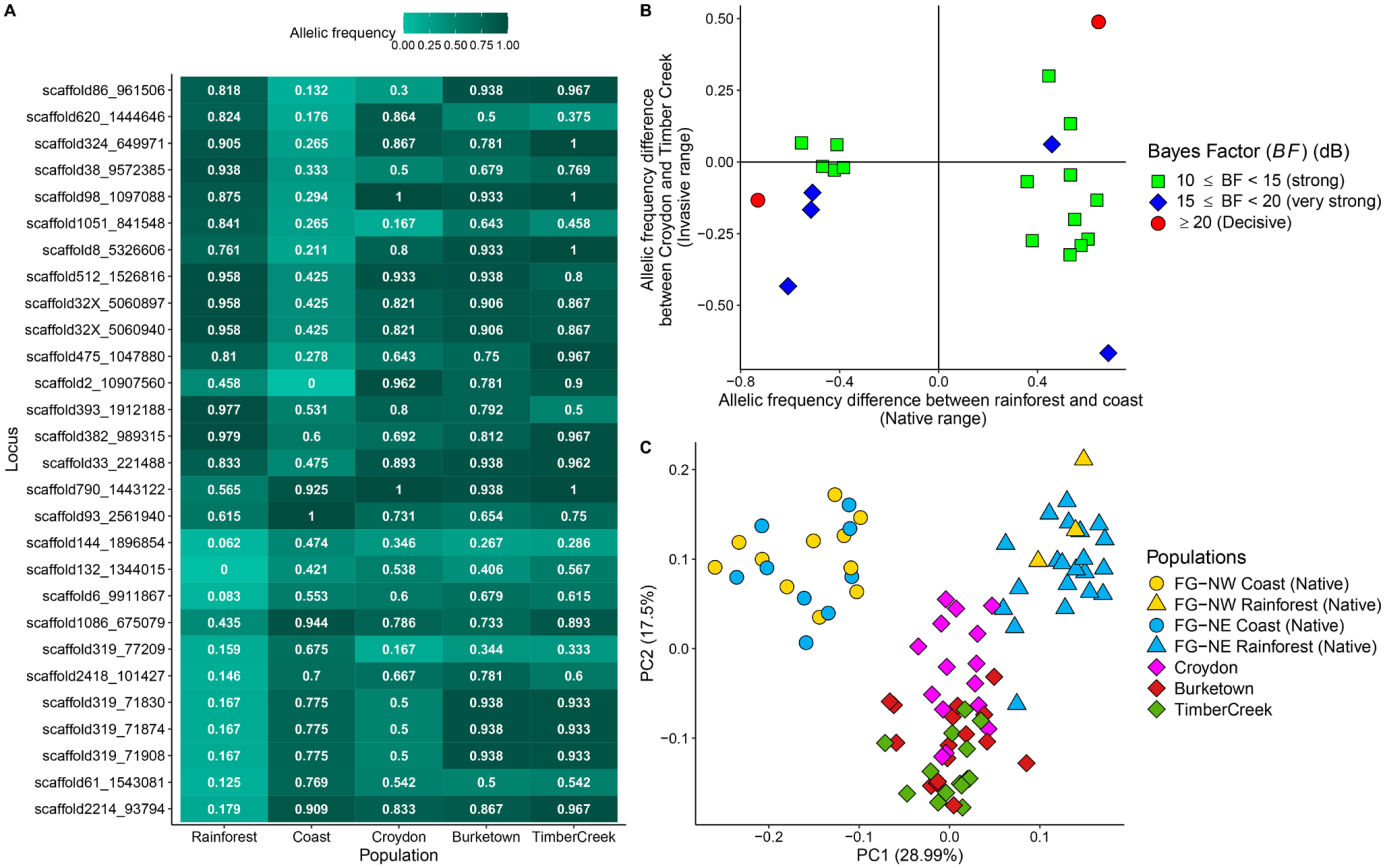
Analysis of loci putatively under selection between native rainforest and coastal toads ( *Rhinella marina* ) sampled in French Guiana (outlier set 1). (A) Allele frequencies of loci under selection. Frequency is calculated based on the reference allele in the original reference genome of an invasive toad. Only outlier SNPs that are variable in the invasive range are shown. Heatmap indicates the absolute allelic frequency of the populations, ordered by the difference between the rainforest and coastal populations in the native range. (B) Relationship between the native and invasive toad allele frequencies of outlier SNPs. (C) Principal Component Analysis of native and invasive toads at outlier SNPs (excluding fixed SNPs in the invasive range). Native rainforest (triangles) and coastal toads (circles) were separated by the PC1 axis.

We found a total of 71 putative outlier SNPs when contrasting the native toads from rainforest versus coastal sites (outlier set 1; *BF* score—strong: 52, very strong: 16; and decisive: 3). We identified 49 genes within 10 kb flanking regions of these SNPs. Of these, six were uncharacterised or novel genes and 43 were annotated genes (Table S7). Forty‐three of these loci were completely fixed in the invasive range (Figure S5) and 13 out of 28 variable loci (46.4%) showed a higher prevalence of putatively dry environment associated loci in invasive range‐edge populations than invasive range‐core populations (Figure 4A). These loci were near six annotated genes (phenylalanyl‐tRNA synthetase 2, mitochondrial (*FARS2*), neuron navigator 3 (*NAV3*), keratin 20 (*KRT20*), cadherin 13 (*CDH13*), peroxisomal biogenesis factor 11 beta (*Pex11b*) and HID1 domain containing (*hid1*)). Some SNPs increased across both ranges (top right quadrant of Figure 4B) or decreased across both ranges (bottom left quadrant of Figure 4B). However, over all 71 SNPs, we found no significant relationship between allelic frequency changes in the native versus invasive ranges (Spearman's = 0.094, *p* = 0.655) (Figure 4B). The PCA created from these 71 putative outlier SNPs indicated that the invasive toads did not cluster with native toads from similar environments, suggesting differences in patterns of selection across these ranges (Figure 4C).

**FIGURE 5:**
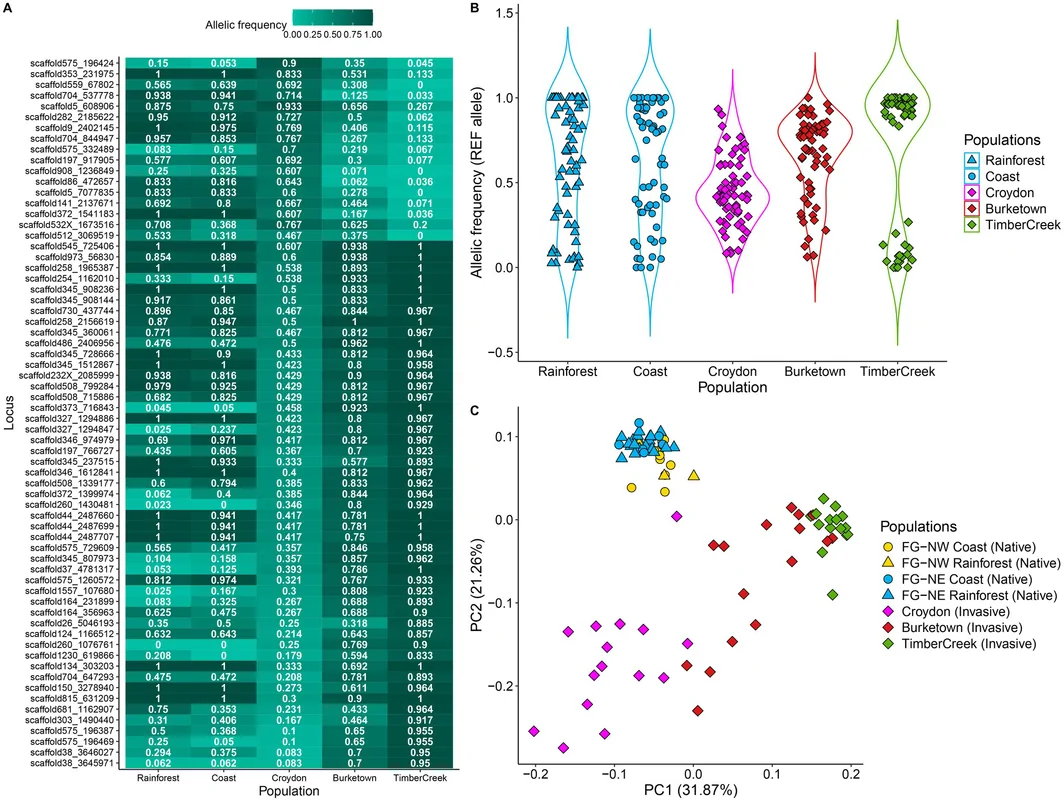
Analysis of loci putatively under selection between eastern (Croydon) and western (Timber Creek) toads ( *Rhinella marina* ) sampled in Australia (outlier set 2). (A) Allele frequencies of loci under selection in Australia. Frequency is calculated based on the reference allele in the original reference genome of an invasive toad. Heatmap indicates the absolute allelic frequency of the populations, ordered by the difference between Croydon and Timber Creek populations in the invasive range. (B) Relationship between the native and invasive toad allele frequencies. (C) Principal Component Analysis of native and invasive toads at outlier SNPs (excluding fixed SNPs in the invasive range). Sampling sites in the invasive range were separated on the PC1 axis.

We investigated putative SNPs under selection in the invasive range by comparing genetic data from Croydon (easternmost site) and Timber Creek (westernmost site). We identified a total of 66 putative outliers (outlier set 2; *BF* score—strong: 55, and very strong: 11). We found 51 genes within 10 kb flanking regions of these outliers. Of these, five were uncharacterised or novel genes and 46 were annotated genes (Table S8). Only 12 out of 66 (18.2%) were fixed in both rainforest and coastal toads in the native range (Figure 5A). In Timber Creek (westernmost site), the allele frequencies for outlier set 2 had more extreme values than all other sites (Figure 5B). However, the coastal samples from the native range did not display this pattern. The PCA constructed using these SNPs did not separate native rainforest and coastal toads, nor did the native toads cluster with individuals from the invasive sites (Figure 5C).

In the post hoc analysis of the substructure we found within Australian sites (Figure 3D, above and below the diagonal), we identified 274 putative outliers (outlier set 3; *BF* score—strong: 31, very strong: 114, and decisive: 129). We found that the minor allele frequency of most SNPs in outlier set 3 was at least 0.3 which indicated these were unlikely to be sequencing artefacts. Additionally, the subgroups did not segregate by sex (Figure S6). We found 184 genes within 10 kb flanking regions of these outliers. Of these, 12 were uncharacterised or novel genes and 162 were annotated genes (Table S9). In the PCA constructed with these outliers, native samples did not cluster with either of the subgroups identified across Australian invasive sites. In addition, these outliers did not separate native samples by environment (Figure S7).

We found no overlap of outlier SNPs nor associated genes between the native and invasive range (outlier set 1 versus outlier set 2; outlier set 1 versus outlier set 3).

## 4 Discussion

### 4.1 Native Rainforest and Coastal Toads Differ Morphologically

We found significant differences in sex ratio and body size between rainforest and coastal toads in the native range. The ratio of males to females in the native rainforest sites (7.3 per female) was much higher than in the native coastal sites (2.2 per female). This extreme sex bias in our sample of the rainforest area precluded our ability to compare females in morphological analyses. Although cane toad habitat use is sex‐biased (males more closely associated with waterbodies and females more closely associated with drier areas; González‐Bernal et al. 2015), in our sampling of French Guiana, both wetter and drier areas were included. Our analysis of males from rainforest versus coastal areas showed morphological differences in mean SVL but not in relative limb lengths (Figure 2 and Figure S1). Body size differences are interesting, given the relatively short distance between these sites (approximately 25 km between the closest rainforest and coastal site). It is unclear why rainforest toads tend to be larger but may be due to differences in age or life history strategies. We did not find a relationship between the SNPs that separated rainforest and coastal toads and their SVL (Figure S4). This indicates that either these SNPs are not predominantly related to body size, or that our sample size of individuals for which we had genetic and morphological data was too small to capture this relationship.

### 4.2 Population Structure in the Native Range and the Origins of the Invasive Population

Despite the impact of cane toads on the Australian fauna (Shine 2010) and evidence of adaptation in invasion front toads in tropical Australia, the genetics of cane toads in their native range have barely been studied (but see Selechnik et al. 2019). We found moderate levels of population genetic structure within French Guiana (*Fst* ranged between 0.02–0.06), despite short geographic distances between sites (~20–250 km), and low levels of admixture between sites (Figure 3A). We found weak but significant IBD across sites in French Guiana (*r* = 0.10, *p* = 0.01). These results are unsurprising given that native toads have low dispersal rates and only travel 20–180 m daily for foraging (DeVore et al. 2021; Shine et al. 2021). Interestingly, toads from sites within Australia were less genetically differentiated than were those in the native range (*Fst* ranged between 0.01–0.03), despite larger geographic distances (~250–1250 km) between sites. This result is consistent with the much larger daily displacement distances seen in Australian toads versus those in the native range (Shine et al. 2021). Although invasive toads clustered more closely with native rainforest toads and had lower *Fst* values than coastal toads, there has been considerable divergence between the native and invasive range toads (*Fst* ranged between 0.13 and 0.15), no indication of shared ancestry in admixture analyses, and no patterns of shared alleles in SNPs under selection that suggest origin. While the source population(s) remain unresolved, future demographic inference incorporating samples from French Guiana, Guyana and intermediate populations will be necessary to clarify the origin of introduction.

We found consistent levels of within‐site genetic diversity in French Guiana, except for the FG‐NW rainforest site which appeared to have lower levels of diversity. However, this may be an effect of the small sample size from that site. As expected, we found lower levels of genetic diversity in the invasive range in Australia, likely due to founder effects and multiple bottlenecks that preceded the Australian introduction. This finding is supported by previous studies on mitochondrial DNA, microsatellite and transcriptomic analyses comparing native range toads from French Guiana and invasive toads from Australia (Cheung, Amos, et al. 2024; Estoup et al. 2001; Selechnik et al. 2019; Slade and Moritz 1998). Within invasive sites in Australia, neutral genetic diversity decreased with distance from the site of introduction (as has been shown previously: Selechnik et al. 2019).

### 4.3 Selection in the Invasive Range Acts on Standing Genetic Variation, Independent of Native Ecotypic Divergence

The environmental contrasts (coastal versus rainforest) within French Guiana and Australia provide an excellent opportunity to study whether evolution follows the same path when a species is introduced into a new area with similar environmental features as those found in its native range. Genetic divergence is often studied in invasive populations spanning different environmental gradients but there are only a few examples of how selection in the native range impacts an invading population. One such example investigated ecotypic differentiation across lake‐stream contrasts and found patterns were repeatable and predictable during the early stages of invasion of three‐spined sticklebacks ( *Gasterosteus aculeatus* ) in Switzerland (Lucek et al. 2013). Another study showed that a large haploblock carrying adaptive traits was present in the repeated parallel adaptation of an invasive ragweed *Ambrosia artemisiifolia* (Battlay et al. 2023).

The complex translocation history of cane toads and incomplete historical information regarding source locations make this task complicated in our system. For example, it is unknown if both rainforest and coastal toads were brought to the Caribbean Islands from French Guiana. If both ecotypes were introduced to Barbados, they presumably had the opportunity to interbreed prior to being taken to Puerto Rico a century later (assuming a lack of reproductive isolation between them). If interbreeding between ecotypes occurred, then toads carrying alleles from both ecotypes may have been taken to Hawai'i and eventually to Australia. As toads moved across Australia, from tropical rainforest environments in Queensland to hot and arid environments in the Northern Territory and Western Australia, these alleles may have sorted along this gradient. In this case, we might expect that toads from Queensland would carry more alleles from the native range rainforest environment while those more westerly toads in Australia would carry a higher proportion of alleles from the coastal environment. By comparing the allelic frequencies of loci putatively under selection in both the native range (coastal versus rainforest) and the invasive range (easternmost versus westernmost sites in Australia), we aimed to investigate if environmentally associated patterns of selection in the invasive range mirrored those in the native range.

Using BayPass for selection analyses that account for the neutral population structure we observed (Olazcuaga et al. 2020), we identified 71 (native, outlier set 1) and 66 (invasive, outlier set 2) loci putatively under selection. There was no overlap between the outlier loci found in the native and invasive toads and no overlap of closely located genes. The outlier loci from the native toads were mostly fixed in the invasive toads, probably resulting from serial genetic bottlenecks across the invasion trajectory. Of the loci putatively under selection in the invasive range, more than 80% were polymorphic in the native range but they did not differ between native rainforest and coastal toads. This suggests that selection on toads in Australia is mainly underpinned by standing genetic variation from the native range. Of alleles that were fixed in the native range, the new variants identified in Australia could be novel alleles that arose in Australia but are more likely to be alleles that exist in French Guiana or Guyana but were not sampled in our study. The outlier loci identified in Australia approach fixation with distance from the site of introduction, which could result from directional selection as toads arrive in more arid environments or could result from genetic drift occurring across successive founding events. Critically, SNPs identified as under selection in the invasive range did not differentiate rainforest versus coastal toads in the native range. In summary, our results suggest that even when invasive toads sort into similar environments as are found in the native range, selection appears to act on different genetic variants.

### 4.4 Substructure Within Invasive Range

Unexpectedly, we observed two genetic groups within each sampling site in Australia (Figure 3A, *K* = 4, represented by green and blue bars). The proportion of individuals in these groups shifted with distance from the site of introduction (Croydon: blue group = 27%; Burketown: blue group = 47%; Timber Creek: blue group = 60%). This pattern was not reported previously in genetic analysis of toads from these sites (Selechnik et al. 2019). This pattern does not seem to be caused by sequencing artefacts or by differences in sex among the samples. We then wondered if these groups in the invasive range might be representative of coastal and rainforest lineages from the native range, under the assumption that there may be barriers to interbreeding. Our post hoc analyses of the outliers that separate groups in the invasive range did not support this hypothesis (outlier set 3). Native range samples from each ecoregion did not cluster with the invasive subgroups, nor did any SNPs (or nearby genes) overlap between outlier sets 1 (native) and 3 (invasive subgroups). Further investigation of this finding is difficult without a more contiguous version of the genome.

### 4.5 Limitations and Conclusions

Reduced‐representation sequencing data allows researchers to investigate population genomics in a cost‐effective manner, especially in non‐model organisms. We acknowledge that the number of markers (58,987 SNPs) generated in this study only represents a tiny portion of the genome (0.002% of 3.5 Gb). These loci may not be associated with the genes involved in adaptation to different environments, if toads are indeed adapting to environmental gradients. Without a chromosomal‐level genome assembly, we are unable to determine if alleles across these two sets of loci putatively under selection are linked and, if so, how close they are to one another on the chromosome. Haploblocks under selection may result in ecotypic differentiation (e.g., as in invasive weed, Battlay et al. 2023; African honeybee, Wallberg et al. 2017; sunflowers, Todesco et al. 2020; and European aspen, Wang et al. 2018). SNPs that appear to be under selection may contribute to adaptive traits or may simply be proximal to regions of the genome that are under selection. A chromosome‐level genome assembly and whole‐genome sequencing would improve the ability of future research to investigate the genetic basis of differentiation between ecotypes in the native range and between range‐edge and range‐core populations in the invasive range.

A previous study provided limited evidence for ecotypic differentiation in the native range (Mittan‐Moreau et al. 2022). Our results provide additional support for the presence of ecotypes in different environments in French Guiana, both through the analysis of phenotypic and genetic data. Although at least some of the toads taken from French Guiana to establish populations in the Caribbean were likely to be collected from beaches near ports, it now seems likely that toads from rainforest regions were also introduced to the Caribbean. Coastal populations in Guyana are closely related to populations in Puerto Rico and Hawai'i (Mittan‐Moreau et al. 2022), so may also be similar to toads in Australia, but no study to date has included toads from the full invasion trajectory from Guyana/French Guiana, throughout the Caribbean, Hawai'i, and across Australia. To better understand if patterns of selection are repeatable across introductions, better sampling along the entire invasive trajectory would be useful.

## Author Contributions

**Kelton Cheung:** conceptualization (equal), data curation (lead), formal analysis (lead), methodology (lead), visualization (lead), writing – original draft (lead), writing – review and editing (lead). **Richard J. Edwards:** conceptualization (equal), formal analysis (supporting), methodology (supporting), supervision (lead), writing – original draft (equal), writing – review and editing (equal). **Jayna L. DeVore:** data curation (equal), methodology (supporting), writing – original draft (supporting), writing – review and editing (supporting). **Simon Ducatez:** data curation (equal), methodology (supporting), writing – original draft (supporting), writing – review and editing (supporting). **Cameron M. Hudson:** data curation (equal), methodology (supporting), writing – original draft (supporting), writing – review and editing (supporting). **Richard Shine:** funding acquisition (lead), methodology (supporting), project administration (equal), writing – original draft (supporting), writing – review and editing (supporting). **Lee A. Rollins:** conceptualization (equal), funding acquisition (lead), methodology (equal), project administration (lead), supervision (lead), writing – original draft (equal), writing – review and editing (equal).

## Funding

This work was supported by the Australian Research Council (DP190100507).

## Conflicts of Interest

The authors declare no conflicts of interest.

## Supporting information


**Figure S1:** Residuals of the ln‐ln regression of arm length (humerus, radioulna) and length (tibiofibula, femur) with ln snout‐vent length (SVL) as covariate between male native cane toads ( *Rhinella marina* ) sampled in rainforest versus coastal environments in French Guiana.
**Figure S2:** Isolation by distance (IBD) based on a panel of 58,987 SNPs from 44 native cane toads ( *Rhinella marina* ) sampled from French Guiana.
**Figure S3:** Population structure of native and invasive cane toads ( *Rhinella marina* ). Principal Component Analysis based on a panel of 58,987 SNPs from (A) 44 native cane toads (PC1, PC3 axes) and (B) 46 invasive cane toads (PC1, PC3 axes).
**Figure S4:** Relationship between snout‐vent length (SVL) and the principal component scores from PC2, Figure 3C, which is based on genetic data that separated FG‐Northeastern (NE) Rainforest toads from FG‐NE coast toads ( *Rhinella marina* ) (Mantel *r* = −0.114, *p* > 0.05). FG‐Northwestern (NW) toads were excluded due to the absence of morphological measurements. Native toads from the rainforest and coast are denoted by triangles and circles, respectively.
**Figure S5:** Analysis of loci putative under selection between native rainforest and coastal toads ( *Rhinella marina* ) sampled in French Guiana. Frequency was calculated based on the reference allele in the reference genome of an invasive toad. Only outlier SNPs that are fixed in the invasive range are shown. Heatmap indicates the absolute allelic frequency of the populations, ordered by the difference between the rainforest and coastal populations in the native range.
**Figure S6:** Principal component analysis based on a subset of 46 invasive cane toads ( *Rhinella marina* ) sampled in Australia, coloured by sample sexes.
**Figure S7:** Principal component analysis based on a panel of 274 SNPs that separate invasive range cane toads ( *Rhinella marina* ) in each invasive range sampling site (see Figure 2D).
**Table S1:** Summary information of cane toads ( *Rhinella marina* ) used for morphological analysis in this study.
**Table S2:** Summary information of cane toads ( *Rhinella marina* ) used for genetic analysis in this study.
**Table S3:** Regression models showing the morphological (arm and leg length) differentiation between rainforest and coastal male cane toads ( *Rhinella marina* ) sampled in French Guiana. The main value and the number in parentheses represent regression coefficients and standard errors respectively. Coastal populations were considered as reference, so that a positive coefficient of POPRainforest means that rainforest toads had larger limbs (after correcting for SVL).
**Table S4:** Summary information of sequencing data for *Rhinella marina* used in this study, before and after variant filtering.
Table **S5**. sNMF cross‐entropy values in *α* = 10, 100 and 1000.
**Table S6:** Percentage of variance explained by each PC axis by PCAdapt of all 90 native and invasive cane toads, a subset of French Guiana cane toads and a subset of invasive Australian cane toads.
**Table S7:** Putative outliers detected by BayPass between cane toads from the coast and rainforest in French Guiana. Gene ontology terms were assigned using DAVID.
**Table S8:** Putative outliers detected by BayPass between Croydon and Timber Creek in Australia. Gene ontology terms were assigned using DAVID.
**Table S9:** Putative outliers detected by BayPass between AU_group1 and AU_Group2 in Australia. Gene ontology terms were assigned using DAVID.

## Data Availability

The raw genomic sequencing data is available at NCBI BioProject PRJNA1346645 and the code used can be found on GitHub at https://github.com/Keltonc.
