# Supplementary material for: Similar Habitat Gradients Across Native and Invasive Ranges Elicit Divergent Evolutionary Responses
Source: Ecol Evol. 2026 Sep 23;16(9):e74407. doi: 10.1002/ece3.74407 (PMC13601023; doi:10.1002/ece3.74407)
Supplement: Supplementary file 1 [file ECE3-16-e74407-s001.docx]

**Supplemental Information for:**

**Similar habitat gradients across native and invasive ranges elicit divergent evolutionary responses**

Kelton Cheung, Richard J Edwards, Jayna L DeVore, Simon Ducatez, Cameron M Hudson, Richard Shine, Lee A Rollins

**Table of Contents:**

| **Summary of Introduction History** | Page 2 |
| --- | --- |
| **Figure S1** | Page 3 |
| **Figure S2** | Page 4 |
| **Figure S3** | Page 5 |
| **Figure S4** | Page 6 |
| **Figure S5** | Page 7 |
| **Figure S6** | Page 8 |
| **Figure S7** | Page 9 |
| **Table S1** | Page 10 |
| **Table S2** | Page 21 |
| **Table S3** | Page 25 |
| **Table S4** | Page 26 |
| **Table S5** | Page 27 |
| **Table S6** | Page 28 |
| **Table S7** | Page 29 |
| **Table S8** | Page 45 |
| **Table S9** | Page 70 |

## Summary of Introduction History

Cane toads are native to South America but have been introduced to and moved between multiple Caribbean islands; this complex introduction history is well documented (Easteal, 1981; Zug & Zug, 1979). However, all toad lineages that were eventually introduced to Australia apparently originated in French Guiana and Guyana (Easteal, 1981; Slade & Moritz, 1998). The first documented introduction from Guyana to Barbados occurred in approximately 1833. Toads were also taken from Cayenne, French Guiana prior to 1844 and introduced to Barbados via Martinique. In 1844, cane toads were translocated from Barbados to Jamaica. In 1920, fewer than two dozen individuals from Barbados were released at Mayaguez in western Puerto Rico. A second introduction to Puerto Rico occurred in 1923-1924, when 40 cane toads were taken from Jamaica to San Juan in the northeast. In 1932, 149 individuals were transported from San Juan to Oahu, Hawai’i. It seems unlikely that the two Puerto Rico introductions had merged prior to the Hawai’ian introduction (Easteal, 1981). Given this complex history, it is likely that toads from multiple native populations were admixed in the Caribbean islands prior to their introduction to Hawai’i, increasing their genetic diversity, which may have increased their adaptive potential. In 1935, 101 cane toads were taken from Honolulu, Hawai’i to Gordonvale, Queensland in northern Australia, marking one of the worst anthropogenic biological control actions in human history (Turvey, 2013). Recent phylogenetic and ecological studies suggest that two ecotypes, rainforest and coastal, were presented in native French Guiana and Guyana populations (DeVore et al., 2021; Mittan‐Moreau et al., 2022). However, historical records lacked the specific localities of where toads were taken from in the native range, thus, it is unknown whether both ecotypes were introduced into Caribbean Islands. Furthermore, toads might have been present in Barbados for years before being translocated to Puerto Rico, providing ample time for admixture between ecotypes to occur.

Figure S1: Residuals of the ln-ln regression of arm length (humerus, radioulna) and length (tibiofibula, femur) with ln snout-vent length (SVL) as covariate between male native cane toads (*Rhinella marina*) sampled in rainforest *versus* coastal environments in French Guiana.

Figure S2: Isolation by Distance (IBD) based on a panel of 58,987 SNPs from 44 native cane toads (*Rhinella marina*) sampled from French Guiana.

Figure S3: Population structure of native and invasive cane toads (*Rhinella marina*). Principal Component Analysis based on a panel of 58,987 SNPs from (A) 44 native cane toads (PC1, PC3 axes) and (B) 46 invasive cane toads (PC1, PC3 axes).

Figure S4: Relationship between snout-vent length (SVL) and the principal component scores from PC2, Figure 3C, which is based on genetic data that separated FG-Northeastern (NE) Rainforest toads from FG-NE coast toads (*Rhinella marina*) (Mantel *r* = -0.114, *p* > 0.05). FG-Northwestern (NW) toads were excluded due to the absence of morphological measurements. Native toads from the rainforest and coast are denoted by triangles and circles, respectively.

Figure S5: Analysis of loci putative under selection between native rainforest and coastal toads (*Rhinella marina*) sampled in French Guiana. Frequency was calculated based on the reference allele in the reference genome of an invasive toad. Only outlier SNPs that are fixed in the invasive range are shown. Heatmap indicates the absolute allelic frequency of the populations, ordered by the difference between the rainforest and coastal populations in the native range.

Figure S6: Principal Component Analysis based on a subset of 46 invasive cane toads (*Rhinella marina*) sampled in Australia, coloured by sample sexes.

Figure S7: Principal Component Analysis based on a panel of 274 SNPs that separate invasive range cane toads (*Rhinella marina*) in each invasive range sampling site (see Figure 2D).

| Table S1: Summary information of cane toads (*Rhinella marina*) used for morphological analysis in this study. | | | | | | | |
| --- | --- | --- | --- | --- | --- | --- | --- |
|  |  |  |  |  |  |  |  |
| **Site** | **Location** | **Environment** | **Sample ID** | **Sex** | **Latitude** | **Longitude** | **Collection dates** |
| **French Guiana** | Lake Rorota | FG-NE Coast | 16-597 | Female | 4.88066384 | -52.2516451 | 2016-07-23 |
|  | Lake Rorota | FG-NE Coast | 16-598 | Female | 4.88066384 | -52.2516451 | 2016-07-23 |
|  | Lake Rorota | FG-NE Coast | 16-599 | Male | 4.88066384 | -52.2516451 | 2016-07-23 |
|  | Lake Rorota | FG-NE Coast | 16-600 | Male | 4.88066384 | -52.2516451 | 2016-07-23 |
|  | Lake Rorota | FG-NE Coast | 16-601 | Female | 4.88066384 | -52.2516451 | 2016-07-23 |
|  | Lake Rorota | FG-NE Coast | 16-602 | Female | 4.88066384 | -52.2516451 | 2016-07-23 |
|  | Lake Rorota | FG-NE Coast | 16-603 | Male | 4.88066384 | -52.2516451 | 2016-07-23 |
|  | Lake Rorota | FG-NE Coast | 16-604 | Male | 4.88066384 | -52.2516451 | 2016-07-23 |
|  | Lake Rorota | FG-NE Coast | 16-605 | Female | 4.88066384 | -52.2516451 | 2016-07-23 |
|  | Lake Rorota | FG-NE Coast | 16-606 | Female | 4.88066384 | -52.2516451 | 2016-07-23 |
|  | Lake Rorota | FG-NE Coast | 16-607 | Female | 4.88066384 | -52.2516451 | 2016-07-23 |
|  | Lake Rorota | FG-NE Coast | 16-608 | Male | 4.88066384 | -52.2516451 | 2016-07-23 |
|  | Lake Rorota | FG-NE Coast | 16-609 | Female | 4.88066384 | -52.2516451 | 2016-07-23 |
|  | Little Rorota Pond | FG-NE Coast | 16-612 | Female | 4.88066384 | -52.2516451 | 2016-07-23 |
|  | Little Rorota Pond | FG-NE Coast | 16-613 | Female | 4.88066384 | -52.2516451 | 2016-07-23 |
|  | Little Rorota Pond | FG-NE Coast | 16-614 | Female | 4.88066384 | -52.2516451 | 2016-07-23 |
|  | Little Rorota Pond | FG-NE Coast | 16-616 | Female | 4.88066384 | -52.2516451 | 2016-07-23 |
|  | Rorota Parking Lot | FG-NE Coast | 16-617 | Female | 4.88066384 | -52.2516451 | 2016-07-23 |
|  | South Remire-Montjoly Beach | FG-NE Coast | 16-624 | Male | 4.8900923 | -52.2539511 | 2016-07-23 |
|  | South Remire-Montjoly Beach | FG-NE Coast | 16-625 | Male | 4.8900923 | -52.2539511 | 2016-07-23 |
|  | South Remire-Montjoly Beach | FG-NE Coast | 16-626 | Male | 4.8900923 | -52.2539511 | 2016-07-23 |
|  | South Remire-Montjoly Beach | FG-NE Coast | 16-627 | Male | 4.8900923 | -52.2539511 | 2016-07-23 |
|  | South Remire-Montjoly Beach | FG-NE Coast | 16-628 | Male | 4.8900923 | -52.2539511 | 2016-07-23 |
|  | South Remire-Montjoly Beach | FG-NE Coast | 16-629 | Male | 4.8900923 | -52.2539511 | 2016-07-23 |
|  | South Remire-Montjoly Beach | FG-NE Coast | 16-630 | Female | 4.8900923 | -52.2539511 | 2016-07-23 |
|  | South Remire-Montjoly Beach | FG-NE Coast | 16-631 | Female | 4.8900923 | -52.2539511 | 2016-07-23 |
|  | South Remire-Montjoly Beach | FG-NE Coast | 16-632 | Male | 4.8900923 | -52.2539511 | 2016-07-23 |
|  | South Remire-Montjoly Beach | FG-NE Coast | 16-634 | Male | 4.8900923 | -52.2539511 | 2016-07-23 |
|  | South Remire-Montjoly Beach | FG-NE Coast | 16-635 | Male | 4.8900923 | -52.2539511 | 2016-07-23 |
|  | South Remire-Montjoly Beach | FG-NE Coast | 16-636 | Male | 4.8900923 | -52.2539511 | 2016-07-23 |
|  | South Remire-Montjoly Beach | FG-NE Coast | 16-638 | Male | 4.8900923 | -52.2539511 | 2016-07-23 |
|  | South Remire-Montjoly Beach | FG-NE Coast | 16-639 | Male | 4.8900923 | -52.2539511 | 2016-07-23 |
|  | South Remire-Montjoly Beach | FG-NE Coast | 16-640 | Male | 4.8900923 | -52.2539511 | 2016-07-23 |
|  | South Remire-Montjoly Beach | FG-NE Coast | 16-641 | Male | 4.8900923 | -52.2539511 | 2016-07-23 |
|  | South Remire-Montjoly Beach | FG-NE Coast | 16-642 | Male | 4.8900923 | -52.2539511 | 2016-07-23 |
|  | South Remire-Montjoly Beach | FG-NE Coast | 16-643 | Male | 4.8900923 | -52.2539511 | 2016-07-23 |
|  | South Remire-Montjoly Beach | FG-NE Coast | 16-644 | Male | 4.8900923 | -52.2539511 | 2016-07-23 |
|  | South Remire-Montjoly Beach | FG-NE Coast | 16-645 | Male | 4.8900923 | -52.2539511 | 2016-07-23 |
|  | South Remire-Montjoly Beach | FG-NE Coast | 16-646 | Male | 4.8900923 | -52.2539511 | 2016-07-23 |
|  | South Remire-Montjoly Beach | FG-NE Coast | 16-648 | Male | 4.8900923 | -52.2539511 | 2016-07-23 |
|  | South Remire-Montjoly Beach | FG-NE Coast | 16-649 | Male | 4.8900923 | -52.2539511 | 2016-07-23 |
|  | South Remire-Montjoly Beach | FG-NE Coast | 16-650 | Male | 4.8900923 | -52.2539511 | 2016-07-23 |
|  | South Remire-Montjoly Beach | FG-NE Coast | 16-651 | Male | 4.8900923 | -52.2539511 | 2016-07-23 |
|  | South Remire-Montjoly Beach | FG-NE Coast | 16-652 | Female | 4.8900923 | -52.2539511 | 2016-07-23 |
|  | South Remire-Montjoly Beach | FG-NE Coast | 16-653 | Male | 4.8900923 | -52.2539511 | 2016-07-23 |
|  | South Remire-Montjoly Beach | FG-NE Coast | 16-654 | Male | 4.8900923 | -52.2539511 | 2016-07-23 |
|  | South Remire-Montjoly Beach | FG-NE Coast | 16-655 | Male | 4.8900923 | -52.2539511 | 2016-07-23 |
|  | South Remire-Montjoly Beach | FG-NE Coast | 16-656 | Male | 4.8900923 | -52.2539511 | 2016-07-23 |
|  | South Remire-Montjoly Beach | FG-NE Coast | 16-657 | Male | 4.8900923 | -52.2539511 | 2016-07-23 |
|  | South Remire-Montjoly Beach | FG-NE Coast | 16-658 | Male | 4.8900923 | -52.2539511 | 2016-07-23 |
|  | Family Plaza | FG-NE Coast | 16-659 | Female | 4.89135369 | -52.3338313 | 2016-07-24 |
|  | Family Plaza | FG-NE Coast | 16-660 | Male | 4.89135369 | -52.3338313 | 2016-07-24 |
|  | Family Plaza | FG-NE Coast | 16-661 | Male | 4.89135369 | -52.3338313 | 2016-07-24 |
|  | Family Plaza | FG-NE Coast | 16-662 | Male | 4.89135369 | -52.3338313 | 2016-07-24 |
|  | Family Plaza | FG-NE Coast | 16-663 | Female | 4.89135369 | -52.3338313 | 2016-07-24 |
|  | Family Plaza | FG-NE Coast | 16-664 | Female | 4.89135369 | -52.3338313 | 2016-07-24 |
|  | Family Plaza | FG-NE Coast | 16-665 | Male | 4.89135369 | -52.3338313 | 2016-07-24 |
|  | Family Plaza | FG-NE Coast | 16-667 | Female | 4.89135369 | -52.3338313 | 2016-07-24 |
|  | Family Plaza | FG-NE Coast | 16-668 | Male | 4.89135369 | -52.3338313 | 2016-07-24 |
|  | Family Plaza | FG-NE Coast | 16-670 | Female | 4.89135369 | -52.3338313 | 2016-07-24 |
|  | Family Plaza | FG-NE Coast | 16-671 | Male | 4.89135369 | -52.3338313 | 2016-07-24 |
|  | Family Plaza | FG-NE Coast | 16-675 | Male | 4.89135369 | -52.3338313 | 2016-07-24 |
|  | Family Plaza | FG-NE Coast | 16-676 | Male | 4.89135369 | -52.3338313 | 2016-07-24 |
|  | Family Plaza | FG-NE Coast | 16-678 | Male | 4.89135369 | -52.3338313 | 2016-07-24 |
|  | Family Plaza | FG-NE Coast | 16-683 | Female | 4.89135369 | -52.3338313 | 2016-07-24 |
|  | Family Plaza | FG-NE Coast | 16-684 | Male | 4.89135369 | -52.3338313 | 2016-07-24 |
|  | Family Plaza | FG-NE Coast | 16-686 | Female | 4.89135369 | -52.3338313 | 2016-07-24 |
|  | South Remire-Montjoly Beach | FG-NE Coast | 16-691 | Female | 4.8900923 | -52.2539511 | 2016-07-25 |
|  | South Remire-Montjoly Beach | FG-NE Coast | 16-692 | Male | 4.8900923 | -52.2539511 | 2016-07-25 |
|  | South Remire-Montjoly Beach | FG-NE Coast | 16-694 | Male | 4.8900923 | -52.2539511 | 2016-07-25 |
|  | South Remire-Montjoly Beach | FG-NE Coast | 16-695 | Female | 4.8900923 | -52.2539511 | 2016-07-25 |
|  | South Remire-Montjoly Beach | FG-NE Coast | 16-696 | Female | 4.8900923 | -52.2539511 | 2016-07-25 |
|  | South Remire-Montjoly Beach | FG-NE Coast | 16-697 | Male | 4.8900923 | -52.2539511 | 2016-07-25 |
|  | South Remire-Montjoly Beach | FG-NE Coast | 16-698 | Male | 4.8900923 | -52.2539511 | 2016-07-25 |
|  | South Remire-Montjoly Beach | FG-NE Coast | 16-699 | Male | 4.8900923 | -52.2539511 | 2016-07-25 |
|  | South Remire-Montjoly Beach | FG-NE Coast | 16-701 | Male | 4.8900923 | -52.2539511 | 2016-07-25 |
|  | South Remire-Montjoly Beach | FG-NE Coast | 16-702 | Male | 4.8900923 | -52.2539511 | 2016-07-25 |
|  | South Remire-Montjoly Beach | FG-NE Coast | 16-703 | Female | 4.8900923 | -52.2539511 | 2016-07-25 |
|  | South Remire-Montjoly Beach | FG-NE Coast | 16-704 | Female | 4.8900923 | -52.2539511 | 2016-07-25 |
|  | South Remire-Montjoly Beach | FG-NE Coast | 16-705 | Male | 4.8900923 | -52.2539511 | 2016-07-25 |
|  | South Remire-Montjoly Beach | FG-NE Coast | 16-706 | Male | 4.8900923 | -52.2539511 | 2016-07-25 |
|  | South Remire-Montjoly Beach | FG-NE Coast | 16-707 | Male | 4.8900923 | -52.2539511 | 2016-07-25 |
|  | South Remire-Montjoly Beach | FG-NE Coast | 16-708 | Male | 4.8900923 | -52.2539511 | 2016-07-25 |
|  | South Remire-Montjoly Beach | FG-NE Coast | 16-709 | Female | 4.8900923 | -52.2539511 | 2016-07-25 |
|  | Kaw Marshes | FG-NE Rainforest | 16-711 | Male | 4.5020833 | -52.057 | 2016-07-26 |
|  | Kaw Marshes | FG-NE Rainforest | 16-712 | Male | 4.5180278 | -52.0977778 | 2016-07-26 |
|  | Kaw Marshes | FG-NE Rainforest | 16-714 | Male | 4.5333333 | -52.1281389 | 2016-07-26 |
|  | Kaw Marshes | FG-NE Rainforest | 16-715 | Male | 4.5333333 | -52.1281389 | 2016-07-26 |
|  | Kaw Marshes | FG-NE Rainforest | 16-716 | Male | 4.5489722 | -52.1719444 | 2016-07-26 |
|  | Kaw Marshes | FG-NE Rainforest | 16-717 | Male | 4.5489722 | -52.1719444 | 2016-07-26 |
|  | Kaw Marshes | FG-NE Rainforest | 16-723 | Female | 4.5956111 | -52.2625556 | 2016-07-26 |
|  | Regina Marshes | FG-NE Rainforest | 16-730 | Male | 4.3625833 | -52.2786389 | 2016-07-27 |
|  | Regina Marshes | FG-NE Rainforest | 16-731 | Male | 4.3625833 | -52.2786389 | 2016-07-27 |
|  | Regina Marshes | FG-NE Rainforest | 16-732 | Male | 4.3625833 | -52.2786389 | 2016-07-27 |
|  | Regina Marshes | FG-NE Rainforest | 16-733 | Male | 4.3625833 | -52.2786389 | 2016-07-27 |
|  | Regina Marshes | FG-NE Rainforest | 16-735 | Male | 4.3625833 | -52.2786389 | 2016-07-27 |
|  | Regina Marshes | FG-NE Rainforest | 16-736 | Male | 4.3625833 | -52.2786389 | 2016-07-27 |
|  | Regina Marshes | FG-NE Rainforest | 16-737 | Male | 4.3625833 | -52.2786389 | 2016-07-27 |
|  | Regina Marshes | FG-NE Rainforest | 16-738 | Male | 4.3625833 | -52.2786389 | 2016-07-27 |
|  | Regina Marshes | FG-NE Rainforest | 16-739 | Male | 4.3625833 | -52.2786389 | 2016-07-27 |
|  | St. Georges Shoreline | FG-NE Rainforest | 16-740 | Female | 3.89531834 | -51.7981835 | 2016-07-28 |
|  | St. Georges Shoreline | FG-NE Rainforest | 16-741 | Female | 3.89531834 | -51.7981835 | 2016-07-28 |
|  | St. Georges Aerodrome | FG-NE Rainforest | 16-742 | Male | 3.90045627 | -51.8015309 | 2016-07-28 |
|  | St. Georges Aerodrome | FG-NE Rainforest | 16-743 | Male | 3.90045627 | -51.8015309 | 2016-07-28 |
|  | St. Georges Aerodrome | FG-NE Rainforest | 16-744 | Male | 3.90045627 | -51.8015309 | 2016-07-28 |
|  | St. Georges Aerodrome | FG-NE Rainforest | 16-745 | Male | 3.90045627 | -51.8015309 | 2016-07-28 |
|  | St. Georges Aerodrome | FG-NE Rainforest | 16-746 | Male | 3.90045627 | -51.8015309 | 2016-07-28 |
|  | St. Georges Aerodrome | FG-NE Rainforest | 16-747 | Male | 3.90045627 | -51.8015309 | 2016-07-28 |
|  | St. Georges Aerodrome | FG-NE Rainforest | 16-748 | Female | 3.90045627 | -51.8015309 | 2016-07-28 |
|  | St. Georges Aerodrome | FG-NE Rainforest | 16-749 | Female | 3.90045627 | -51.8015309 | 2016-07-28 |
|  | St. Georges Aerodrome | FG-NE Rainforest | 16-750 | Male | 3.90045627 | -51.8015309 | 2016-07-28 |
|  | St. Georges Aerodrome | FG-NE Rainforest | 16-753 | Male | 3.90045627 | -51.8015309 | 2016-07-28 |
|  | St. Georges Aerodrome | FG-NE Rainforest | 16-754 | Male | 3.90045627 | -51.8015309 | 2016-07-28 |
|  | St. Georges Aerodrome | FG-NE Rainforest | 16-756 | Male | 3.90045627 | -51.8015309 | 2016-07-28 |
|  | St. Georges Aerodrome | FG-NE Rainforest | 16-758 | Male | 3.90045627 | -51.8015309 | 2016-07-28 |
|  | St. Georges Aerodrome | FG-NE Rainforest | 16-760 | Male | 3.90045627 | -51.8015309 | 2016-07-28 |
|  | St. Georges Aerodrome | FG-NE Rainforest | 16-761 | Male | 3.90045627 | -51.8015309 | 2016-07-28 |
|  | St. Georges Aerodrome | FG-NE Rainforest | 16-762 | Male | 3.90045627 | -51.8015309 | 2016-07-28 |
|  | St. Georges Aerodrome | FG-NE Rainforest | 16-765 | Female | 3.90045627 | -51.8015309 | 2016-07-28 |
|  | St. Georges Lumber Yard | FG-NE Rainforest | 16-769 | Male | 3.9460278 | -51.8504444 | 2016-07-28 |
|  | St. Georges Lumber Yard | FG-NE Rainforest | 16-770 | Male | 3.9460278 | -51.8504444 | 2016-07-28 |
|  | St. Georges Lumber Yard | FG-NE Rainforest | 16-771 | Male | 3.9460278 | -51.8504444 | 2016-07-28 |
|  | St. Georges Lumber Yard | FG-NE Rainforest | 16-772 | Male | 3.9460278 | -51.8504444 | 2016-07-28 |
|  | St. Georges Lumber Yard | FG-NE Rainforest | 16-773 | Male | 3.9460278 | -51.8504444 | 2016-07-28 |
|  | St. Georges Lumber Yard | FG-NE Rainforest | 16-774 | Male | 3.9460278 | -51.8504444 | 2016-07-28 |
|  | St. Georges Lumber Yard | FG-NE Rainforest | 16-775 | Male | 3.9460278 | -51.8504444 | 2016-07-28 |
|  | St. Georges Lumber Yard | FG-NE Rainforest | 16-776 | Male | 3.9460278 | -51.8504444 | 2016-07-28 |
|  | St. Georges Lumber Yard | FG-NE Rainforest | 16-777 | Male | 3.9460278 | -51.8504444 | 2016-07-28 |
|  | St. Georges Lumber Yard | FG-NE Rainforest | 16-778 | Male | 3.9460278 | -51.8504444 | 2016-07-28 |
|  | St. Georges Lumber Yard | FG-NE Rainforest | 16-779 | Male | 3.9460278 | -51.8504444 | 2016-07-28 |
|  | North Remire-Montjoly Beach | FG-NE Coast | 16-795 | Female | 4.91707277 | -52.2669545 | 2016-07-29 |
|  | North Remire-Montjoly Beach | FG-NE Coast | 16-796 | Female | 4.91707277 | -52.2669545 | 2016-07-29 |
|  | Kourou Atlantis | FG-NE Coast | 16-804 | Female | 5.15715438 | -52.6464053 | 2016-07-29 |
|  | Kourou Atlantis | FG-NE Coast | 16-805 | Female | 5.15715438 | -52.6464053 | 2016-07-29 |
|  | Kourou Atlantis | FG-NE Coast | 16-806 | Male | 5.15715438 | -52.6464053 | 2016-07-29 |
|  | Kourou Atlantis | FG-NE Coast | 16-807 | Female | 5.15715438 | -52.6464053 | 2016-07-29 |
|  | Kourou Atlantis | FG-NE Coast | 16-808 | Male | 5.15715438 | -52.6464053 | 2016-07-29 |
|  | Kourou Atlantis | FG-NE Coast | 16-809 | Female | 5.15715438 | -52.6464053 | 2016-07-29 |
|  | Kourou Atlantis | FG-NE Coast | 16-810 | Female | 5.15715438 | -52.6464053 | 2016-07-29 |
|  | Kourou Atlantis | FG-NE Coast | 16-811 | Female | 5.15715438 | -52.6464053 | 2016-07-29 |
|  | Kourou Hotel Des Roches | FG-NE Coast | 16-812 | Male | 5.160194 | -52.6274025 | 2016-07-29 |
|  | Kourou Hotel Des Roches | FG-NE Coast | 16-824 | Male | 5.160194 | -52.6274025 | 2016-07-29 |
|  | Kourou Hotel Des Roches | FG-NE Coast | 16-825 | Male | 5.160194 | -52.6274025 | 2016-07-29 |
|  | Kourou Hotel Des Roches | FG-NE Coast | 16-826 | Male | 5.160194 | -52.6274025 | 2016-07-29 |
|  | Kourou Hotel Des Roches | FG-NE Coast | 16-827 | Male | 5.160194 | -52.6274025 | 2016-07-29 |
|  | Kourou Hotel Des Roches | FG-NE Coast | 16-828 | Male | 5.160194 | -52.6274025 | 2016-07-29 |
|  | Kourou Hotel Des Roches | FG-NE Coast | 16-829 | Male | 5.160194 | -52.6274025 | 2016-07-29 |
|  | Kourou Hotel Des Roches | FG-NE Coast | 16-830 | Male | 5.160194 | -52.6274025 | 2016-07-29 |
|  | Kourou Hotel Des Roches | FG-NE Coast | 16-831 | Male | 5.160194 | -52.6274025 | 2016-07-29 |
|  | Kourou Hotel Des Roches | FG-NE Coast | 16-832 | Male | 5.160194 | -52.6274025 | 2016-07-29 |
|  | Kourou Hotel Des Roches | FG-NE Coast | 16-834 | Male | 5.160194 | -52.6274025 | 2016-07-29 |
|  | Kourou Hotel Des Roches | FG-NE Coast | 16-835 | Male | 5.160194 | -52.6274025 | 2016-07-29 |
|  | Kourou Hotel Des Roches | FG-NE Coast | 16-1067 | Male | 5.160194 | -52.6274025 | 2016-07-29 |
|  | Kourou Hotel Des Roches | FG-NE Coast | 16-836 | Male | 5.160194 | -52.6274025 | 2016-07-29 |
|  | Kourou Hotel Des Roches | FG-NE Coast | 16-837 | Male | 5.160194 | -52.6274025 | 2016-07-29 |
|  | Kourou Hotel Des Roches | FG-NE Coast | 16-838 | Male | 5.160194 | -52.6274025 | 2016-07-29 |
|  | Kourou Hotel Des Roches | FG-NE Coast | 16-839 | Male | 5.160194 | -52.6274025 | 2016-07-29 |
|  | Kourou Hotel Des Roches | FG-NE Coast | 16-840 | Male | 5.160194 | -52.6274025 | 2016-07-29 |
|  | Kourou Hotel Des Roches | FG-NE Coast | 16-841 | Male | 5.160194 | -52.6274025 | 2016-07-29 |
|  | Kourou Hotel Des Roches | FG-NE Coast | 16-844 | Male | 5.160194 | -52.6274025 | 2016-07-29 |
|  | Kourou Hotel Des Roches | FG-NE Coast | 16-846 | Male | 5.160194 | -52.6274025 | 2016-07-29 |
|  | Kourou Hotel Des Roches | FG-NE Coast | 16-848 | Male | 5.160194 | -52.6274025 | 2016-07-29 |
|  | Kourou Hotel Des Roches | FG-NE Coast | 16-849 | Male | 5.160194 | -52.6274025 | 2016-07-29 |
|  | Kourou Hotel Des Roches | FG-NE Coast | 16-850 | Male | 5.160194 | -52.6274025 | 2016-07-29 |
|  | Kourou Hotel Des Roches | FG-NE Coast | 16-851 | Female | 5.160194 | -52.6274025 | 2016-07-29 |
|  | Kourou Hotel Des Roches | FG-NE Coast | 16-855 | Male | 5.160194 | -52.6274025 | 2016-07-29 |
|  | Kourou Hotel Des Roches | FG-NE Coast | 16-856 | Female | 5.160194 | -52.6274025 | 2016-07-29 |
|  | Kourou Hotel Des Roches | FG-NE Coast | 16-857 | Male | 5.160194 | -52.6274025 | 2016-07-29 |
|  | Kourou Hotel Des Roches | FG-NE Coast | 16-858 | Male | 5.160194 | -52.6274025 | 2016-07-29 |
|  | Kourou Hotel Des Roches | FG-NE Coast | 16-861 | Female | 5.160194 | -52.6274025 | 2016-07-29 |
|  | Kourou Hotel Des Roches | FG-NE Coast | 16-862 | Male | 5.160194 | -52.6274025 | 2016-07-29 |
|  | Kourou Hotel Des Roches | FG-NE Coast | 16-863 | Female | 5.160194 | -52.6274025 | 2016-07-29 |
|  | Kourou Hotel Des Roches | FG-NE Coast | 16-864 | Female | 5.160194 | -52.6274025 | 2016-07-29 |
|  | Kourou Hotel Des Roches | FG-NE Coast | 16-865 | Female | 5.160194 | -52.6274025 | 2016-07-29 |
|  | Kourou Hotel Des Roches | FG-NE Coast | 16-866 | Male | 5.160194 | -52.6274025 | 2016-07-29 |
|  | Kourou Hotel Des Roches | FG-NE Coast | 16-867 | Male | 5.160194 | -52.6274025 | 2016-07-29 |
|  | Kourou Hotel Des Roches | FG-NE Coast | 16-868 | Male | 5.160194 | -52.6274025 | 2016-07-29 |
|  | Kourou Hotel Des Roches | FG-NE Coast | 16-869 | Male | 5.160194 | -52.6274025 | 2016-07-29 |
|  | Kourou Hotel Des Roches | FG-NE Coast | 16-870 | Male | 5.160194 | -52.6274025 | 2016-07-29 |
|  | Kourou Hotel Des Roches | FG-NE Coast | 16-871 | Male | 5.160194 | -52.6274025 | 2016-07-29 |
|  | Kourou Hotel Des Roches | FG-NE Coast | 16-872 | Male | 5.160194 | -52.6274025 | 2016-07-29 |
|  | Kourou Hotel Des Roches | FG-NE Coast | 16-873 | Male | 5.160194 | -52.6274025 | 2016-07-29 |
|  | Kourou Hotel Des Roches | FG-NE Coast | 16-874 | Male | 5.160194 | -52.6274025 | 2016-07-29 |
|  | Maison de Nature | FG-NE Coast | 16-907 | Male | 5.41226972 | -53.0324355 | 2016-08-01 |
|  | Maison de Nature | FG-NE Coast | 16-908 | Male | 5.41226972 | -53.0324355 | 2016-08-01 |
|  | Maison de Nature | FG-NE Coast | 16-909 | Male | 5.41226972 | -53.0324355 | 2016-08-01 |
|  | Maison de Nature | FG-NE Coast | 16-910 | Male | 5.41226972 | -53.0324355 | 2016-08-01 |
|  | Sinamary Pond | FG-NE Coast | 16-912 | Male | 5.27558027 | -52.9274297 | 2016-08-01 |
|  | Sinamary Pond | FG-NE Coast | 16-913 | Male | 5.27558027 | -52.9274297 | 2016-08-01 |
|  | Sinamary Pond | FG-NE Coast | 16-914 | Male | 5.27558027 | -52.9274297 | 2016-08-01 |
|  | Sinamary Pond | FG-NE Coast | 16-915 | Male | 5.27558027 | -52.9274297 | 2016-08-01 |
|  | North Remire-Montjoly Beach | FG-NE Coast | 16-916 | Female | 4.91707277 | -52.2669545 | 2016-08-03 |
|  | Yalimapo Beach | FG-NW Coast | 16-917 | Male | 5.74632008 | -53.9417258 | 2016-08-04 |
|  | Yalimapo Beach | FG-NW Coast | 16-918 | Female | 5.74632008 | -53.9417258 | 2016-08-04 |
|  | Yalimapo Beach | FG-NW Coast | 16-919 | Male | 5.74632008 | -53.9417258 | 2016-08-04 |
|  | Yalimapo Beach | FG-NW Coast | 16-920 | Female | 5.74632008 | -53.9417258 | 2016-08-04 |
|  | Yalimapo Beach | FG-NW Coast | 16-921 | Female | 5.74632008 | -53.9417258 | 2016-08-04 |
|  | Yalimapo Beach | FG-NW Coast | 16-922 | Female | 5.74632008 | -53.9417258 | 2016-08-04 |
|  | Yalimapo Beach | FG-NW Coast | 16-923 | Female | 5.74632008 | -53.9417258 | 2016-08-04 |
|  | Yalimapo Beach | FG-NW Coast | 16-924 | Female | 5.74632008 | -53.9417258 | 2016-08-04 |
|  | Yalimapo Beach | FG-NW Coast | 16-925 | Male | 5.74632008 | -53.9417258 | 2016-08-04 |
|  | Yalimapo Municipal Building | FG-NW Coast | 16-926 | Female | 5.73611477 | -53.9112559 | 2016-08-04 |
|  | North Remire-Montjoly Beach | FG-NE Coast | 16-951 | Male | 4.91707277 | -52.2669545 | 2016-08-05 |
|  | North Remire-Montjoly Beach | FG-NE Coast | 16-952 | Male | 4.91707277 | -52.2669545 | 2016-08-05 |
|  | North Remire-Montjoly Beach | FG-NE Coast | 16-953 | Male | 4.91707277 | -52.2669545 | 2016-08-05 |
|  | North Remire-Montjoly Beach | FG-NE Coast | 16-954 | Male | 4.91707277 | -52.2669545 | 2016-08-05 |
|  | North Remire-Montjoly Beach | FG-NE Coast | 16-956 | Male | 4.91707277 | -52.2669545 | 2016-08-05 |
|  | North Remire-Montjoly Beach | FG-NE Coast | 16-957 | Male | 4.91707277 | -52.2669545 | 2016-08-05 |
|  | North Remire-Montjoly Beach | FG-NE Coast | 16-958 | Male | 4.91707277 | -52.2669545 | 2016-08-05 |
|  | North Remire-Montjoly Beach | FG-NE Coast | 16-959 | Male | 4.91707277 | -52.2669545 | 2016-08-05 |
|  | North Remire-Montjoly Beach | FG-NE Coast | 16-963 | Male | 4.91707277 | -52.2669545 | 2016-08-05 |
|  | North Remire-Montjoly Beach | FG-NE Coast | 16-964 | Male | 4.91707277 | -52.2669545 | 2016-08-05 |
|  | North Remire-Montjoly Beach | FG-NE Coast | 16-965 | Male | 4.91707277 | -52.2669545 | 2016-08-05 |
|  | North Remire-Montjoly Beach | FG-NE Coast | 16-966 | Male | 4.91707277 | -52.2669545 | 2016-08-05 |
|  | North Remire-Montjoly Beach | FG-NE Coast | 16-967 | Female | 4.91707277 | -52.2669545 | 2016-08-05 |
|  | North Remire-Montjoly Beach | FG-NE Coast | 16-968 | Male | 4.91707277 | -52.2669545 | 2016-08-05 |
|  | North Remire-Montjoly Beach | FG-NE Coast | 16-969 | Female | 4.91707277 | -52.2669545 | 2016-08-05 |
|  | North Remire-Montjoly Beach | FG-NE Coast | 16-970 | Male | 4.91707277 | -52.2669545 | 2016-08-05 |
|  | North Remire-Montjoly Beach | FG-NE Coast | 16-971 | Female | 4.91707277 | -52.2669545 | 2016-08-05 |
|  | North Remire-Montjoly Beach | FG-NE Coast | 16-972 | Male | 4.91707277 | -52.2669545 | 2016-08-05 |
|  | North Remire-Montjoly Beach | FG-NE Coast | 16-973 | Female | 4.91707277 | -52.2669545 | 2016-08-05 |
|  | North Remire-Montjoly Beach | FG-NE Coast | 16-974 | Male | 4.91707277 | -52.2669545 | 2016-08-05 |
|  | North Remire-Montjoly Beach | FG-NE Coast | 16-975 | Female | 4.91707277 | -52.2669545 | 2016-08-05 |
|  | North Remire-Montjoly Beach | FG-NE Coast | 16-976 | Male | 4.91707277 | -52.2669545 | 2016-08-05 |
|  | North Remire-Montjoly Beach | FG-NE Coast | 16-977 | Female | 4.91707277 | -52.2669545 | 2016-08-05 |
|  | North Remire-Montjoly Beach | FG-NE Coast | 16-978 | Male | 4.91707277 | -52.2669545 | 2016-08-05 |
|  | North Remire-Montjoly Beach | FG-NE Coast | 16-979 | Male | 4.91707277 | -52.2669545 | 2016-08-05 |
|  | North Remire-Montjoly Beach | FG-NE Coast | 16-980 | Male | 4.91707277 | -52.2669545 | 2016-08-05 |
|  | North Remire-Montjoly Beach | FG-NE Coast | 16-981 | Male | 4.91707277 | -52.2669545 | 2016-08-05 |
|  | North Remire-Montjoly Beach | FG-NE Coast | 16-982 | Male | 4.91707277 | -52.2669545 | 2016-08-05 |
|  | North Remire-Montjoly Beach | FG-NE Coast | 16-983 | Male | 4.91707277 | -52.2669545 | 2016-08-05 |
|  | North Remire-Montjoly Beach | FG-NE Coast | 16-984 | Male | 4.91707277 | -52.2669545 | 2016-08-05 |
|  | North Remire-Montjoly Beach | FG-NE Coast | 16-985 | Male | 4.91707277 | -52.2669545 | 2016-08-05 |
|  | North Remire-Montjoly Beach | FG-NE Coast | 16-986 | Male | 4.91707277 | -52.2669545 | 2016-08-05 |
|  | North Remire-Montjoly Beach | FG-NE Coast | 16-987 | Male | 4.91707277 | -52.2669545 | 2016-08-05 |
|  | North Remire-Montjoly Beach | FG-NE Coast | 16-988 | Male | 4.91707277 | -52.2669545 | 2016-08-05 |
|  | North Remire-Montjoly Beach | FG-NE Coast | 16-989 | Male | 4.91707277 | -52.2669545 | 2016-08-05 |
|  | North Remire-Montjoly Beach | FG-NE Coast | 16-991 | Male | 4.91707277 | -52.2669545 | 2016-08-05 |
|  | North Remire-Montjoly Beach | FG-NE Coast | 16-992 | Male | 4.91707277 | -52.2669545 | 2016-08-05 |
|  | North Remire-Montjoly Beach | FG-NE Coast | 16-993 | Male | 4.91707277 | -52.2669545 | 2016-08-05 |
|  | North Remire-Montjoly Beach | FG-NE Coast | 16-994 | Male | 4.91707277 | -52.2669545 | 2016-08-05 |
|  | North Remire-Montjoly Beach | FG-NE Coast | 16-995 | Male | 4.91707277 | -52.2669545 | 2016-08-05 |
|  | North Remire-Montjoly Beach | FG-NE Coast | 16-996 | Male | 4.91707277 | -52.2669545 | 2016-08-05 |
|  | North Remire-Montjoly Beach | FG-NE Coast | 16-998 | Male | 4.91707277 | -52.2669545 | 2016-08-05 |
|  | CNRS House | FG-NE Coast | 16-1001 | Female | 4.87771341 | -52.3335309 | 2016-08-05 |
|  | CNRS House | FG-NE Coast | 16-1002 | Male | 4.87771341 | -52.3335309 | 2016-08-05 |
|  | CNRS House | FG-NE Coast | 16-1003 | Male | 4.87771341 | -52.3335309 | 2016-08-05 |
|  | CNRS House | FG-NE Coast | 16-1004 | Male | 4.87771341 | -52.3335309 | 2016-08-05 |
|  | CNRS House | FG-NE Coast | 16-1005 | Male | 4.87771341 | -52.3335309 | 2016-08-05 |
|  | CNRS House | FG-NE Coast | 16-1006 | Male | 4.87771341 | -52.3335309 | 2016-08-05 |
|  | Family Plaza | FG-NE Coast | 16-1007 | Male | 4.89135369 | -52.3338313 | 2016-08-07 |
|  | Family Plaza | FG-NE Coast | 16-1008 | Female | 4.89135369 | -52.3338313 | 2016-08-07 |
|  | Family Plaza | FG-NE Coast | 16-1009 | Male | 4.89135369 | -52.3338313 | 2016-08-07 |
|  | Family Plaza | FG-NE Coast | 16-1010 | Female | 4.89135369 | -52.3338313 | 2016-08-07 |
|  | Family Plaza | FG-NE Coast | 16-1011 | Male | 4.89135369 | -52.3338313 | 2016-08-07 |
|  | Family Plaza | FG-NE Coast | 16-1012 | Female | 4.89135369 | -52.3338313 | 2016-08-07 |
|  | Family Plaza | FG-NE Coast | 16-1013 | Male | 4.89135369 | -52.3338313 | 2016-08-07 |
|  | Family Plaza | FG-NE Coast | 16-1014 | Female | 4.89135369 | -52.3338313 | 2016-08-07 |
|  | Family Plaza | FG-NE Coast | 16-1015 | Male | 4.89135369 | -52.3338313 | 2016-08-07 |
|  | Family Plaza | FG-NE Coast | 16-1016 | Female | 4.89135369 | -52.3338313 | 2016-08-07 |
|  | Family Plaza | FG-NE Coast | 16-1017 | Male | 4.89135369 | -52.3338313 | 2016-08-07 |
|  | Family Plaza | FG-NE Coast | 16-1018 | Female | 4.89135369 | -52.3338313 | 2016-08-07 |
|  | Family Plaza | FG-NE Coast | 16-1019 | Male | 4.89135369 | -52.3338313 | 2016-08-07 |
|  | Family Plaza | FG-NE Coast | 16-1020 | Male | 4.89135369 | -52.3338313 | 2016-08-07 |
|  | Family Plaza | FG-NE Coast | 16-1021 | Male | 4.89135369 | -52.3338313 | 2016-08-07 |
|  | Family Plaza | FG-NE Coast | 16-1022 | Female | 4.89135369 | -52.3338313 | 2016-08-07 |
|  | Family Plaza | FG-NE Coast | 16-1023 | Female | 4.89135369 | -52.3338313 | 2016-08-07 |
|  | Family Plaza | FG-NE Coast | 16-1024 | Male | 4.89135369 | -52.3338313 | 2016-08-07 |
|  | South Remire-Montjoly Beach | FG-NE Coast | 16-1037 | Male | 4.8900923 | -52.2539511 | 2016-08-08 |
|  | South Remire-Montjoly Beach | FG-NE Coast | 16-1038 | Female | 4.8900923 | -52.2539511 | 2016-08-08 |
|  | South Remire-Montjoly Beach | FG-NE Coast | 16-1039 | Male | 4.8900923 | -52.2539511 | 2016-08-08 |
|  | South Remire-Montjoly Beach | FG-NE Coast | 16-1040 | Female | 4.8900923 | -52.2539511 | 2016-08-08 |
|  | South Remire-Montjoly Beach | FG-NE Coast | 16-1041 | Male | 4.8900923 | -52.2539511 | 2016-08-08 |
|  | South Remire-Montjoly Beach | FG-NE Coast | 16-1042 | Female | 4.8900923 | -52.2539511 | 2016-08-08 |
|  | South Remire-Montjoly Beach | FG-NE Coast | 16-1043 | Male | 4.8900923 | -52.2539511 | 2016-08-08 |
|  | South Remire-Montjoly Beach | FG-NE Coast | 16-1044 | Male | 4.8900923 | -52.2539511 | 2016-08-08 |
|  | South Remire-Montjoly Beach | FG-NE Coast | 16-1045 | Female | 4.8900923 | -52.2539511 | 2016-08-08 |
|  | South Remire-Montjoly Beach | FG-NE Coast | 16-1046 | Female | 4.8900923 | -52.2539511 | 2016-08-08 |
|  | South Remire-Montjoly Beach | FG-NE Coast | 16-1047 | Male | 4.8900923 | -52.2539511 | 2016-08-08 |
|  | South Remire-Montjoly Beach | FG-NE Coast | 16-1048 | Female | 4.8900923 | -52.2539511 | 2016-08-08 |
|  | South Remire-Montjoly Beach | FG-NE Coast | 16-1049 | Female | 4.8900923 | -52.2539511 | 2016-08-08 |
|  | South Remire-Montjoly Beach | FG-NE Coast | 16-1050 | Male | 4.8900923 | -52.2539511 | 2016-08-08 |
|  | South Remire-Montjoly Beach | FG-NE Coast | 16-1051 | Male | 4.8900923 | -52.2539511 | 2016-08-08 |
|  | South Remire-Montjoly Beach | FG-NE Coast | 16-1052 | Male | 4.8900923 | -52.2539511 | 2016-08-08 |
|  | South Remire-Montjoly Beach | FG-NE Coast | 16-1054 | Female | 4.8900923 | -52.2539511 | 2016-08-08 |
|  | South Remire-Montjoly Beach | FG-NE Coast | 16-1055 | Male | 4.8900923 | -52.2539511 | 2016-08-08 |
|  | South Remire-Montjoly Beach | FG-NE Coast | 16-1056 | Male | 4.8900923 | -52.2539511 | 2016-08-08 |
|  | South Remire-Montjoly Beach | FG-NE Coast | 16-1058 | Male | 4.8900923 | -52.2539511 | 2016-08-08 |
|  | South Remire-Montjoly Beach | FG-NE Coast | 16-1059 | Male | 4.8900923 | -52.2539511 | 2016-08-08 |
|  | South Remire-Montjoly Beach | FG-NE Coast | 16-1060 | Female | 4.8900923 | -52.2539511 | 2016-08-08 |
|  | South Remire-Montjoly Beach | FG-NE Coast | 16-1061 | Male | 4.8900923 | -52.2539511 | 2016-08-08 |
|  | South Remire-Montjoly Beach | FG-NE Coast | 16-1062 | Female | 4.8900923 | -52.2539511 | 2016-08-08 |
|  | South Remire-Montjoly Beach | FG-NE Coast | 16-1063 | Male | 4.8900923 | -52.2539511 | 2016-08-08 |
|  | South Remire-Montjoly Beach | FG-NE Coast | 16-1064 | Female | 4.8900923 | -52.2539511 | 2016-08-08 |
|  | South Remire-Montjoly Beach | FG-NE Coast | 16-1065 | Male | 4.8900923 | -52.2539511 | 2016-08-08 |
|  | South Remire-Montjoly Beach | FG-NE Coast | 16-1066 | Male | 4.8900923 | -52.2539511 | 2016-08-08 |

Table S2: Summary information of cane toads (*Rhinella marina*) used for genetic analysis in this study.

| **Site** | **Location** | **Environment** | **Sample ID** | **Sex** | **Tissue** | **Latitude** | **Longitude** | **Collection date** |
| --- | --- | --- | --- | --- | --- | --- | --- | --- |
| **French Guiana** | St. Georges Shoreline | Rainforest | RMF740 | Female | Toe | 3.895318343 | -51.79818347 | 2016-07-28 |
|  | St. Georges Shoreline | Rainforest | RMF741 | Female | Toe | 3.895318343 | -51.79818347 | 2016-07-28 |
|  | St. Georges Aerodrome | Rainforest | RMF744 | Male | Toe | 3.900456271 | -51.80153087 | 2016-07-28 |
|  | St. Georges Aerodrome | Rainforest | RMF749 | Female | Toe | 3.900456271 | -51.80153087 | 2016-07-28 |
|  | St. Georges Aerodrome | Rainforest | RMF743 | Male | Toe | 3.900456271 | -51.80153087 | 2016-07-28 |
|  | St. Georges Aerodrome | Rainforest | RMF746 | Male | Toe | 3.900456271 | -51.80153087 | 2016-07-28 |
|  | St. Georges Aerodrome | Rainforest | RMF745 | Male | Toe | 3.900456271 | -51.80153087 | 2016-07-28 |
|  | St. Georges Aerodrome | Rainforest | RMF750 | Male | Toe | 3.900456271 | -51.80153087 | 2016-07-28 |
|  | St George lumberyard | Rainforest | LY2 | Male | Toe | 3.9478833 | -51.851 | 2016 |
|  | St George lumberyard | Rainforest | LY1 | Male | Toe | 3.9479 | -51.851 | 2016 |
|  | Belizon wetland | Rainforest | J6 | Male | Toe | 4.3396 | -52.5142 | 2016 |
|  | Belizon wetland | Rainforest | J5 | Male | Toe | 4.3396 | -52.5142 | 2016 |
|  | Regina Marshes | Rainforest | RMF734 | Juvenile | Toe | 4.3625833 | -52.27863889 | 2016-07-27 |
|  | Tadpole puddles | Rainforest | Puddle | Male | Toe | 4.4910167 | -52.3467167 | 2016 |
|  | Kaw Marshes | Rainforest | RMF711 | Male | Toe | 4.5020833 | -52.057 | 2016-07-26 |
|  | Kaw Marshes | Rainforest | RMF712 | Male | Toe | 4.5180278 | -52.09777778 | 2016-07-26 |
|  | Kaw Marshes | Rainforest | RMF713 | Juvenile | Toe | 4.5271667 | -52.11927778 | 2016-07-26 |
|  | Kaw Marshes | Rainforest | RMF715 | Male | Toe | 4.5333333 | -52.12813889 | 2016-07-26 |
|  | Kaw Marshes | Rainforest | RMF714 | Male | Toe | 4.5333333 | -52.12813889 | 2016-07-26 |
|  | Kaw Lumberyard | Rainforest | RMF716 | Male | Toe | 4.5489722 | -52.17194444 | 2016-07-26 |
|  | Kaw Lumberyard | Rainforest | RMF717 | Male | Toe | 4.5489722 | -52.17194444 | 2016-07-26 |
|  | Kaw Marshes | Rainforest | RMF723 | Female | Toe | 4.5956111 | -52.26255556 | 2016-07-26 |
|  | South Remire-Montjoly Beach | Coast | RMF627 | Male | Toe | 4.890092298 | -52.25395113 | 2016-07-23 |
|  | South Remire-Montjoly Beach | Coast | RMF628 | Male | Toe | 4.890092298 | -52.25395113 | 2016-07-23 |
|  | Family Plaza | Coast | RMF668 | Male | Toe | 4.891353692 | -52.33383134 | 2016-07-23 |
|  | Family Plaza | Coast | RMF667 | Female | Toe | 4.891353692 | -52.33383134 | 2016-07-23 |
|  | Kourou Atlantis | Coast | RMF808 | Male | Toe | 5.15715438 | -52.64640525 | 2016-07-29 |
|  | Kourou Atlantis | Coast | RMF807 | Female | Toe | 5.15715438 | -52.64640525 | 2016-07-29 |
|  | Kourou Hotel Des Roches | Coast | RMF812 | Male | Toe | 5.160194 | -52.6274025 | 2016-07-29 |
|  | Kourou Hotel Des Roches | Coast | RMF814 | Male | Toe | 5.160194 | -52.6274025 | 2016-07-29 |
|  | Kourou Hotel Des Roches | Coast | RMF813 | Male | Toe | 5.160194 | -52.6274025 | 2016-07-29 |
|  | St Laurent forest | Rainforest | J2 | Unknown | Toe | 5.4872 | -53.9944 | 2016 |
|  | St Laurent forest | Rainforest | J3 | Unknown | Toe | 5.4872 | -53.9944 | 2016 |
|  | St Laurent school | Rainforest | J1 | Unknown | Toe | 5.4872 | -53.9944 | 2016 |
|  | St Laurent forest | Rainforest | J4 | Unknown | Toe | 5.4872 | -53.9944 | 2016 |
|  | Mana ditch | Coast | J10 | Unknown | Toe | 5.6694 | -53.7765 | 2016 |
|  | Yalimapo Municipal Building | Coast | RMF927 | Juvenile | Toe | 5.736114765 | -53.9112559 | 2016-08-04 |
|  | Yalimapo Municipal Building | Coast | RMF926 | Female | Toe | 5.736114765 | -53.9112559 | 2016-08-04 |
|  | Yalimapo Beach | Coast | RMF917 | Male | Toe | 5.746320083 | -53.94172579 | 2016-08-04 |
|  | Yalimapo Beach | Coast | RMF920 | Female | Toe | 5.746320083 | -53.94172579 | 2016-08-04 |
|  | Yalimapo Beach | Coast | RMF923 | Female | Toe | 5.746320083 | -53.94172579 | 2016-08-04 |
|  | Yalimapo Beach | Coast | RMF919 | Male | Toe | 5.746320083 | -53.94172579 | 2016-08-04 |
|  | Yalimapo Beach | Coast | RMF922 | Female | Toe | 5.746320083 | -53.94172579 | 2016-08-04 |
|  | Yalimapo Beach | Coast | RMF925 | Male | Toe | 5.746320083 | -53.94172579 | 2016-08-04 |
|  | Yalimapo Beach | Coast | RMF918 | Female | Toe | 5.746320083 | -53.94172579 | 2016-08-04 |
|  | Yalimapo Beach | Coast | RMF921 | Female | Toe | 5.746320083 | -53.94172579 | 2016-08-04 |
|  | Yalimapo Beach | Coast | RMF924 | Female | Toe | 5.746320083 | -53.94172579 | 2016-08-04 |
| **Australia** |  |  |  |  |  |  |  |  |
| Queensland | Croydon | AU_Group1 | RM308 | Female | Muscle | -18.206 | 142.24 | 2014-05-12 |
|  | Croydon | AU_Group1 | RM328 | Female | Muscle | -18.206 | 142.24 | 2014-05-12 |
|  | Croydon | AU_Group1 | RM332 | Male | Muscle | -18.206 | 142.24 | 2014-05-13 |
|  | Croydon | AU_Group1 | RM324 | Male | Muscle | -18.206 | 142.24 | 2014-05-12 |
|  | Croydon | AU_Group1 | RM326 | Female | Muscle | -18.206 | 142.24 | 2014-05-12 |
|  | Croydon | AU_Group1 | RM334 | Male | Muscle | -18.206 | 142.24 | 2014-05-13 |
|  | Croydon | AU_Group2 | RM330 | Female | Muscle | -18.206 | 142.24 | 2014-05-13 |
|  | Croydon | AU_Group2 | RM309 | Female | Muscle | -18.206 | 142.24 | 2014-05-12 |
|  | Croydon | AU_Group2 | RM329 | Female | Muscle | -18.206 | 142.24 | 2014-05-13 |
|  | Croydon | AU_Group1 | RM343 | Male | Muscle | -18.206 | 142.24 | 2014-05-13 |
|  | Croydon | AU_Group1 | RM325 | Male | Muscle | -18.206 | 142.24 | 2014-05-12 |
|  | Croydon | AU_Group1 | RM327 | Female | Muscle | -18.206 | 142.24 | 2014-05-12 |
|  | Croydon | AU_Group2 | RM345 | Male | Muscle | -18.206 | 142.24 | 2014-05-13 |
|  | Croydon | AU_Group1 | RM331 | Female | Muscle | -18.206 | 142.24 | 2014-05-13 |
|  | Croydon | AU_Group1 | RM333 | Male | Muscle | -18.206 | 142.24 | 2014-05-13 |
|  | Burketown | AU_Group2 | RM393 | Female | Muscle | -17.8522 | 139.633 | 2014-05-16 |
|  | Burketown | AU_Group1 | RM368 | Male | Muscle | -17.8522 | 139.633 | 2014-05-15 |
|  | Burketown | AU_Group1 | RM370 | Male | Muscle | -17.8522 | 139.633 | 2014-05-15 |
|  | Burketown | AU_Group2 | RM391 | Female | Muscle | -17.8522 | 139.633 | 2014-05-16 |
|  | Burketown | AU_Group1 | RM365 | Male | Muscle | -17.8522 | 139.633 | 2014-05-15 |
|  | Burketown | AU_Group2 | RM389 | Female | Muscle | -17.8522 | 139.633 | 2014-05-16 |
|  | Burketown | AU_Group2 | RM372 | Female | Muscle | -17.8522 | 139.633 | 2014-05-15 |
|  | Burketown | AU_Group1 | RM396 | Female | Muscle | -17.8522 | 139.633 | 2014-05-16 |
|  | Burketown | AU_Group1 | RM385 | Male | Muscle | -17.8522 | 139.633 | 2014-05-16 |
|  | Burketown | AU_Group1 | RM386 | Male | Muscle | -17.8522 | 139.633 | 2014-05-16 |
|  | Burketown | AU_Group2 | RM392 | Female | Muscle | -17.8522 | 139.633 | 2014-05-16 |
|  | Burketown | AU_Group2 | RM397 | Female | Muscle | -17.8522 | 139.633 | 2014-05-16 |
|  | Burketown | AU_Group1 | RM366 | Male | Muscle | -17.8522 | 139.633 | 2014-05-15 |
|  | Burketown | AU_Group1 | RM367 | Male | Muscle | -17.8522 | 139.633 | 2014-05-15 |
|  | Burketown | AU_Group2 | RM390 | Female | Muscle | -17.8522 | 139.633 | 2014-05-16 |
|  | Burketown | AU_Group1 | RM369 | Male | Muscle | -17.8522 | 139.633 | 2014-05-15 |
| Northern Territory | Timber Creek | AU_Group1 | RM599 | Male | Muscle | -15.6432 | 130.4666 | 2015-05-01 |
|  | Timber Creek | AU_Group1 | RM605 | Male | Muscle | -15.6432 | 130.4666 | 2015-05-01 |
|  | Timber Creek | AU_Group2 | RM601 | Female | Muscle | -15.6432 | 130.4666 | 2015-05-01 |
|  | Timber Creek | AU_Group2 | RM608 | Female | Muscle | -15.6432 | 130.4666 | 2015-05-01 |
|  | Timber Creek | AU_Group1 | RM595 | Male | Muscle | -15.6432 | 130.4666 | 2015-05-01 |
|  | Timber Creek | AU_Group2 | RM600 | Female | Muscle | -15.6432 | 130.4666 | 2015-05-01 |
|  | Timber Creek | AU_Group1 | RM593 | Male | Muscle | -15.6432 | 130.4666 | 2015-05-01 |
|  | Timber Creek | AU_Group1 | RM603 | Male | Muscle | -15.6432 | 130.4666 | 2015-05-01 |
|  | Timber Creek | AU_Group2 | RM607 | Female | Muscle | -15.6432 | 130.4666 | 2015-05-01 |
|  | Timber Creek | AU_Group2 | RM606 | Female | Muscle | -15.6432 | 130.4666 | 2015-05-01 |
|  | Timber Creek | AU_Group2 | RM598 | Female | Muscle | -15.6432 | 130.4666 | 2015-05-01 |
|  | Timber Creek | AU_Group1 | RM602 | Male | Muscle | -15.6432 | 130.4666 | 2015-05-01 |
|  | Timber Creek | AU_Group2 | RM604 | Female | Muscle | -15.6432 | 130.4666 | 2015-05-01 |
|  | Timber Creek | AU_Group2 | RM594 | Male | Muscle | -15.6432 | 130.4666 | 2015-05-01 |
|  | Timber Creek | AU_Group2 | RM597 | Female | Muscle | -15.6432 | 130.4666 | 2015-05-01 |

Table S3**:** Regression models showing the morphological (arm and leg length) differentiation between rainforest and coastal male cane toads (*Rhinella marina*) sampled in French Guiana. The main value and the number in parentheses represent regression coefficients and standard errors respectively. Coastal populations were considered as reference, so that a positive coefficient of POPRainforest means that rainforest toads had larger limbs (after correcting for SVL).

|  | Radioulnar | Humerus | Femur | Tibia |
| --- | --- | --- | --- | --- |
| (Intercept) | -1.21*** | -1.00*** | -1.13*** | -0.71*** |
|  | (0.12) | (0.10) | (0.10) | (0.08) |
| POPRainforest | 0.07*** | 0.07*** | 0.06*** | 0.04*** |
|  | (0.01) | (0.01) | (0.01) | (0.01) |
| LogSVL | 0.98*** | 0.99*** | 1.06*** | 0.95*** |
|  | (0.02) | (0.02) | (0.02) | (0.02) |
| N | 213 | 213 | 213 | 213 |
| R^2^ | 0.93 | 0.94 | 0.95 | 0.96 |
| *** *p* < 0.001; ** *p* < 0.01; * *p* < 0.05. | | | |  |

Table S4: Summary information of sequencing data for *Rhinella marina* used in this study, before and after variant filtering.

| **Pre-variant filtering** | **Results** | **Values** |
| --- | --- | --- |
| Sample selection and sequencing | Number of individuals collected | NA |
|  | Number of individuals intially sequenced | n = 93 |
| Sequence QC | Number of individuals who were successfully sequenced | n = 93 |
|  | Number of individuals who were with high quality sequences | n = 90 |
|  | Total number of reads prior to any filtering | 7.08 million reads / individual |
|  | Number of reads remaining after filtering for read quality | 6.92 million reads / individual |
| Mapped-read filtering | Number of reads that mapped | 5.8 million reads / individual |
|  | Number of reads remaining after filtering for mapping quality | 3.86 million reads / individual |
|  | Number of reads remaining after filtering for improperly paired reads | NA |
|  | Number of reads remaining after filtering for PCR duplicates | NA |

Table S5: sNMF cross-entropy values in α = 10, 100 and 1000.

|  | Regularisation parameter (α) | | |
| --- | --- | --- | --- |
| **K** | **10** | **100** | **1000** |
| **1** | 0.6014858 | 0.6019722 | 0.6008621 |
| **2** | 0.4987007 | 0.4992104 | 0.4994748 |
| **3** | 0.4875187 | **0.4871892** | 0.4894149 |
| **4** | 0.4894196 | 0.4898836 | 0.4904496 |
| **5** | 0.4935123 | 0.493955 | 0.4976588 |
| **6** | 0.4981235 | 0.5005973 | 0.5029818 |
| **7** | 0.5049839 | 0.5045715 | 0.5124338 |
| **8** | 0.508632 | 0.5126036 | 0.5198319 |
| **9** | 0.5190756 | 0.525134 | 0.5296716 |
| **10** | 0.535183 | 0.5344974 | 0.547608 |

Table S6: Percentage of variance explained by each PC axis by PCAdapt of all 90 native and invasive cane toads, a subset of French Guiana cane toads and a subset of invasive Australian cane toads.

|  | **All samples (Native & Invasive)** | **French Guiana (Native)** | **Australia (Invasive)** |
| --- | --- | --- | --- |
| PC | % variance explained | % variance explained | % variance explained |
| 1 | 29.28 | 12.42 | 7.65 |
| 2 | 8.04 | 6.97 | 5.3 |
| 3 | 4.43 | 5.15 | 4.67 |
| 4 | 3.19 | 4.09 | 4 |
| 5 | 2.54 | 3.91 | 3.53 |
| 6 | 2.32 | 3.74 | 3.4 |
| 7 | 2.23 | 3.68 | 3.37 |
| 8 | 2.19 | 3.43 | 3.32 |
| 9 | 2.05 | 3.33 | 3.25 |
| 10 | 2.03 | 3.19 | 3.12 |

Table S7: Putative outliers detected by BayPass between cane toads from the coast and rainforest in French Guiana. Gene ontology terms were assigned using DAVID.

| **Gene** | **Annotation from DAVID** | **GOTERM_BP_DIRECT** | **GOTERM_CC_DIRECT** | **GOTERM_MF_DIRECT** |
| --- | --- | --- | --- | --- |
| RMARP26297 | TPD52 like 1(TPD52L1) | GO:0000086~G2/M transition of mitotic cell cycle,GO:0043406~positive regulation of MAP kinase activity,GO:0046330~positive regulation of JNK cascade,GO:2001235~positive regulation of apoptotic signaling pathway, | GO:0005737~cytoplasm,GO:0048471~perinuclear region of cytoplasm, | GO:0005515~protein binding,GO:0042802~identical protein binding,GO:0042803~protein homodimerization activity, |
| RMARP00322 | cadherin 13(CDH13) | GO:0000902~cell morphogenesis,GO:0001938~positive regulation of endothelial cell proliferation,GO:0001954~positive regulation of cell-matrix adhesion,GO:0002040~sprouting angiogenesis,GO:0007043~cell-cell junction assembly,GO:0007156~homophilic cell adhesion via plasma membrane adhesion molecules,GO:0007162~negative regulation of cell adhesion,GO:0007266~Rho protein signal transduction,GO:0007275~multicellular organism development,GO:0008285~negative regulation of cell population proliferation,GO:0016339~calcium-dependent cell-cell adhesion via plasma membrane cell adhesion molecules,GO:0016601~Rac protein signal transduction,GO:0030032~lamellipodium assembly,GO:0030100~regulation of endocytosis,GO:0030335~positive regulation of cell migration,GO:0034332~adherens junction organization,GO:0042058~regulation of epidermal growth factor receptor signaling pathway,GO:0043542~endothelial cell migration,GO:0043616~keratinocyte proliferation,GO:0045944~positive regulation of transcription by RNA polymerase II,GO:0048661~positive regulation of smooth muscle cell proliferation,GO:0050850~positive regulation of calcium-mediated signaling,GO:0050927~positive regulation of positive chemotaxis,GO:0051668~localization within membrane,GO:0055096~low-density lipoprotein particle mediated signaling, | GO:0005615~extracellular space,GO:0005737~cytoplasm,GO:0005886~plasma membrane,GO:0005901~caveola,GO:0005912~adherens junction,GO:0009897~external side of plasma membrane,GO:0016020~membrane,GO:0016342~catenin complex,GO:0043005~neuron projection, | GO:0005509~calcium ion binding,GO:0030169~low-density lipoprotein particle binding,GO:0042802~identical protein binding,GO:0045296~cadherin binding,GO:0055100~adiponectin binding, |
| RMARP05438 | 5'-nucleotidase, cytosolic IIa(nt5c2a) | GO:0009117~nucleotide metabolic process,GO:0046037~GMP metabolic process, | GO:0005737~cytoplasm,GO:0005829~cytosol, | GO:0000166~nucleotide binding,GO:0003824~catalytic activity,GO:0005524~ATP binding,GO:0008253~5'-nucleotidase activity,GO:0016740~transferase activity,GO:0016787~hydrolase activity,GO:0046872~metal ion binding,GO:0050146~nucleoside phosphotransferase activity,GO:0106411~XMP 5'-nucleosidase activity, |
| RMARP06702 | glutamate-ammonia ligase | GO:0006542~glutamine biosynthetic process | GO:0005737~cytoplasm,GO:0005829~cytosol,GO:0005783~endoplasmic reticulum,GO:0005739~mitochondrion | GO:0005524~ATP binding,GO:0004356~glutamine synthetase activity,GO:0046872~metal ion binding |
| RMARP06703 | CABZ01027551.1 (Clone-based gene) | N/A | N/A | N/A |
| RMARP07870 | chloride channel accessory 1 gene 1 | N/A | GO:0005886~plasma membrane,GO:0016020~membrane, | GO:0005229~intracellular calcium-gated chloride channel activity,GO:0046872~metal ion binding,GO:0008237:metallopeptidase activity |
| RMARP08766 | caseinolytic mitochondrial matrix peptidase proteolytic subunit(clpp) | GO:0006508~proteolysis,GO:0006515~protein quality control for misfolded or incompletely synthesized proteins, | GO:0009368~endopeptidase Clp complex, | GO:0004176~ATP-dependent peptidase activity,GO:0004252~serine-type endopeptidase activity,GO:0008236~serine-type peptidase activity,GO:0051117~ATPase binding, |
| RMARP08767 | LOC100036665 | N/A | N/A | N/A |
| RMARP13710 | ELK4, ETS transcription factor(elk4) | GO:0006357~regulation of transcription by RNA polymerase II,GO:0030154~cell differentiation, | GO:0005634~nucleus, | GO:0000981~DNA-binding transcription factor activity, RNA polymerase II-specific,GO:0003677~DNA binding,GO:0003700~DNA-binding transcription factor activity,GO:0043565~sequence-specific DNA binding, |
| RMARP18826 | keratin 20(KRT20) | N/A | GO:0005882~intermediate filament, | GO:0005198~structural molecule activity, |
| RMARP22683 | solute carrier family 22 member 16(SLC22A16) | GO:0007338~single fertilization,GO:0015695~organic cation transport,GO:0030317~flagellated sperm motility,GO:0046717~acid secretion,GO:0055085~transmembrane transport, | GO:0005829~cytosol,GO:0005886~plasma membrane,GO:0016020~membrane, | GO:0005275~amine transmembrane transporter activity,GO:0015101~organic cation transmembrane transporter activity,GO:0015226~carnitine transmembrane transporter activity,GO:0015606~spermidine transmembrane transporter activity,GO:0022857~transmembrane transporter activity, |
| RMARP25757 | tissue factor pathway inhibitor(TFPI) | GO:0007596~blood coagulation,GO:0030195~negative regulation of blood coagulation,GO:0071383~cellular response to steroid hormone stimulus, | GO:0005576~extracellular region,GO:0005615~extracellular space,GO:0005783~endoplasmic reticulum,GO:0005886~plasma membrane,GO:0005901~caveola,GO:0009986~cell surface,GO:0098552~side of membrane, | GO:0004866~endopeptidase inhibitor activity,GO:0004867~serine-type endopeptidase inhibitor activity, |
| RMARP25760 | zgc:111983(zgc:111983) | GO:0008150~biological_process, | N/A | GO:0003674~molecular_function, |
| RMARP34118 | olfactory receptor family 13 subfamily AQ member 1D(or13aq1d) | GO:0007186~G protein-coupled receptor signaling pathway,GO:0007608~sensory perception of smell, | GO:0005886~plasma membrane,GO:0016020~membrane, | GO:0004930~G protein-coupled receptor activity,GO:0004984~olfactory receptor activity, |
| RMARP01098 | solute carrier family 5 member 11(SLC5A11) | GO:0006915~apoptotic process,GO:0008645~hexose transmembrane transport,GO:0015791~polyol transmembrane transport,GO:0015798~myo-inositol transport,GO:0035725~sodium ion transmembrane transport, | GO:0005886~plasma membrane,GO:0016020~membrane,GO:0016324~apical plasma membrane, | GO:0005365~myo-inositol transmembrane transporter activity,GO:0005412~D-glucose:sodium symporter activity,GO:0005515~protein binding,GO:0015166~polyol transmembrane transporter activity,GO:0022857~transmembrane transporter activity, |
| RMARP01099 | Glutamate receptor, ionotropic, delta 2 (Grid2) interacting protein | N/A | N/A | N/A |
| RMARP01630 | general transcription factor IIH subunit 2(gtf2h2) | GO:0006281~DNA repair,GO:0006289~nucleotide-excision repair,GO:0006351~DNA-templated transcription,GO:0006357~regulation of transcription by RNA polymerase II, | GO:0000439~transcription factor TFIIH core complex,GO:0005634~nucleus,GO:0005675~transcription factor TFIIH holo complex, | GO:0008270~zinc ion binding,GO:0046872~metal ion binding, |
| RMARP02023 | mitogen-activated protein kinase 12 (provisional)(mapk12 (provisional)) | GO:0035556~intracellular signal transduction, | GO:0005634~nucleus,GO:0005737~cytoplasm, | GO:0004674~protein serine/threonine kinase activity,GO:0004705~JUN kinase activity,GO:0004707~MAP kinase activity,GO:0005524~ATP binding, |
| RMARP02371 | solute carrier family 25 member 39(slc25a39) | GO:0006783~heme biosynthetic process,GO:1990542~mitochondrial transmembrane transport, | GO:0005739~mitochondrion,GO:0005743~mitochondrial inner membrane,GO:0016020~membrane, | GO:0046872~metal ion binding,GO:0051537~2 iron, 2 sulfur cluster binding, |
| RMARP03648 | calmodulin-lysine N-methyltransferase(camkmt) | GO:0032259~methylation, | GO:0005634~nucleus,GO:0005737~cytoplasm, | GO:0008168~methyltransferase activity,GO:0018025~calmodulin-lysine N-methyltransferase activity, |
| RMARP04086 | spermine oxidase(SMOX) | GO:0006596~polyamine biosynthetic process,GO:0006598~polyamine catabolic process,GO:0006805~xenobiotic metabolic process,GO:0046208~spermine catabolic process, | GO:0005634~nucleus,GO:0005654~nucleoplasm,GO:0005737~cytoplasm,GO:0005829~cytosol,GO:0031965~nuclear membrane,GO:0043231~intracellular membrane-bounded organelle, | GO:0016491~oxidoreductase activity,GO:0046592~polyamine oxidase activity,GO:0052901~spermine oxidase activity, |
| RMARP08242 | contactin 1(CNTN1) | GO:0007155~cell adhesion,GO:0007219~Notch signaling pathway,GO:0007411~axon guidance,GO:0007420~brain development,GO:0007626~locomotory behavior,GO:0010467~gene expression,GO:0010628~positive regulation of gene expression,GO:0010765~positive regulation of sodium ion transport,GO:0010976~positive regulation of neuron projection development,GO:0021549~cerebellum development,GO:0032289~central nervous system myelin formation,GO:0098609~cell-cell adhesion, | GO:0005886~plasma membrane,GO:0016020~membrane,GO:0030424~axon,GO:0042734~presynaptic membrane,GO:0045211~postsynaptic membrane,GO:0070062~extracellular exosome,GO:0098552~side of membrane,GO:0098978~glutamatergic synapse, | GO:0005515~protein binding,GO:0030246~carbohydrate binding,GO:0098632~cell-cell adhesion mediator activity, |
| RMARP08861 | family with sequence similarity 118, member B(Fam118b) | GO:0008150~biological_process, | GO:0005575~cellular_component,GO:0015030~Cajal body, | GO:0003674~molecular_function,GO:0042802~identical protein binding, |
| RMARP11598 | phenylalanyl-tRNA synthetase 2, mitochondrial(FARS2) | GO:0006412~translation,GO:0006418~tRNA aminoacylation for protein translation,GO:0006432~phenylalanyl-tRNA aminoacylation,GO:0008033~tRNA processing,GO:0043039~tRNA aminoacylation, | GO:0005737~cytoplasm,GO:0005739~mitochondrion,GO:0005759~mitochondrial matrix, | GO:0000049~tRNA binding,GO:0004826~phenylalanine-tRNA ligase activity,GO:0005515~protein binding,GO:0005524~ATP binding, |
| RMARP12293 | Family with sequence similarity 169 member B (FAM169B) | N/A | N/A | N/A |
| RMARP18990 | si:dkey-21a6.5(si:dkey-21a6.5) | N/A | GO:0016020~membrane, | N/A |
| RMARP20413 | ubiquitin specific peptidase 7 (herpes virus-associated)(usp7) | GO:0006508~proteolysis,GO:0016579~protein deubiquitination,GO:0031647~regulation of protein stability,GO:1904888~cranial skeletal system development, | GO:0005634~nucleus,GO:0005829~cytosol, | GO:0004843~cysteine-type deubiquitinase activity,GO:0008234~cysteine-type peptidase activity, |
| RMARP20549 | Novel gene | N/A | N/A | N/A |
| RMARP21138 | neuron navigator 3(NAV3) | GO:0007026~negative regulation of microtubule depolymerization,GO:0007399~nervous system development,GO:0022008~neurogenesis,GO:0030336~negative regulation of cell migration,GO:0031116~positive regulation of microtubule polymerization,GO:0032703~negative regulation of interleukin-2 production, | GO:0005640~nuclear outer membrane,GO:1990752~microtubule end, | GO:0005524~ATP binding,GO:0008017~microtubule binding,GO:0016887~ATP hydrolysis activity, |
| RMARP21296 | ALG9, alpha-1,2-mannosyltransferase | N/A | N/A | N/A |
| RMARP22151 | synaptophysin(SYP) | GO:0006897~endocytosis,GO:0010807~regulation of synaptic vesicle priming,GO:0016188~synaptic vesicle maturation,GO:0048168~regulation of neuronal synaptic plasticity,GO:0048169~regulation of long-term neuronal synaptic plasticity,GO:0048172~regulation of short-term neuronal synaptic plasticity,GO:0048488~synaptic vesicle endocytosis,GO:0048499~synaptic vesicle membrane organization,GO:2000300~regulation of synaptic vesicle exocytosis,GO:2000474~regulation of opioid receptor signaling pathway, | GO:0008021~synaptic vesicle,GO:0016020~membrane,GO:0030672~synaptic vesicle membrane,GO:0031594~neuromuscular junction,GO:0042734~presynaptic membrane,GO:0043005~neuron projection,GO:0043195~terminal bouton,GO:0048471~perinuclear region of cytoplasm,GO:0048786~presynaptic active zone,GO:0060076~excitatory synapse,GO:0098685~Schaffer collateral - CA1 synapse, | GO:0005515~protein binding,GO:0015485~cholesterol binding,GO:0042169~SH2 domain binding,GO:0042802~identical protein binding, |
| RMARP22152 | proteasome subunit alpha 5(psma5) | GO:0043161~proteasome-mediated ubiquitin-dependent protein catabolic process, | GO:0000502~proteasome complex,GO:0005634~nucleus,GO:0005737~cytoplasm,GO:0005839~proteasome core complex,GO:0019773~proteasome core complex, alpha-subunit complex, | N/A |
| RMARP22535 | CU694264.1 (Clone-based gene) | N/A | N/A | N/A |
| RMARP22536 | dCTP pyrophosphatase 1(Dctpp1) | GO:0006253~dCTP catabolic process,GO:0009143~nucleoside triphosphate catabolic process,GO:0042262~DNA protection, | GO:0005634~nucleus,GO:0005654~nucleoplasm,GO:0005739~mitochondrion,GO:0005829~cytosol, | GO:0000287~magnesium ion binding,GO:0016462~pyrophosphatase activity,GO:0032556~pyrimidine deoxyribonucleotide binding,GO:0042802~identical protein binding,GO:0047429~nucleoside triphosphate diphosphatase activity,GO:0047840~dCTP diphosphatase activity, |
| RMARP22537 | quinolinate phosphoribosyltransferase(qprt) | GO:0009435~NAD biosynthetic process,GO:0019363~pyridine nucleotide biosynthetic process,GO:0034213~quinolinate catabolic process, | GO:0005737~cytoplasm, | GO:0004514~nicotinate-nucleotide diphosphorylase (carboxylating) activity,GO:0016757~glycosyltransferase activity, |
| RMARP23426 | ring finger protein 217(rnf217) | GO:0006313~DNA transposition,GO:0006511~ubiquitin-dependent protein catabolic process,GO:0015074~DNA integration,GO:0016567~protein ubiquitination, | GO:0000151~ubiquitin ligase complex,GO:0005737~cytoplasm,GO:0016020~membrane, | GO:0003677~DNA binding,GO:0004842~ubiquitin-protein transferase activity,GO:0008270~zinc ion binding,GO:0016740~transferase activity,GO:0031624~ubiquitin conjugating enzyme binding,GO:0046872~metal ion binding,GO:0061630~ubiquitin protein ligase activity, |
| RMARP23490 | Novel gene | N/A | N/A | N/A |
| RMARP24128 | cerebellar degeneration-related protein 2-like (CDR2L) | N/A | N/A | N/A |
| RMARP24129 | peroxisomal biogenesis factor 11 beta(Pex11b) | GO:0007031~peroxisome organization,GO:0007165~signal transduction,GO:0016559~peroxisome fission,GO:0044375~regulation of peroxisome size, | GO:0005654~nucleoplasm,GO:0005739~mitochondrion,GO:0005777~peroxisome,GO:0005778~peroxisomal membrane,GO:0032991~protein-containing complex,GO:0043231~intracellular membrane-bounded organelle, | GO:0042802~identical protein binding,GO:0042803~protein homodimerization activity, |
| RMARP24130 | HID1 domain containing(hid1) | N/A | GO:0000138~Golgi trans cisterna,GO:0005797~Golgi medial cisterna,GO:0016020~membrane, | N/A |
| RMARP27343 | protein kinase, AMP-activated, gamma 2 non-catalytic subunit(prkag2) | GO:0006110~regulation of glycolytic process,GO:0006633~fatty acid biosynthetic process,GO:0042149~cellular response to glucose starvation,GO:0043609~regulation of carbon utilization,GO:0045722~positive regulation of gluconeogenesis, | GO:0005634~nucleus,GO:0005737~cytoplasm,GO:0031588~nucleotide-activated protein kinase complex, | GO:0016208~AMP binding,GO:0016301~kinase activity,GO:0019887~protein kinase regulator activity,GO:0019901~protein kinase binding, |
| RMARP29017 | Rho GTPase activating protein 18(arhgap18) | GO:0007165~signal transduction,GO:0030833~regulation of actin filament polymerization,GO:0051056~regulation of small GTPase mediated signal transduction, | GO:0005737~cytoplasm, | GO:0005096~GTPase activator activity, |
| RMARP29019 | mitochondrial trans-2-enoyl-CoA reductase(mecr) | GO:0006629~lipid metabolic process,GO:0006631~fatty acid metabolic process,GO:0006633~fatty acid biosynthetic process, | GO:0005739~mitochondrion, | GO:0016491~oxidoreductase activity,GO:0141148~enoyl-[acyl-carrier-protein] reductase (NADPH) activity, |
| RMARP31485 | ABRA C-terminal like (ABRACL) | N/A | N/A | N/A |
| RMARP31979 | chloride intracellular channel 4(clic4) | GO:0006821~chloride transport,GO:0034220~monoatomic ion transmembrane transport, | GO:0005634~nucleus,GO:0005737~cytoplasm,GO:0005739~mitochondrion,GO:0005813~centrosome,GO:0005886~plasma membrane,GO:0016020~membrane,GO:0030659~cytoplasmic vesicle membrane,GO:0034707~chloride channel complex,GO:0070161~anchoring junction, | GO:0005254~chloride channel activity,GO:0016491~oxidoreductase activity, |
| RMARP31980 | ATP-binding cassette, sub-family A (ABC1), member 13 | N/A | N/A | N/A |
| RMARP32718 | leucine rich repeats and calponin homology domain containing 2(LRCH2) | N/A | N/A | GO:0005515~protein binding, |
| RMARP32721 | Predicted gene, 40193 | N/A | N/A | N/A |
| RMARP35142 | protein kinase N2(pkn2) | GO:0007165~signal transduction,GO:0035556~intracellular signal transduction, | GO:0005634~nucleus,GO:0005737~cytoplasm,GO:0030496~midbody,GO:0032154~cleavage furrow, | GO:0004674~protein serine/threonine kinase activity,GO:0004697~diacylglycerol-dependent serine/threonine kinase activity,GO:0004698~calcium,diacylglycerol-dependent serine/threonine kinase activity,GO:0004699~diacylglycerol-dependent, calcium-independent serine/threonine kinase activity,GO:0005524~ATP binding,GO:0031267~small GTPase binding, |

Table S8: Putative outliers detected by BayPass between Croydon and Timber Creek in Australia. Gene ontology terms were assigned using DAVID.

| **Gene** | **Annotation from DAVID** | **GOTERM_BP_DIRECT** | **GOTERM_CC_DIRECT** | **GOTERM_MF_DIRECT** |
| --- | --- | --- | --- | --- |
| RMARP00876 | DnaJ heat shock protein family (Hsp40) member C24(dnajc24) | GO:0002098~tRNA wobble uridine modification, | GO:0005634~nucleus,GO:0005737~cytoplasm,GO:0016020~membrane,GO:0033588~elongator holoenzyme complex, | GO:0001671~ATPase activator activity,GO:0008198~ferrous iron binding, |
| RMARP03477 | cell adhesion molecule 2(CADM2) | GO:0007156~homophilic cell adhesion via plasma membrane adhesion molecules, | GO:0005886~plasma membrane,GO:0030424~axon,GO:0032809~neuronal cell body membrane,GO:0045202~synapse, | N/A |
| RMARP04699 | denticleless E3 ubiquitin protein ligase homolog(DTL) | GO:0000209~protein polyubiquitination,GO:0006260~DNA replication,GO:0006511~ubiquitin-dependent protein catabolic process,GO:0006513~protein monoubiquitination,GO:0006974~DNA damage response,GO:0007095~mitotic G2 DNA damage checkpoint signaling,GO:0009411~response to UV,GO:0019985~translesion synthesis,GO:0043161~proteasome-mediated ubiquitin-dependent protein catabolic process,GO:0048511~rhythmic process,GO:0051726~regulation of cell cycle, | GO:0005634~nucleus,GO:0005694~chromosome,GO:0005737~cytoplasm,GO:0005813~centrosome,GO:0031464~Cul4A-RING E3 ubiquitin ligase complex,GO:0031465~Cul4B-RING E3 ubiquitin ligase complex, | GO:0005515~protein binding,GO:0030674~protein-macromolecule adaptor activity, |
| RMARP04700 | integrator complex subunit 7(ints7) | GO:0002088~lens development in camera-type eye,GO:0016180~snRNA processing,GO:0034472~snRNA 3'-end processing, | GO:0005634~nucleus,GO:0032039~integrator complex, | N/A |
| RMARP04803 | Rho GTPase activating protein 23(ARHGAP23) | GO:0007165~signal transduction,GO:0051056~regulation of small GTPase mediated signal transduction, | GO:0005829~cytosol,GO:0070062~extracellular exosome, | GO:0005096~GTPase activator activity, |
| RMARP05494 | Succinate dehydrogenase assembly factor 4, mitochondrial(SDHAF4) | GO:0034553~mitochondrial respiratory chain complex II assembly, | GO:0005739~mitochondrion, | N/A |
| RMARP05495 | keratin associated protein 23-1(KRTAP23-1) | N/A | GO:0005829~cytosol,GO:0005882~intermediate filament, | GO:0005515~protein binding, |
| RMARP08740 | phospholipase C beta 3(plcb3) | GO:0007165~signal transduction,GO:0007186~G protein-coupled receptor signaling pathway,GO:0016042~lipid catabolic process,GO:0035556~intracellular signal transduction,GO:0046488~phosphatidylinositol metabolic process,GO:0048015~phosphatidylinositol-mediated signaling,GO:0051209~release of sequestered calcium ion into cytosol, | GO:0005737~cytoplasm, | GO:0004435~phosphatidylinositol phospholipase C activity,GO:0005509~calcium ion binding,GO:0005516~calmodulin binding,GO:0016787~hydrolase activity,GO:0046872~metal ion binding, |
| RMARP08745 | DENN domain containing 1C(dennd1c) | GO:0006897~endocytosis,GO:0032456~endocytic recycling, | GO:0005829~cytosol,GO:0031410~cytoplasmic vesicle,GO:0043231~intracellular membrane-bounded organelle, | GO:0005085~guanyl-nucleotide exchange factor activity,GO:1901981~phosphatidylinositol phosphate binding, |
| RMARP11194 | polypeptide N-acetylgalactosaminyltransferase 14(GALNT14) | GO:0006486~protein glycosylation, | GO:0000139~Golgi membrane,GO:0005794~Golgi apparatus, | GO:0004653~polypeptide N-acetylgalactosaminyltransferase activity,GO:0030246~carbohydrate binding, |
| RMARP11672 | TATA-box binding protein associated factor 9b(taf9b) | GO:0006352~DNA-templated transcription initiation,GO:0051123~RNA polymerase II preinitiation complex assembly, | GO:0000124~SAGA complex,GO:0005634~nucleus,GO:0005669~transcription factor TFIID complex, | GO:0003713~transcription coactivator activity,GO:0016251~RNA polymerase II general transcription initiation factor activity,GO:0046982~protein heterodimerization activity, |
| RMARP11673 | germ cell nuclear acidic peptidase(GCNA) | GO:0006259~DNA metabolic process,GO:0006950~response to stress, | GO:0005634~nucleus, | N/A |
| RMARP12056 | CF transmembrane conductance regulator(CFTR) | GO:0006338~chromatin remodeling,GO:0006695~cholesterol biosynthetic process,GO:0006904~vesicle docking involved in exocytosis,GO:0015701~bicarbonate transport,GO:0030301~cholesterol transport,GO:0034976~response to endoplasmic reticulum stress,GO:0035377~transepithelial water transport,GO:0035774~positive regulation of insulin secretion involved in cellular response to glucose stimulus,GO:0045921~positive regulation of exocytosis,GO:0048240~sperm capacitation,GO:0050891~multicellular organismal-level water homeostasis,GO:0051454~intracellular pH elevation,GO:0051649~establishment of localization in cell,GO:0055085~transmembrane transport,GO:0060081~membrane hyperpolarization,GO:0070175~positive regulation of enamel mineralization,GO:0071320~cellular response to cAMP,GO:0097186~amelogenesis,GO:0140588~chromatin looping,GO:1902161~positive regulation of cyclic nucleotide-gated ion channel activity,GO:1902476~chloride transmembrane transport,GO:1902943~positive regulation of voltage-gated chloride channel activity,GO:1904322~cellular response to forskolin, | GO:0005634~nucleus,GO:0005737~cytoplasm,GO:0005765~lysosomal membrane,GO:0005769~early endosome,GO:0005789~endoplasmic reticulum membrane,GO:0005829~cytosol,GO:0005886~plasma membrane,GO:0009986~cell surface,GO:0010008~endosome membrane,GO:0016020~membrane,GO:0016324~apical plasma membrane,GO:0030660~Golgi-associated vesicle membrane,GO:0030669~clathrin-coated endocytic vesicle membrane,GO:0031901~early endosome membrane,GO:0032991~protein-containing complex,GO:0034707~chloride channel complex,GO:0055037~recycling endosome,GO:0055038~recycling endosome membrane, | GO:0000166~nucleotide binding,GO:0003689~DNA clamp loader activity,GO:0005254~chloride channel activity,GO:0005260~intracellularly ATP-gated chloride channel activity,GO:0005515~protein binding,GO:0005524~ATP binding,GO:0015106~bicarbonate transmembrane transporter activity,GO:0015108~chloride transmembrane transporter activity,GO:0016787~hydrolase activity,GO:0016853~isomerase activity,GO:0016887~ATP hydrolysis activity,GO:0017081~chloride channel regulator activity,GO:0019869~chloride channel inhibitor activity,GO:0019899~enzyme binding,GO:0030165~PDZ domain binding,GO:0042626~ATPase-coupled transmembrane transporter activity,GO:0043225~ATPase-coupled inorganic anion transmembrane transporter activity,GO:0051087~protein-folding chaperone binding,GO:0061775~cohesin loader activity,GO:0071889~14-3-3 protein binding,GO:0106138~Sec61 translocon complex binding,GO:0140359~ABC-type transporter activity,GO:0140584~chromatin extrusion motor activity,GO:0140665~ATP-dependent H3-H4 histone complex chaperone activity,GO:0140849~ATP-dependent H2AZ histone chaperone activity, |
| RMARP12573 | SLIT-ROBO Rho GTPase activating protein 1(srgap1) | GO:0007165~signal transduction,GO:0030336~negative regulation of cell migration, | GO:0005737~cytoplasm,GO:0005886~plasma membrane, | GO:0005096~GTPase activator activity, |
| RMARP14634 | regulating synaptic membrane exocytosis 2(rims2) | GO:0006886~intracellular protein transport,GO:0006887~exocytosis, | GO:0016020~membrane,GO:0045202~synapse, | GO:0031267~small GTPase binding,GO:0046872~metal ion binding, |
| RMARP14639 | LDL receptor related protein 12(lrp12) | N/A | GO:0005886~plasma membrane, | GO:0008236~serine-type peptidase activity, |
| RMARP14639 | dihydropyrimidinase like 5(DPYSL5) | GO:0007165~signal transduction,GO:0007399~nervous system development,GO:0007411~axon guidance,GO:0050774~negative regulation of dendrite morphogenesis, | GO:0005737~cytoplasm,GO:0005829~cytosol,GO:0030425~dendrite,GO:0032991~protein-containing complex,GO:0043025~neuronal cell body,GO:0098978~glutamatergic synapse, | GO:0005515~protein binding,GO:0016810~hydrolase activity, acting on carbon-nitrogen (but not peptide) bonds,GO:0016812~hydrolase activity, acting on carbon-nitrogen (but not peptide) bonds, in cyclic amides, |
| RMARP16030 | Zmp:0000001267 (H2TTY7_TAKRU) | N/A | GO:0016020~membrane, | GO:0015179~L-amino acid transmembrane transporter activity, |
| RMARP16031 | slc7a13 | N/A | N/A | N/A |
| RMARP16769 | synapsin II(Syn2) | GO:0007269~neurotransmitter secretion,GO:0017156~calcium-ion regulated exocytosis,GO:0050808~synapse organization,GO:0097091~synaptic vesicle clustering,GO:0099504~synaptic vesicle cycle, | GO:0005886~plasma membrane,GO:0008021~synaptic vesicle,GO:0014069~postsynaptic density,GO:0030672~synaptic vesicle membrane,GO:0031201~SNARE complex,GO:0043209~myelin sheath,GO:0045202~synapse,GO:0098685~Schaffer collateral - CA1 synapse,GO:0098850~extrinsic component of synaptic vesicle membrane,GO:0098978~glutamatergic synapse, | GO:0005515~protein binding,GO:0005524~ATP binding,GO:0042802~identical protein binding,GO:0048306~calcium-dependent protein binding, |
| RMARP16772 | F-box protein 7(FBXO7) | GO:0000422~autophagy of mitochondrion,GO:0006626~protein targeting to mitochondrion,GO:0010975~regulation of neuron projection development,GO:0030098~lymphocyte differentiation,GO:0031647~regulation of protein stability,GO:0040012~regulation of locomotion,GO:0043161~proteasome-mediated ubiquitin-dependent protein catabolic process,GO:0045620~negative regulation of lymphocyte differentiation,GO:0070936~protein K48-linked ubiquitination,GO:1901526~positive regulation of mitophagy,GO:1903377~negative regulation of oxidative stress-induced neuron intrinsic apoptotic signaling pathway,GO:1903599~positive regulation of autophagy of mitochondrion,GO:2000134~negative regulation of G1/S transition of mitotic cell cycle, | GO:0005654~nucleoplasm,GO:0005739~mitochondrion,GO:0005829~cytosol,GO:0019005~SCF ubiquitin ligase complex,GO:0097409~glial cytoplasmic inclusion,GO:0097414~classical Lewy body,GO:0097462~Lewy neurite,GO:1990037~Lewy body core,GO:1990038~Lewy body corona, | GO:0019901~protein kinase binding,GO:0031625~ubiquitin protein ligase binding,GO:0043130~ubiquitin binding,GO:0046982~protein heterodimerization activity,GO:1990756~ubiquitin-like ligase-substrate adaptor activity, |
| RMARP16773 | RNA 2',3'-cyclic phosphate and 5'-OH ligase(RTCB) | GO:0006388~tRNA splicing, via endonucleolytic cleavage and ligation, | GO:0005634~nucleus,GO:0005635~nuclear envelope,GO:0005654~nucleoplasm,GO:0005789~endoplasmic reticulum membrane,GO:0005829~cytosol,GO:0072669~tRNA-splicing ligase complex, | GO:0003972~RNA ligase (ATP) activity,GO:0005525~GTP binding,GO:0017166~vinculin binding,GO:0046872~metal ion binding,GO:0170057~RNA ligase (GTP) activity, |
| RMARP16955 | nuclear factor I A(NFIA) | GO:0000902~cell morphogenesis,GO:0006260~DNA replication,GO:0006357~regulation of transcription by RNA polymerase II,GO:0009611~response to wounding,GO:0010458~exit from mitosis,GO:0010467~gene expression,GO:0014009~glial cell proliferation,GO:0021780~glial cell fate specification,GO:0030509~BMP signaling pathway,GO:0035108~limb morphogenesis,GO:0045893~positive regulation of DNA-templated transcription,GO:0048665~neuron fate specification,GO:0051216~cartilage development,GO:0060041~retina development in camera-type eye,GO:0060074~synapse maturation,GO:0061351~neural precursor cell proliferation,GO:0072189~ureter development, | GO:0005634~nucleus,GO:0005654~nucleoplasm,GO:0030054~cell junction, | GO:0000978~RNA polymerase II cis-regulatory region sequence-specific DNA binding,GO:0000981~DNA-binding transcription factor activity, RNA polymerase II-specific,GO:0001228~DNA-binding transcription activator activity, RNA polymerase II-specific,GO:0003677~DNA binding,GO:0003682~chromatin binding,GO:0003700~DNA-binding transcription factor activity,GO:0140297~DNA-binding transcription factor binding, |
| RMARP16961 | A0A674NJ45_TAKRU | N/A | N/A | GO:0003676~nucleic acid binding, |
| RMARP17011 | catenin alpha 2(CTNNA2) | GO:0007010~cytoskeleton organization,GO:0007155~cell adhesion,GO:0007409~axonogenesis,GO:0010975~regulation of neuron projection development,GO:0016477~cell migration,GO:0021942~radial glia guided migration of Purkinje cell,GO:0030154~cell differentiation,GO:0034316~negative regulation of Arp2/3 complex-mediated actin nucleation,GO:0048813~dendrite morphogenesis,GO:0048854~brain morphogenesis,GO:0051823~regulation of synapse structural plasticity,GO:0060134~prepulse inhibition,GO:0098609~cell-cell adhesion,GO:2001222~regulation of neuron migration, | GO:0005634~nucleus,GO:0005737~cytoplasm,GO:0005829~cytosol,GO:0005912~adherens junction,GO:0015629~actin cytoskeleton,GO:0016020~membrane,GO:0016342~catenin complex,GO:0030424~axon, | GO:0005200~structural constituent of cytoskeleton,GO:0005515~protein binding,GO:0008013~beta-catenin binding,GO:0045296~cadherin binding,GO:0051015~actin filament binding, |
| RMARP17012 | ATP binding cassette subfamily B member 8(ABCB8) | GO:0006813~potassium ion transport,GO:0006884~cell volume homeostasis,GO:0015833~peptide transport,GO:0055085~transmembrane transport,GO:0071805~potassium ion transmembrane transport,GO:0140141~mitochondrial potassium ion transmembrane transport, | GO:0005654~nucleoplasm,GO:0005730~nucleolus,GO:0005739~mitochondrion,GO:0005743~mitochondrial inner membrane,GO:0016020~membrane,GO:0031966~mitochondrial membrane,GO:0043190~ATP-binding cassette (ABC) transporter complex,GO:0062157~mitochondrial ATP-gated potassium channel complex, | GO:0005515~protein binding,GO:0005524~ATP binding,GO:0015440~ABC-type peptide transporter activity,GO:0016887~ATP hydrolysis activity,GO:0042626~ATPase-coupled transmembrane transporter activity,GO:0140359~ABC-type transporter activity, |
| RMARP18378 | nucleoporin 50(NUP50) | GO:0001841~neural tube formation,GO:0006606~protein import into nucleus,GO:0006913~nucleocytoplasmic transport,GO:0051028~mRNA transport, | GO:0005635~nuclear envelope,GO:0005643~nuclear pore,GO:0005654~nucleoplasm,GO:0031965~nuclear membrane, | GO:0005515~protein binding, |
| RMARP19136 | protein tyrosine phosphatase non-receptor type 2(PTPN2) | GO:0000122~negative regulation of transcription by RNA polymerase II,GO:0006338~chromatin remodeling,GO:0008285~negative regulation of cell population proliferation,GO:0008286~insulin receptor signaling pathway,GO:0010804~negative regulation of tumor necrosis factor-mediated signaling pathway,GO:0010888~negative regulation of lipid storage,GO:0030183~B cell differentiation,GO:0030217~T cell differentiation,GO:0030218~erythrocyte differentiation,GO:0035335~peptidyl-tyrosine dephosphorylation,GO:0038020~insulin receptor recycling,GO:0042059~negative regulation of epidermal growth factor receptor signaling pathway,GO:0042532~negative regulation of tyrosine phosphorylation of STAT protein,GO:0042593~glucose homeostasis,GO:0045650~negative regulation of macrophage differentiation,GO:0045722~positive regulation of gluconeogenesis,GO:0046426~negative regulation of receptor signaling pathway via JAK-STAT,GO:0046627~negative regulation of insulin receptor signaling pathway,GO:0050728~negative regulation of inflammatory response,GO:0050860~negative regulation of T cell receptor signaling pathway,GO:0050922~negative regulation of chemotaxis,GO:0060334~regulation of type II interferon-mediated signaling pathway,GO:0060336~negative regulation of type II interferon-mediated signaling pathway,GO:0060339~negative regulation of type I interferon-mediated signaling pathway,GO:0070104~negative regulation of interleukin-6-mediated signaling pathway,GO:0070373~negative regulation of ERK1 and ERK2 cascade,GO:1902202~regulation of hepatocyte growth factor receptor signaling pathway,GO:1902206~negative regulation of interleukin-2-mediated signaling pathway,GO:1902215~negative regulation of interleukin-4-mediated signaling pathway,GO:1902227~negative regulation of macrophage colony-stimulating factor signaling pathway,GO:1902233~negative regulation of positive thymic T cell selection,GO:1902237~positive regulation of endoplasmic reticulum stress-induced intrinsic apoptotic signaling pathway,GO:1903899~positive regulation of PERK-mediated unfolded protein response,GO:2000587~negative regulation of platelet-derived growth factor receptor-beta signaling pathway, | GO:0005634~nucleus,GO:0005654~nucleoplasm,GO:0005737~cytoplasm,GO:0005783~endoplasmic reticulum,GO:0005793~endoplasmic reticulum-Golgi intermediate compartment,GO:0005829~cytosol,GO:0005886~plasma membrane,GO:0016020~membrane,GO:0031904~endosome lumen, | GO:0004725~protein tyrosine phosphatase activity,GO:0004726~non-membrane spanning protein tyrosine phosphatase activity,GO:0005178~integrin binding,GO:0005515~protein binding,GO:0019901~protein kinase binding,GO:0019905~syntaxin binding,GO:0030946~protein tyrosine phosphatase activity, metal-dependent,GO:0030971~receptor tyrosine kinase binding,GO:0097677~STAT family protein binding,GO:0140793~histone H2AXY142 phosphatase activity, |
| RMARP19808 | glial cells missing transcription factor 1(GCM1) | GO:0000768~syncytium formation by plasma membrane fusion,GO:0006355~regulation of DNA-templated transcription,GO:0006357~regulation of transcription by RNA polymerase II,GO:0006366~transcription by RNA polymerase II,GO:0009653~anatomical structure morphogenesis,GO:0042063~gliogenesis,GO:0045893~positive regulation of DNA-templated transcription,GO:0045944~positive regulation of transcription by RNA polymerase II,GO:0060018~astrocyte fate commitment,GO:0060143~positive regulation of syncytium formation by plasma membrane fusion,GO:0060670~branching involved in labyrinthine layer morphogenesis,GO:0060706~cell differentiation involved in embryonic placenta development,GO:0060800~regulation of cell differentiation involved in embryonic placenta development, | GO:0000785~chromatin,GO:0005634~nucleus,GO:0005667~transcription regulator complex, | GO:0000978~RNA polymerase II cis-regulatory region sequence-specific DNA binding,GO:0000981~DNA-binding transcription factor activity, RNA polymerase II-specific,GO:0001228~DNA-binding transcription activator activity, RNA polymerase II-specific,GO:0003677~DNA binding,GO:0003700~DNA-binding transcription factor activity,GO:0005515~protein binding,GO:0008270~zinc ion binding,GO:0042826~histone deacetylase binding,GO:1990837~sequence-specific double-stranded DNA binding, |
| RMARP19812 | glutathione transferase-like(LOC100190440) | GO:0006749~glutathione metabolic process,GO:0006805~xenobiotic metabolic process, | N/A | GO:0004364~glutathione transferase activity, |
| RMARP19816 | microcephalin 1(mcph1) | GO:0000278~mitotic cell cycle,GO:0006281~DNA repair, | GO:0005634~nucleus,GO:0016020~membrane, | GO:0003677~DNA binding,GO:0004842~ubiquitin-protein transferase activity,GO:0008270~zinc ion binding,GO:0016746~acyltransferase activity, |
| RMARP19817 | Novel gene | N/A | N/A | N/A |
| RMARP19827 | Novel gene | N/A | N/A | N/A |
| RMARP20457 | Chromosome 1 C18orf63 homolog | N/A | N/A | N/A |
| RMARP20463 | translocase of inner mitochondrial membrane 21 homolog(timm21) | GO:0015031~protein transport,GO:0030150~protein import into mitochondrial matrix, | GO:0005739~mitochondrion,GO:0005743~mitochondrial inner membrane,GO:0005744~TIM23 mitochondrial import inner membrane translocase complex, | N/A |
| RMARP20476 | diacylglycerol kinase iota(DGKI) | GO:0006654~phosphatidic acid biosynthetic process,GO:0007200~phospholipase C-activating G protein-coupled receptor signaling pathway,GO:0007265~Ras protein signal transduction,GO:0007269~neurotransmitter secretion,GO:0030168~platelet activation,GO:0035556~intracellular signal transduction,GO:0046339~diacylglycerol metabolic process,GO:0046486~glycerolipid metabolic process,GO:0046579~positive regulation of Ras protein signal transduction,GO:0046834~lipid phosphorylation,GO:0046959~habituation,GO:0051966~regulation of synaptic transmission, glutamatergic,GO:0060079~excitatory postsynaptic potential,GO:0099171~presynaptic modulation of chemical synaptic transmission,GO:1900452~regulation of long-term synaptic depression, | GO:0005634~nucleus,GO:0005737~cytoplasm,GO:0005829~cytosol,GO:0005886~plasma membrane,GO:0014069~postsynaptic density,GO:0016020~membrane,GO:0030424~axon,GO:0030672~synaptic vesicle membrane,GO:0032045~guanyl-nucleotide exchange factor complex,GO:0032991~protein-containing complex,GO:0043197~dendritic spine,GO:0045202~synapse,GO:0048471~perinuclear region of cytoplasm,GO:0097060~synaptic membrane,GO:0098685~Schaffer collateral - CA1 synapse,GO:0098793~presynapse,GO:0098794~postsynapse,GO:0098978~glutamatergic synapse, | GO:0004143~ATP-dependent diacylglycerol kinase activity,GO:0005095~GTPase inhibitor activity,GO:0005515~protein binding,GO:0005524~ATP binding,GO:0031267~small GTPase binding,GO:0046872~metal ion binding, |
| RMARP23682 | tctex1 domain-containing protein 2(LOC100495666) | GO:0007018~microtubule-based movement, | GO:0005737~cytoplasm,GO:0005868~cytoplasmic dynein complex, | GO:0045505~dynein intermediate chain binding, |
| RMARP24048 | family with sequence similarity 236, member F(Fam236f) | GO:0008150~biological_process, | GO:0005575~cellular_component, | GO:0003674~molecular_function, |
| RMARP24049 | Rho guanine nucleotide exchange factor 10(arhgef10) | GO:0030036~actin cytoskeleton organization,GO:0051496~positive regulation of stress fiber assembly,GO:0090307~mitotic spindle assembly, | GO:0005737~cytoplasm,GO:0005813~centrosome, | GO:0005085~guanyl-nucleotide exchange factor activity, |
| RMARP24165 | Novel gene | N/A | N/A | N/A |
| RMARP24669 | transmembrane protein 117(TMEM117) | GO:0070059~intrinsic apoptotic signaling pathway in response to endoplasmic reticulum stress, | GO:0005783~endoplasmic reticulum,GO:0005886~plasma membrane, | N/A |
| RMARP25441 | dpy-19-like 4(dpy19l4) | N/A | GO:0005789~endoplasmic reticulum membrane,GO:0016020~membrane, | GO:0000030~mannosyltransferase activity,GO:0016757~glycosyltransferase activity, |
| RMARP25442 | frequently rearranged in advanced T-cell lymphomas 1(frat1) | N/A | GO:0005737~cytoplasm, | N/A |
| RMARP25458 | grainyhead like transcription factor 2(GRHL2) | GO:0003208~cardiac ventricle morphogenesis,GO:0006357~regulation of transcription by RNA polymerase II,GO:0007155~cell adhesion,GO:0007420~brain development,GO:0021915~neural tube development,GO:0030216~keratinocyte differentiation,GO:0035264~multicellular organism growth,GO:0042733~embryonic digit morphogenesis,GO:0043010~camera-type eye development,GO:0048701~embryonic cranial skeleton morphogenesis,GO:0060324~face development,GO:0060463~lung lobe morphogenesis,GO:0060487~lung epithelial cell differentiation,GO:0060672~epithelial cell morphogenesis involved in placental branching,GO:0061713~anterior neural tube closure,GO:0070830~bicellular tight junction assembly,GO:0090132~epithelium migration, | GO:0005634~nucleus,GO:0005654~nucleoplasm,GO:0005911~cell-cell junction, | GO:0000978~RNA polymerase II cis-regulatory region sequence-specific DNA binding,GO:0001161~intronic transcription regulatory region sequence-specific DNA binding,GO:0001228~DNA-binding transcription activator activity, RNA polymerase II-specific,GO:0003677~DNA binding,GO:0003700~DNA-binding transcription factor activity,GO:0031490~chromatin DNA binding,GO:0042802~identical protein binding,GO:0140297~DNA-binding transcription factor binding, |
| RMARP27692 | sterile alpha motif domain containing 12(SAMD12) | GO:0007169~cell surface receptor protein tyrosine kinase signaling pathway, | GO:0009898~cytoplasmic side of plasma membrane, | GO:0005515~protein binding, |
| RMARP28111 | Novel gene | N/A | N/A | N/A |
| RMARP28112 | eukaryotic translation initiation factor 3 subunit H(eif3h) | GO:0001732~formation of cytoplasmic translation initiation complex,GO:0002183~cytoplasmic translational initiation,GO:0006412~translation,GO:0006413~translational initiation, | GO:0005737~cytoplasm,GO:0005852~eukaryotic translation initiation factor 3 complex,GO:0016282~eukaryotic 43S preinitiation complex,GO:0033290~eukaryotic 48S preinitiation complex, | GO:0003743~translation initiation factor activity,GO:0008237~metallopeptidase activity, |
| RMARP30528 | calcium voltage-gated channel subunit alpha1 A(CACNA1A) | GO:0006816~calcium ion transport,GO:0007204~positive regulation of cytosolic calcium ion concentration,GO:0007268~chemical synaptic transmission,GO:0008219~cell death,GO:0050804~modulation of chemical synaptic transmission,GO:0070588~calcium ion transmembrane transport,GO:0098703~calcium ion import across plasma membrane,GO:1904645~response to amyloid-beta,GO:1904646~cellular response to amyloid-beta, | GO:0005634~nucleus,GO:0005737~cytoplasm,GO:0005886~plasma membrane,GO:0005891~voltage-gated calcium channel complex,GO:0016020~membrane,GO:0034702~monoatomic ion channel complex,GO:0042995~cell projection,GO:0043025~neuronal cell body,GO:0045202~synapse, | GO:0001540~amyloid-beta binding,GO:0005216~monoatomic ion channel activity,GO:0005245~voltage-gated calcium channel activity,GO:0005262~calcium channel activity,GO:0005509~calcium ion binding,GO:0005515~protein binding,GO:0008331~high voltage-gated calcium channel activity,GO:0019905~syntaxin binding,GO:0046872~metal ion binding, |
| RMARP31181 | arachidonate 15-lipoxygenase, type B(alox15b) | GO:0006629~lipid metabolic process,GO:0019369~arachidonate metabolic process,GO:0019372~lipoxygenase pathway,GO:0034440~lipid oxidation, | GO:0005737~cytoplasm, | GO:0005506~iron ion binding,GO:0016702~oxidoreductase activity, acting on single donors with incorporation of molecular oxygen, incorporation of two atoms of oxygen,GO:0016853~isomerase activity,GO:0046872~metal ion binding,GO:0051213~dioxygenase activity, |
| RMARP33700 | complement C3(c3) | GO:0006954~inflammatory response,GO:0006956~complement activation, | GO:0005576~extracellular region,GO:0005615~extracellular space, | GO:0004866~endopeptidase inhibitor activity, |
| RMARP33702 | Novel gene | N/A | N/A | N/A |

Table S9: Putative outliers detected by BayPass between AU_group1 and AU_Group2 in Australia. Gene ontology terms were assigned using DAVID.

| **Gene** | **Annotation from DAVID** | **GOTERM_BP_DIRECT** | **GOTERM_CC_DIRECT** | **GOTERM_MF_DIRECT** |
| --- | --- | --- | --- | --- |
| RMARP01566 | corticotropin releasing hormone binding protein(crhbp) | GO:0009755~hormone-mediated signaling pathway,GO:0051460~negative regulation of corticotropin secretion, | GO:0005576~extracellular region,GO:0005615~extracellular space, | GO:0051424~corticotropin-releasing hormone binding, |
| RMARP01567 | component of oligomeric golgi complex 2(Cog2) | GO:0000301~retrograde transport, vesicle recycling within Golgi,GO:0006891~intra-Golgi vesicle-mediated transport,GO:0007030~Golgi organization,GO:0015031~protein transport,GO:0070085~glycosylation, | GO:0000139~Golgi membrane,GO:0005794~Golgi apparatus,GO:0005795~Golgi stack,GO:0016020~membrane,GO:0017119~Golgi transport complex, | GO:0044877~protein-containing complex binding, |
| RMARP01665 | versican(VCAN) | GO:0007155~cell adhesion, | GO:0001750~photoreceptor outer segment,GO:0005576~extracellular region,GO:0033165~interphotoreceptor matrix, | GO:0005509~calcium ion binding,GO:0005540~hyaluronic acid binding,GO:0030246~carbohydrate binding, |
| RMARP03532 | cilia and flagella associated protein 73(cfap73) | GO:0006508~proteolysis,GO:0030198~extracellular matrix organization, | GO:0005576~extracellular region,GO:0005856~cytoskeleton,GO:0043232~intracellular membraneless organelle, | GO:0004222~metalloendopeptidase activity,GO:0046872~metal ion binding, |
| RMARP03618 | semaphorin 6A(sema6a) | GO:0001755~neural crest cell migration,GO:0007411~axon guidance,GO:0071526~semaphorin-plexin signaling pathway,GO:2001224~positive regulation of neuron migration, | GO:0005886~plasma membrane,GO:0016020~membrane, | GO:0030215~semaphorin receptor binding,GO:0045499~chemorepellent activity, |
| RMARP03926 | COMM domain containing 8(COMMD8) | N/A | GO:0005654~nucleoplasm,GO:0005829~cytosol, | N/A |
| RMARP03927 | ATPase, class V, type 10D(atp10d) | GO:0015914~phospholipid transport,GO:0045332~phospholipid translocation, | GO:0005886~plasma membrane,GO:0016020~membrane, | GO:0000287~magnesium ion binding,GO:0005524~ATP binding,GO:0016887~ATP hydrolysis activity,GO:0046872~metal ion binding,GO:0140326~ATPase-coupled intramembrane lipid transporter activity, |
| RMARP03928 | corin, serine peptidase(corin) | GO:0007186~G protein-coupled receptor signaling pathway,GO:0008217~regulation of blood pressure,GO:0016486~peptide hormone processing, | GO:0005886~plasma membrane,GO:0016020~membrane, | GO:0004252~serine-type endopeptidase activity,GO:0004930~G protein-coupled receptor activity,GO:0008236~serine-type peptidase activity, |
| RMARP03932 | nuclear transcription factor, X-box binding like 1(nfxl1) | GO:0006355~regulation of DNA-templated transcription, | GO:0005634~nucleus,GO:0016020~membrane, | GO:0000977~RNA polymerase II transcription regulatory region sequence-specific DNA binding,GO:0000981~DNA-binding transcription factor activity, RNA polymerase II-specific,GO:0003700~DNA-binding transcription factor activity,GO:0008270~zinc ion binding,GO:0046872~metal ion binding, |
| RMARP03934 | cyclic nucleotide gated channel alpha 1(cnga1) | GO:0006811~monoatomic ion transport,GO:0007601~visual perception,GO:0034220~monoatomic ion transmembrane transport,GO:0055085~transmembrane transport,GO:0098655~monoatomic cation transmembrane transport, | GO:0005886~plasma membrane,GO:0016020~membrane,GO:0017071~intracellular cyclic nucleotide activated cation channel complex, | GO:0005216~monoatomic ion channel activity,GO:0005221~intracellularly cyclic nucleotide-activated monoatomic cation channel activity,GO:0005222~intracellularly cAMP-activated cation channel activity,GO:0005223~intracellularly cGMP-activated cation channel activity,GO:0030552~cAMP binding,GO:0030553~cGMP binding,GO:0044877~protein-containing complex binding, |
| RMARP03956 | RAS like family 11 member B(rasl11b) | GO:0048382~mesendoderm development, | N/A | GO:0003924~GTPase activity,GO:0003925~G protein activity,GO:0005525~GTP binding,GO:0016787~hydrolase activity, |
| RMARP03957 | sec1 family domain containing 2(scfd2) | GO:0006886~intracellular protein transport,GO:0016192~vesicle-mediated transport, | N/A | N/A |
| RMARP03960 | factor interacting with PAPOLA and CPSF1(fip1l1) | GO:0006397~mRNA processing, | GO:0005634~nucleus,GO:0005847~mRNA cleavage and polyadenylation specificity factor complex, | N/A |
| RMARP03961 | choline/ethanolaminephosphotransferase 1(Cept1) | GO:0006646~phosphatidylethanolamine biosynthetic process,GO:0006656~phosphatidylcholine biosynthetic process,GO:0006657~CDP-choline pathway,GO:0008654~phospholipid biosynthetic process, | GO:0005789~endoplasmic reticulum membrane,GO:0005794~Golgi apparatus,GO:0016020~membrane,GO:0031965~nuclear membrane, | GO:0004142~diacylglycerol cholinephosphotransferase activity,GO:0004307~ethanolaminephosphotransferase activity,GO:0016780~phosphotransferase activity, for other substituted phosphate groups,GO:0046872~metal ion binding,GO:0047359~1-alkenyl-2-acylglycerol choline phosphotransferase activity, |
| RMARP03964 | GS homeobox 2(gsx2) | GO:0006355~regulation of DNA-templated transcription,GO:0048666~neuron development, | GO:0005634~nucleus, | GO:0000981~DNA-binding transcription factor activity, RNA polymerase II-specific,GO:0003677~DNA binding,GO:1990837~sequence-specific double-stranded DNA binding, |
| RMARP03972 | steroid 5 alpha-reductase 3(srd5a3) | GO:0006486~protein glycosylation,GO:0006488~dolichol-linked oligosaccharide biosynthetic process,GO:0006629~lipid metabolic process,GO:0016095~polyprenol catabolic process,GO:0019348~dolichol metabolic process,GO:0019408~dolichol biosynthetic process, | GO:0005783~endoplasmic reticulum,GO:0005789~endoplasmic reticulum membrane,GO:0012505~endomembrane system,GO:0016020~membrane, | GO:0016491~oxidoreductase activity,GO:0016627~oxidoreductase activity, acting on the CH-CH group of donors,GO:0016628~oxidoreductase activity, acting on the CH-CH group of donors, NAD or NADP as acceptor,GO:0047751~3-oxo-5alpha-steroid 4-dehydrogenase (NADP+) activity,GO:0102389~polyprenol reductase activity,GO:0160198~polyprenal reductase activity, |
| RMARP03973 | transmembrane protein 165(TMEM165) | GO:0006487~protein N-linked glycosylation,GO:0032468~Golgi calcium ion homeostasis,GO:0032472~Golgi calcium ion transport,GO:0035751~regulation of lysosomal lumen pH,GO:0070588~calcium ion transmembrane transport,GO:0071421~manganese ion transmembrane transport, | GO:0005765~lysosomal membrane,GO:0005794~Golgi apparatus,GO:0010008~endosome membrane,GO:0032588~trans-Golgi network membrane,GO:0033106~cis-Golgi network membrane, | GO:0005384~manganese ion transmembrane transporter activity,GO:0015085~calcium ion transmembrane transporter activity, |
| RMARP04147 | acidic nuclear phosphoprotein 32 family member E(ANP32E) | GO:0006325~chromatin organization,GO:0006457~protein folding,GO:0042981~regulation of apoptotic process, | GO:0000812~Swr1 complex,GO:0005634~nucleus,GO:0005737~cytoplasm,GO:0030672~synaptic vesicle membrane,GO:0031410~cytoplasmic vesicle,GO:0098839~postsynaptic density membrane,GO:0098895~postsynaptic endosome membrane,GO:0098978~glutamatergic synapse, | GO:0005515~protein binding,GO:0019212~phosphatase inhibitor activity,GO:0042393~histone binding,GO:0044183~protein folding chaperone,GO:0140713~histone chaperone activity, |
| RMARP04414 | hepatocyte growth factor-regulated tyrosine kinase substrate(HGS) | GO:0006622~protein targeting to lysosome,GO:0007165~signal transduction,GO:0008285~negative regulation of cell population proliferation,GO:0010324~membrane invagination,GO:0010628~positive regulation of gene expression,GO:0010642~negative regulation of platelet-derived growth factor receptor signaling pathway,GO:0015031~protein transport,GO:0016197~endosomal transport,GO:0016236~macroautophagy,GO:0016525~negative regulation of angiogenesis,GO:0030948~negative regulation of vascular endothelial growth factor receptor signaling pathway,GO:0031623~receptor internalization,GO:0032456~endocytic recycling,GO:0036258~multivesicular body assembly,GO:0042176~regulation of protein catabolic process,GO:0043328~protein transport to vacuole involved in ubiquitin-dependent protein catabolic process via the multivesicular body sorting pathway,GO:0043405~regulation of MAP kinase activity,GO:0046426~negative regulation of receptor signaling pathway via JAK-STAT,GO:0072657~protein localization to membrane,GO:0090148~membrane fission,GO:1903543~positive regulation of exosomal secretion, | GO:0005764~lysosome,GO:0005768~endosome,GO:0005769~early endosome,GO:0005829~cytosol,GO:0031901~early endosome membrane,GO:0032585~multivesicular body membrane,GO:0033565~ESCRT-0 complex,GO:0070062~extracellular exosome,GO:0097013~phagocytic vesicle lumen, | GO:0005515~protein binding,GO:0016301~kinase activity,GO:0019904~protein domain specific binding,GO:0035091~phosphatidylinositol binding,GO:0043130~ubiquitin binding,GO:0044389~ubiquitin-like protein ligase binding,GO:0046872~metal ion binding, |
| RMARP04415 | Novel gene | N/A | N/A | N/A |
| RMARP04416 | Coiled-coil serine rich protein 1 (ccser1) | N/A | N/A | N/A |
| RMARP05035 | zinc finger protein 737(ZNF737) | GO:0006355~regulation of DNA-templated transcription,GO:0006357~regulation of transcription by RNA polymerase II, | GO:0005634~nucleus, | GO:0000978~RNA polymerase II cis-regulatory region sequence-specific DNA binding,GO:0000981~DNA-binding transcription factor activity, RNA polymerase II-specific,GO:0046872~metal ion binding, |
| RMARP05039 | STRA6-like(Stra6l) | GO:0034633~retinol transport,GO:0051649~establishment of localization in cell,GO:0071939~vitamin A import into cell, | GO:0005886~plasma membrane, | GO:0005515~protein binding,GO:0034632~retinol transmembrane transporter activity,GO:0038023~signaling receptor activity, |
| RMARP05346 | Transmembrane serine protease 9(TMPRSS9) | GO:0006508~proteolysis,GO:0035821~modulation of process of another organism, | GO:0005576~extracellular region,GO:0016020~membrane, | GO:0004252~serine-type endopeptidase activity, |
| RMARP05960 | CR762475.1 (Clone-based gene) | N/A | N/A | N/A |
| RMARP05971 | SET domain containing 1B, histone lysine methyltransferase b (setd1bb) | N/A | N/A | N/A |
| RMARP05977 | lysine demethylase 2A(KDM2A) | GO:0006303~double-strand break repair via nonhomologous end joining,GO:0006325~chromatin organization,GO:0006357~regulation of transcription by RNA polymerase II,GO:0006482~protein demethylation,GO:0010944~negative regulation of transcription by competitive promoter binding,GO:0032922~circadian regulation of gene expression,GO:0042752~regulation of circadian rhythm, | GO:0005634~nucleus,GO:0005654~nucleoplasm, | GO:0003677~DNA binding,GO:0003712~transcription coregulator activity,GO:0005515~protein binding,GO:0008270~zinc ion binding,GO:0032452~histone demethylase activity,GO:0045322~unmethylated CpG binding,GO:0046872~metal ion binding,GO:0051213~dioxygenase activity,GO:0051864~histone H3K36 demethylase activity, |
| RMARP05977 | F-box and leucine rich repeat protein 19(FBXL19) | GO:0006338~chromatin remodeling,GO:0006357~regulation of transcription by RNA polymerase II,GO:0043161~proteasome-mediated ubiquitin-dependent protein catabolic process, | GO:0005634~nucleus,GO:0005737~cytoplasm,GO:0005829~cytosol,GO:0019005~SCF ubiquitin ligase complex, | GO:0003677~DNA binding,GO:0003712~transcription coregulator activity,GO:0005515~protein binding,GO:0008270~zinc ion binding,GO:0032452~histone demethylase activity,GO:0045322~unmethylated CpG binding,GO:0046872~metal ion binding,GO:1990756~ubiquitin-like ligase-substrate adaptor activity, |
| RMARP05978 | proline rich 13(PRR13) | N/A | GO:0005654~nucleoplasm,GO:0005829~cytosol, | GO:0005515~protein binding, |
| RMARP05979 | CD59b antigen(Cd59b) | GO:0001971~negative regulation of activation of membrane attack complex,GO:0042102~positive regulation of T cell proliferation,GO:0043066~negative regulation of apoptotic process,GO:0045916~negative regulation of complement activation,GO:1903660~negative regulation of complement-dependent cytotoxicity, | GO:0005615~extracellular space,GO:0005886~plasma membrane,GO:0009897~external side of plasma membrane,GO:0009986~cell surface,GO:0042383~sarcolemma,GO:0043218~compact myelin, | GO:0001848~complement binding, |
| RMARP06914 | KIAA0825(KIAA0825) | N/A | N/A | GO:0005515~protein binding, |
| RMARP06935 | calpastatin(cast) | N/A | GO:0005737~cytoplasm, | GO:0004869~cysteine-type endopeptidase inhibitor activity,GO:0010859~calcium-dependent cysteine-type endopeptidase inhibitor activity, |
| RMARP07043 | epidermal growth factor receptor pathway substrate 15-like 1(eps15l1) | GO:0006897~endocytosis,GO:0016197~endosomal transport, | GO:0005737~cytoplasm,GO:0005886~plasma membrane,GO:0030132~clathrin coat of coated pit, | GO:0005509~calcium ion binding,GO:0046872~metal ion binding, |
| RMARP07049 | LO018680.1 (Clone-based gene) | N/A | N/A | N/A |
| RMARP07050 | COPI coat complex subunit epsilon(cope) | GO:0006888~endoplasmic reticulum to Golgi vesicle-mediated transport,GO:0006890~retrograde vesicle-mediated transport, Golgi to endoplasmic reticulum,GO:0006891~intra-Golgi vesicle-mediated transport,GO:0015031~protein transport,GO:0016192~vesicle-mediated transport, | GO:0000139~Golgi membrane,GO:0005737~cytoplasm,GO:0005794~Golgi apparatus,GO:0016020~membrane,GO:0030126~COPI vesicle coat,GO:0030663~COPI-coated vesicle membrane,GO:0031410~cytoplasmic vesicle, | GO:0005198~structural molecule activity, |
| RMARP07072 | SURP and G-patch domain containing 1(SUGP1) | GO:0006396~RNA processing,GO:0006397~mRNA processing,GO:0008380~RNA splicing, | GO:0005634~nucleus,GO:0005654~nucleoplasm,GO:0005681~spliceosomal complex, | GO:0003723~RNA binding,GO:0005515~protein binding, |
| RMARP07088 | GTP binding protein 3, mitochondrial(gtpbp3) | GO:0002098~tRNA wobble uridine modification,GO:0006400~tRNA modification,GO:0008033~tRNA processing,GO:0030488~tRNA methylation, | GO:0005737~cytoplasm,GO:0005739~mitochondrion, | GO:0003924~GTPase activity,GO:0005525~GTP binding, |
| RMARP07325 | germ cell nuclear acidic peptidase(gcna) | GO:0006259~DNA metabolic process,GO:0006950~response to stress,GO:0051983~regulation of chromosome segregation,GO:0106300~protein-DNA covalent cross-linking repair,GO:0140014~mitotic nuclear division, | GO:0005634~nucleus,GO:0005694~chromosome,GO:0016605~PML body, | N/A |
| RMARP07327 | coiled-coil domain containing 124(ccdc124) | GO:0006366~transcription by RNA polymerase II, | GO:0005634~nucleus,GO:0005737~cytoplasm,GO:0005813~centrosome,GO:0005856~cytoskeleton,GO:0030496~midbody, | GO:0003713~transcription coactivator activity, |
| RMARP07538 | solute carrier family 10 member 7(SLC10A7) | GO:0006814~sodium ion transport,GO:0006874~intracellular calcium ion homeostasis,GO:0030210~heparin biosynthetic process,GO:0034436~glycoprotein transport,GO:0048193~Golgi vesicle transport,GO:0060348~bone development, | GO:0000139~Golgi membrane,GO:0005789~endoplasmic reticulum membrane,GO:0005797~Golgi medial cisterna,GO:0005801~cis-Golgi network,GO:0005802~trans-Golgi network,GO:0005886~plasma membrane, | GO:0015125~bile acid transmembrane transporter activity,GO:0015293~symporter activity, |
| RMARP07553 | nuclear receptor subfamily 3 group C member 2(NR3C2) | GO:0006357~regulation of transcription by RNA polymerase II,GO:0007165~signal transduction,GO:0030518~nuclear receptor-mediated steroid hormone signaling pathway,GO:1901224~positive regulation of non-canonical NF-kappaB signal transduction, | GO:0000785~chromatin,GO:0005634~nucleus,GO:0005654~nucleoplasm,GO:0005789~endoplasmic reticulum membrane,GO:0005829~cytosol,GO:0043235~receptor complex, | GO:0000981~DNA-binding transcription factor activity, RNA polymerase II-specific,GO:0003700~DNA-binding transcription factor activity,GO:0003707~nuclear steroid receptor activity,GO:0004879~nuclear receptor activity,GO:0005496~steroid binding,GO:0005515~protein binding,GO:0008270~zinc ion binding,GO:0017025~TBP-class protein binding,GO:0034056~estrogen response element binding,GO:0043565~sequence-specific DNA binding,GO:1990837~sequence-specific double-stranded DNA binding, |
| RMARP08637 | BX571665.1 (Clone-based gene) | N/A | N/A | N/A |
| RMARP08672 | NEDD4 binding protein 1(n4bp1) | GO:0031397~negative regulation of protein ubiquitination,GO:0032435~negative regulation of proteasomal ubiquitin-dependent protein catabolic process,GO:0045087~innate immune response, | GO:0005634~nucleus,GO:0005730~nucleolus,GO:0016605~PML body, | GO:0003723~RNA binding, |
| RMARP08673 | CYLD lysine 63 deubiquitinase(CYLD) | GO:0007346~regulation of mitotic cell cycle,GO:0010803~regulation of tumor necrosis factor-mediated signaling pathway,GO:0016055~Wnt signaling pathway,GO:0016579~protein deubiquitination,GO:0032088~negative regulation of NF-kappaB transcription factor activity,GO:0043369~CD4-positive or CD8-positive, alpha-beta T cell lineage commitment,GO:0045087~innate immune response,GO:0045577~regulation of B cell differentiation,GO:0045582~positive regulation of T cell differentiation,GO:0046329~negative regulation of JNK cascade,GO:0048872~homeostasis of number of cells,GO:0050728~negative regulation of inflammatory response,GO:0050862~positive regulation of T cell receptor signaling pathway,GO:0060544~regulation of necroptotic process,GO:0070266~necroptotic process,GO:0070507~regulation of microtubule cytoskeleton organization,GO:0070536~protein K63-linked deubiquitination,GO:0090090~negative regulation of canonical Wnt signaling pathway,GO:1901026~ripoptosome assembly involved in necroptotic process,GO:1901223~negative regulation of non-canonical NF-kappaB signal transduction,GO:1902017~regulation of cilium assembly,GO:1903753~negative regulation of p38MAPK cascade,GO:1903829~positive regulation of protein localization,GO:2000493~negative regulation of interleukin-18-mediated signaling pathway,GO:2001238~positive regulation of extrinsic apoptotic signaling pathway,GO:2001242~regulation of intrinsic apoptotic signaling pathway, | GO:0005813~centrosome,GO:0005819~spindle,GO:0005829~cytosol,GO:0005874~microtubule,GO:0005881~cytoplasmic microtubule,GO:0005886~plasma membrane,GO:0009898~cytoplasmic side of plasma membrane,GO:0030496~midbody,GO:0036064~ciliary basal body,GO:0042995~cell projection,GO:0048471~perinuclear region of cytoplasm,GO:0097542~ciliary tip, | GO:0004843~cysteine-type deubiquitinase activity,GO:0008270~zinc ion binding,GO:0019901~protein kinase binding,GO:0061578~K63-linked deubiquitinase activity,GO:0070064~proline-rich region binding,GO:1990380~K48-linked deubiquitinase activity, |
| RMARP08675 | receptor-interacting serine/threonine-protein kinase 2(LOC100490663) | GO:0006915~apoptotic process,GO:0006950~response to stress,GO:0007166~cell surface receptor signaling pathway,GO:0009893~positive regulation of metabolic process,GO:0031349~positive regulation of defense response,GO:0042981~regulation of apoptotic process,GO:0043123~positive regulation of canonical NF-kappaB signal transduction, | GO:0005737~cytoplasm, | GO:0000166~nucleotide binding,GO:0004672~protein kinase activity,GO:0004674~protein serine/threonine kinase activity,GO:0005524~ATP binding, |
| RMARP08676 | nuclear factor of activated T cells 4(nfatc4) | GO:0006355~regulation of DNA-templated transcription,GO:0033173~calcineurin-NFAT signaling cascade,GO:0045944~positive regulation of transcription by RNA polymerase II, | GO:0005634~nucleus,GO:0005667~transcription regulator complex,GO:0005737~cytoplasm, | GO:0000978~RNA polymerase II cis-regulatory region sequence-specific DNA binding,GO:0000981~DNA-binding transcription factor activity, RNA polymerase II-specific,GO:0003677~DNA binding,GO:0003700~DNA-binding transcription factor activity, |
| RMARP09796 | Mitochondrial import receptor subunit TOM5 homolog (A0A8C6Y5E4_NAJNA) | N/A | GO:0005742~mitochondrial outer membrane translocase complex, | N/A |
| RMARP09797 | Rab geranylgeranyltransferase subunit alpha(rabggta) | GO:0006888~endoplasmic reticulum to Golgi vesicle-mediated transport,GO:0097354~prenylation, | GO:0005737~cytoplasm,GO:0005968~Rab-protein geranylgeranyltransferase complex, | GO:0004659~prenyltransferase activity,GO:0004663~Rab geranylgeranyltransferase activity,GO:0008270~zinc ion binding, |
| RMARP09919 | tyrosine 3-monooxygenase/tryptophan 5-monooxygenase activation protein eta(ywhah) | GO:0001702~gastrulation with mouth forming second,GO:0003401~axis elongation,GO:0007165~signal transduction,GO:0008104~protein localization, | GO:0005737~cytoplasm, | GO:0004497~monooxygenase activity, |
| RMARP09946 | calcineurin binding protein 1(CABIN1) | GO:0006325~chromatin organization, | GO:0005634~nucleus, | GO:0005515~protein binding,GO:0031491~nucleosome binding, |
| RMARP10956 | ATPase phospholipid transporting 8A1(ATP8A1) | GO:0015914~phospholipid transport,GO:0045332~phospholipid translocation, | GO:0005886~plasma membrane,GO:0016020~membrane, | GO:0000287~magnesium ion binding,GO:0005524~ATP binding,GO:0016887~ATP hydrolysis activity,GO:0140326~ATPase-coupled intramembrane lipid transporter activity, |
| RMARP10972 | LIM and calponin homology domains 1(limch1) | GO:0031032~actomyosin structure organization, | N/A | GO:0003779~actin binding,GO:0046872~metal ion binding, |
| RMARP11019 | Sgcz | N/A | N/A | N/A |
| RMARP11142 | N-deacetylase/N-sulfotransferase (heparan glucosaminyl) 3(ndst3) | GO:0015012~heparan sulfate proteoglycan biosynthetic process,GO:0015014~heparan sulfate proteoglycan biosynthetic process, polysaccharide chain biosynthetic process,GO:0030210~heparin biosynthetic process, | GO:0000139~Golgi membrane,GO:0005794~Golgi apparatus,GO:0016020~membrane, | GO:0015016~[heparan sulfate]-glucosamine N-sulfotransferase activity,GO:0016787~hydrolase activity,GO:0019213~deacetylase activity, |
| RMARP11506 | Novel gene | N/A | N/A | N/A |
| RMARP11515 | F-box protein 8(fbxo8) | GO:0006511~ubiquitin-dependent protein catabolic process,GO:0032012~regulation of ARF protein signal transduction, | GO:0000151~ubiquitin ligase complex, | GO:0005085~guanyl-nucleotide exchange factor activity,GO:0005515~protein binding, |
| RMARP11534 | REST corepressor 3(RCOR3) | GO:0006357~regulation of transcription by RNA polymerase II,GO:0045892~negative regulation of DNA-templated transcription, | GO:0000118~histone deacetylase complex,GO:0005634~nucleus,GO:0005654~nucleoplasm,GO:0005667~transcription regulator complex,GO:0005829~cytosol, | GO:0003714~transcription corepressor activity,GO:0005515~protein binding, |
| RMARP11535 | Uncharacterised gene | N/A | N/A | N/A |
| RMARP11536 | erythrocyte membrane protein band 4.1 like 4A(epb41l4a) | GO:0031032~actomyosin structure organization, | GO:0005856~cytoskeleton, | GO:0008092~cytoskeletal protein binding, |
| RMARP11539 | enkurin domain containing 1 (enkd1) | N/A | N/A | N/A |
| RMARP12138 | Rasef | N/A | N/A | N/A |
| RMARP12141 | idnK, gluconokinase homolog (E. coli)(idnk) | GO:0005975~carbohydrate metabolic process,GO:0046177~D-gluconate catabolic process, | N/A | GO:0005524~ATP binding,GO:0016301~kinase activity,GO:0046316~gluconokinase activity, |
| RMARP12376 | Coiled-coil domain containing 171 (ccdc171) | N/A | N/A | N/A |
| RMARP12377 | hydroxysteroid 17-beta dehydrogenase 4(HSD17B4) | GO:0000038~very long-chain fatty acid metabolic process,GO:0001649~osteoblast differentiation,GO:0006635~fatty acid beta-oxidation,GO:0008209~androgen metabolic process,GO:0008210~estrogen metabolic process,GO:0036111~very long-chain fatty-acyl-CoA metabolic process,GO:0036112~medium-chain fatty-acyl-CoA metabolic process,GO:0060009~Sertoli cell development, | GO:0005777~peroxisome,GO:0005778~peroxisomal membrane,GO:0005782~peroxisomal matrix,GO:0005829~cytosol,GO:0016020~membrane,GO:0043231~intracellular membrane-bounded organelle, | GO:0003857~3-hydroxyacyl-CoA dehydrogenase activity,GO:0004300~enoyl-CoA hydratase activity,GO:0004303~estradiol 17-beta-dehydrogenase [NAD(P)+] activity,GO:0016491~oxidoreductase activity,GO:0016853~isomerase activity,GO:0018812~3-hydroxyacyl-CoA dehydratase activity,GO:0033989~3alpha,7alpha,12alpha-trihydroxy-5beta-cholest-24-enoyl-CoA hydratase activity,GO:0042803~protein homodimerization activity,GO:0044594~17-beta-hydroxysteroid dehydrogenase (NAD+) activity,GO:0106386~(3R)-hydroxyacyl-CoA dehydrogenase (NAD+) activity, |
| RMARP12619 | dachsous cadherin-related 2(DCHS2) | GO:0007156~homophilic cell adhesion via plasma membrane adhesion molecules, | GO:0005886~plasma membrane,GO:0005911~cell-cell junction,GO:0016342~catenin complex, | GO:0005509~calcium ion binding,GO:0045296~cadherin binding, |
| RMARP13138 | microtubule associated serine/threonine kinase family member 4(MAST4) | GO:0006338~chromatin remodeling,GO:0007010~cytoskeleton organization,GO:0035556~intracellular signal transduction, | GO:0005737~cytoplasm, | GO:0000287~magnesium ion binding,GO:0004674~protein serine/threonine kinase activity,GO:0004676~3-phosphoinositide-dependent protein kinase activity,GO:0004677~DNA-dependent protein kinase activity,GO:0004679~AMP-activated protein kinase activity,GO:0004694~eukaryotic translation initiation factor 2alpha kinase activity,GO:0004711~ribosomal protein S6 kinase activity,GO:0005524~ATP binding,GO:0035175~histone H3S10 kinase activity,GO:0035402~histone H3T11 kinase activity,GO:0035403~histone H3T6 kinase activity,GO:0035979~histone H2AXS139 kinase activity,GO:0044022~histone H3S28 kinase activity,GO:0044023~histone H4S1 kinase activity,GO:0044024~histone H2AS1 kinase activity,GO:0044025~histone H2BS14 kinase activity,GO:0072354~histone H3T3 kinase activity,GO:0072371~histone H2AS121 kinase activity,GO:0072518~Rho-dependent protein serine/threonine kinase activity,GO:0106310~protein serine kinase activity,GO:0140823~histone H2BS36 kinase activity,GO:0140855~histone H3S57 kinase activity,GO:0140857~histone H3T45 kinase activity,GO:1990244~histone H2AT120 kinase activity, |
| RMARP13406 | seizure related 6 homolog-like(sez6l) | GO:0060074~synapse maturation,GO:0090036~regulation of protein kinase C signaling, | GO:0005783~endoplasmic reticulum,GO:0016020~membrane,GO:0043025~neuronal cell body, | N/A |
| RMARP13607 | Coiled-coil domain containing 60 (ccdc60) | N/A | N/A | N/A |
| RMARP13608 | sushi domain containing 2(susd2) | N/A | GO:0005615~extracellular space,GO:0016020~membrane, | N/A |
| RMARP13621 | si:ch73-366l1.5(si:ch73-366l1.5) | N/A | GO:0016020~membrane, | N/A |
| RMARP13623 | oxysterol binding protein(OSBP) | GO:0006686~sphingomyelin biosynthetic process,GO:0006699~bile acid biosynthetic process,GO:0006869~lipid transport,GO:0015914~phospholipid transport,GO:0015918~sterol transport,GO:0032367~intracellular cholesterol transport,GO:0035627~ceramide transport,GO:0035774~positive regulation of insulin secretion involved in cellular response to glucose stimulus,GO:0042531~positive regulation of tyrosine phosphorylation of STAT protein,GO:1904411~positive regulation of secretory granule organization, | GO:0000139~Golgi membrane,GO:0005654~nucleoplasm,GO:0005730~nucleolus,GO:0005737~cytoplasm,GO:0005789~endoplasmic reticulum membrane,GO:0005794~Golgi apparatus,GO:0005802~trans-Golgi network,GO:0005829~cytosol,GO:0005886~plasma membrane,GO:0016020~membrane,GO:0030054~cell junction,GO:0048471~perinuclear region of cytoplasm,GO:0097038~perinuclear endoplasmic reticulum, | GO:0005515~protein binding,GO:0008142~oxysterol binding,GO:0008289~lipid binding,GO:0019904~protein domain specific binding,GO:0032934~sterol binding,GO:0070273~phosphatidylinositol-4-phosphate binding,GO:0120015~sterol transfer activity, |
| RMARP14212 | Leucine rich repeat containing 74B (lrrc74b) | N/A | N/A | N/A |
| RMARP14213 | HscB mitochondrial iron-sulfur cluster co-chaperone(hscb) | GO:0044571~[2Fe-2S] cluster assembly,GO:0051259~protein complex oligomerization, | GO:0005739~mitochondrion, | GO:0001671~ATPase activator activity,GO:0051087~protein-folding chaperone binding, |
| RMARP14812 | SH3-domain GRB2-like 2(sh3gl2) | GO:0006897~endocytosis,GO:0016191~synaptic vesicle uncoating, | GO:0005737~cytoplasm,GO:0005769~early endosome,GO:0016020~membrane,GO:0098793~presynapse,GO:0098978~glutamatergic synapse, | N/A |
| RMARP14813 | Tachykinin precursor 1(TAC1) | GO:0007217~tachykinin receptor signaling pathway,GO:0007218~neuropeptide signaling pathway, | GO:0005576~extracellular region, | GO:0003676~nucleic acid binding, |
| RMARP14824 | HAUS augmin like complex subunit 6(haus6) | GO:0000226~microtubule cytoskeleton organization,GO:0051225~spindle assembly, | GO:0070652~HAUS complex,GO:1990498~mitotic spindle microtubule, | GO:0008017~microtubule binding, |
| RMARP14825 | perilipin 2(plin2) | GO:0010890~positive regulation of sequestering of triglyceride,GO:0019915~lipid storage, | GO:0005811~lipid droplet,GO:0005829~cytosol, | N/A |
| RMARP14831 | alkaline ceramidase 2(acer2) | GO:0006665~sphingolipid metabolic process,GO:0006672~ceramide metabolic process,GO:0046512~sphingosine biosynthetic process,GO:0046514~ceramide catabolic process, | GO:0000139~Golgi membrane,GO:0016020~membrane, | GO:0016787~hydrolase activity,GO:0016811~hydrolase activity, acting on carbon-nitrogen (but not peptide) bonds, in linear amides,GO:0017040~N-acylsphingosine amidohydrolase activity, |
| RMARP14837 | focadhesin(focad) | GO:0060147~regulation of post-transcriptional gene silencing, | N/A | N/A |
| RMARP14840 | 3-hydroxyacyl-CoA dehydratase 4(hacd4) | GO:0006633~fatty acid biosynthetic process,GO:0030148~sphingolipid biosynthetic process,GO:0030497~fatty acid elongation,GO:0042761~very long-chain fatty acid biosynthetic process, | GO:0005783~endoplasmic reticulum,GO:0005789~endoplasmic reticulum membrane,GO:0016020~membrane, | GO:0016829~lyase activity,GO:0018812~3-hydroxyacyl-CoA dehydratase activity,GO:0102158~very-long-chain (3R)-3-hydroxyacyl-CoA dehydratase activity, |
| RMARP14855 | Uncharacterised gene | N/A | N/A | N/A |
| RMARP14963 | bone morphogenetic protein receptor type 1B(bmpr1b) | GO:0002063~chondrocyte development,GO:0007178~cell surface receptor protein serine/threonine kinase signaling pathway,GO:0009953~dorsal/ventral pattern formation,GO:0030154~cell differentiation,GO:0030166~proteoglycan biosynthetic process,GO:0030509~BMP signaling pathway,GO:0060350~endochondral bone morphogenesis,GO:0061036~positive regulation of cartilage development,GO:0071363~cellular response to growth factor stimulus,GO:1902731~negative regulation of chondrocyte proliferation, | GO:0005886~plasma membrane,GO:0016020~membrane,GO:0043235~receptor complex, | GO:0004674~protein serine/threonine kinase activity,GO:0004675~transmembrane receptor protein serine/threonine kinase activity,GO:0005025~transforming growth factor beta receptor activity, type I,GO:0005026~transforming growth factor beta receptor activity, type II,GO:0005524~ATP binding,GO:0016361~activin receptor activity, type I,GO:0016362~activin receptor activity, type II,GO:0070123~transforming growth factor beta receptor activity, type III,GO:0098821~BMP receptor activity, |
| RMARP14964 | PDZ and LIM domain 7 (PDLIM7) | N/A | N/A | N/A |
| RMARP15062 | Solute carrier organic anion transporter family member (A0A803SWP3_ANOCA) | GO:0006811~monoatomic ion transport,GO:0055085~transmembrane transport, | GO:0005886~plasma membrane, | N/A |
| RMARP15063 | negative regulator of P-body association(Nbdy) | GO:0000956~nuclear-transcribed mRNA catabolic process,GO:0010607~negative regulation of cytoplasmic mRNA processing body assembly, | GO:0000932~P-body, | N/A |
| RMARP15064 | Novel gene | N/A | N/A | N/A |
| RMARP15068 | diphosphoinositol pentakisphosphate kinase 2(ppip5k2) | GO:0006020~inositol metabolic process,GO:0032958~inositol phosphate biosynthetic process, | GO:0005737~cytoplasm,GO:0005829~cytosol,GO:0016020~membrane, | GO:0000827~inositol-1,3,4,5,6-pentakisphosphate kinase activity,GO:0000828~inositol hexakisphosphate kinase activity,GO:0000829~diphosphoinositol pentakisphosphate kinase activity,GO:0000832~inositol hexakisphosphate 5-kinase activity,GO:0005524~ATP binding,GO:0016301~kinase activity,GO:0033857~5-diphosphoinositol pentakisphosphate 1-kinase activity,GO:0052723~inositol hexakisphosphate 1-kinase activity,GO:0052724~inositol hexakisphosphate 3-kinase activity, |
| RMARP15122 | PR domain 5(prdm5) | GO:0006357~regulation of transcription by RNA polymerase II,GO:0032259~methylation, | GO:0005634~nucleus,GO:0005694~chromosome, | GO:0000976~transcription cis-regulatory region binding,GO:0008168~methyltransferase activity,GO:0016740~transferase activity,GO:0043035~chromatin insulator sequence binding,GO:0046872~metal ion binding, |
| RMARP15132 | cytochrome P450 family 2 subfamily U member 1(cyp2u1) | GO:0006082~organic acid metabolic process,GO:0006805~xenobiotic metabolic process, | GO:0005737~cytoplasm,GO:0016020~membrane,GO:0043231~intracellular membrane-bounded organelle, | GO:0004497~monooxygenase activity,GO:0005506~iron ion binding,GO:0008395~steroid hydroxylase activity,GO:0016712~oxidoreductase activity, acting on paired donors, with incorporation or reduction of molecular oxygen, reduced flavin or flavoprotein as one donor, and incorporation of one atom of oxygen,GO:0020037~heme binding,GO:0046872~metal ion binding, |
| RMARP15132 | vitamin D 25-hydroxylase(LOC100556624) | GO:0006082~organic acid metabolic process,GO:0006805~xenobiotic metabolic process, | GO:0005737~cytoplasm,GO:0016020~membrane,GO:0043231~intracellular membrane-bounded organelle, | GO:0005506~iron ion binding,GO:0008395~steroid hydroxylase activity,GO:0016712~oxidoreductase activity, acting on paired donors, with incorporation or reduction of molecular oxygen, reduced flavin or flavoprotein as one donor, and incorporation of one atom of oxygen,GO:0020037~heme binding,GO:0030343~vitamin D3 25-hydroxylase activity,GO:0042803~protein homodimerization activity,GO:1902271~D3 vitamins binding, |
| RMARP15153 | phospholipase A2 group IIC(PLA2G2C) | GO:0006633~fatty acid biosynthetic process,GO:0006644~phospholipid metabolic process,GO:0016042~lipid catabolic process,GO:0046470~phosphatidylcholine metabolic process,GO:0046471~phosphatidylglycerol metabolic process,GO:0048146~positive regulation of fibroblast proliferation,GO:0050482~arachidonate secretion, | GO:0005576~extracellular region, | GO:0004623~phospholipase A2 activity,GO:0005102~signaling receptor binding,GO:0005509~calcium ion binding,GO:0005543~phospholipid binding,GO:0047498~calcium-dependent phospholipase A2 activity, |
| RMARP15167 | pleckstrin and Sec7 domain containing 3(psd3) | GO:0032012~regulation of ARF protein signal transduction, | GO:0005886~plasma membrane,GO:0032587~ruffle membrane, | GO:0005085~guanyl-nucleotide exchange factor activity,GO:0005543~phospholipid binding, |
| RMARP16239 | palladin, cytoskeletal associated protein(PALLD) | GO:0003334~keratinocyte development,GO:0003382~epithelial cell morphogenesis,GO:0007010~cytoskeleton organization,GO:0007156~homophilic cell adhesion via plasma membrane adhesion molecules,GO:0007411~axon guidance,GO:0016477~cell migration,GO:0030036~actin cytoskeleton organization,GO:0030154~cell differentiation,GO:0070593~dendrite self-avoidance, | GO:0001725~stress fiber,GO:0001726~ruffle,GO:0002102~podosome,GO:0005634~nucleus,GO:0005739~mitochondrion,GO:0005829~cytosol,GO:0005884~actin filament,GO:0005886~plasma membrane,GO:0005925~focal adhesion,GO:0015629~actin cytoskeleton,GO:0030018~Z disc,GO:0030027~lamellipodium,GO:0030424~axon,GO:0030426~growth cone,GO:0060076~excitatory synapse, | GO:0003779~actin binding,GO:0004672~protein kinase activity,GO:0005515~protein binding,GO:0051371~muscle alpha-actinin binding,GO:0098632~cell-cell adhesion mediator activity, |
| RMARP16242 | SH3 domain containing ring finger 1(sh3rf1) | GO:0001764~neuron migration,GO:0016567~protein ubiquitination,GO:0032436~positive regulation of proteasomal ubiquitin-dependent protein catabolic process,GO:0043066~negative regulation of apoptotic process,GO:0043370~regulation of CD4-positive, alpha-beta T cell differentiation,GO:0046330~positive regulation of JNK cascade,GO:0051865~protein autoubiquitination,GO:2000564~regulation of CD8-positive, alpha-beta T cell proliferation, | GO:0005737~cytoplasm,GO:0005794~Golgi apparatus,GO:0005829~cytosol,GO:0030027~lamellipodium,GO:0042995~cell projection,GO:0048471~perinuclear region of cytoplasm, | GO:0005078~MAP-kinase scaffold activity,GO:0016740~transferase activity,GO:0046872~metal ion binding,GO:0061630~ubiquitin protein ligase activity, |
| RMARP16270 | RNA polymerase I subunit E(POLR1E) | GO:0006351~DNA-templated transcription, | GO:0000428~DNA-directed RNA polymerase complex,GO:0005730~nucleolus, | GO:0003677~DNA binding, |
| RMARP16271 | F-box protein 10(fbxo10) | GO:0006511~ubiquitin-dependent protein catabolic process,GO:0042981~regulation of apoptotic process, | N/A | N/A |
| RMARP16497 | Sperm tail PG-rich repeat containing 2 (STPG2) | N/A | N/A | N/A |
| RMARP16671 | DNA replication regulator and spliceosomal factor(SMU1) | GO:0000381~regulation of alternative mRNA splicing, via spliceosome,GO:0000398~mRNA splicing, via spliceosome,GO:0008380~RNA splicing, | GO:0005634~nucleus,GO:0005737~cytoplasm,GO:0016607~nuclear speck,GO:0071005~U2-type precatalytic spliceosome,GO:0071011~precatalytic spliceosome, | GO:0005515~protein binding, |
| RMARP16682 | major facilitator superfamily domain containing 12(mfsd12) | GO:0008643~carbohydrate transport,GO:0043474~pigment metabolic process involved in pigmentation,GO:0048021~regulation of melanin biosynthetic process,GO:1903712~cysteine transmembrane transport, | GO:0005886~plasma membrane,GO:0016020~membrane, | GO:0015293~symporter activity,GO:0033229~cysteine transmembrane transporter activity, |
| RMARP16683 | haptoglobin-related protein(HPR) | GO:0002376~immune system process,GO:0006508~proteolysis,GO:0006952~defense response,GO:0006953~acute-phase response,GO:0031638~zymogen activation,GO:0042542~response to hydrogen peroxide,GO:0042742~defense response to bacterium,GO:0051354~negative regulation of oxidoreductase activity,GO:2000296~negative regulation of hydrogen peroxide catabolic process, | GO:0005576~extracellular region,GO:0005615~extracellular space,GO:0031838~haptoglobin-hemoglobin complex,GO:0034366~spherical high-density lipoprotein particle,GO:0035580~specific granule lumen,GO:0070062~extracellular exosome,GO:0071682~endocytic vesicle lumen,GO:0072562~blood microparticle,GO:1904724~tertiary granule lumen, | GO:0004252~serine-type endopeptidase activity,GO:0016209~antioxidant activity,GO:0030492~hemoglobin binding, |
| RMARP17385 | olfactory receptor 11L1(LOC490585) | GO:0007186~G protein-coupled receptor signaling pathway,GO:0050911~detection of chemical stimulus involved in sensory perception of smell, | GO:0005886~plasma membrane,GO:0016020~membrane, | GO:0004930~G protein-coupled receptor activity,GO:0004984~olfactory receptor activity, |
| RMARP17533 | dynein, axonemal, heavy chain 6(dnah6) | GO:0007018~microtubule-based movement, | GO:0005737~cytoplasm,GO:0005856~cytoskeleton,GO:0005874~microtubule,GO:0005929~cilium,GO:0005930~axoneme,GO:0030286~dynein complex, | GO:0005524~ATP binding,GO:0008569~minus-end-directed microtubule motor activity,GO:0016887~ATP hydrolysis activity,GO:0045505~dynein intermediate chain binding,GO:0051959~dynein light intermediate chain binding, |
| RMARP17534 | protein arginine methyltransferase 3(PRMT3) | GO:0006338~chromatin remodeling,GO:0006355~regulation of DNA-templated transcription,GO:0031397~negative regulation of protein ubiquitination,GO:0032259~methylation,GO:1900053~negative regulation of retinoic acid biosynthetic process, | GO:0005634~nucleus,GO:0005737~cytoplasm,GO:0005829~cytosol,GO:0016020~membrane, | GO:0005515~protein binding,GO:0008168~methyltransferase activity,GO:0016274~protein-arginine N-methyltransferase activity,GO:0035241~protein-arginine omega-N monomethyltransferase activity,GO:0035242~protein-arginine omega-N asymmetric methyltransferase activity,GO:0042054~histone methyltransferase activity,GO:0043022~ribosome binding,GO:0046872~metal ion binding, |
| RMARP17536 | annexin A3(anxa3) | N/A | N/A | GO:0004859~phospholipase inhibitor activity,GO:0005509~calcium ion binding,GO:0005544~calcium-dependent phospholipid binding, |
| RMARP17587 | AFG2 AAA ATPase homolog A(afg2a) | GO:0042273~ribosomal large subunit biogenesis, | GO:0005737~cytoplasm, | GO:0005524~ATP binding,GO:0016887~ATP hydrolysis activity, |
| RMARP17595 | bridge-like lipid transfer protein family member 1(BLTP1) | GO:0001558~regulation of cell growth,GO:0006909~phagocytosis,GO:0015914~phospholipid transport,GO:0016197~endosomal transport,GO:0030856~regulation of epithelial cell differentiation,GO:0032456~endocytic recycling,GO:0048488~synaptic vesicle endocytosis,GO:0120009~intermembrane lipid transfer, | GO:0005634~nucleus,GO:0005789~endoplasmic reticulum membrane,GO:0005886~plasma membrane,GO:0016020~membrane,GO:0031966~mitochondrial membrane,GO:0098793~presynapse,GO:0140268~endoplasmic reticulum-plasma membrane contact site, | GO:0005515~protein binding,GO:1904121~phosphatidylethanolamine transfer activity, |
| RMARP18288 | dimethylglycine dehydrogenase(dmgdh) | N/A | GO:0005739~mitochondrion, | N/A |
| RMARP18292 | homer scaffold protein 1(homer1) | GO:0006874~intracellular calcium ion homeostasis,GO:0007216~G protein-coupled glutamate receptor signaling pathway,GO:0061061~muscle structure development,GO:2001256~regulation of store-operated calcium entry, | GO:0005737~cytoplasm,GO:0005886~plasma membrane,GO:0014069~postsynaptic density,GO:0030425~dendrite,GO:0045202~synapse, | GO:0035256~G protein-coupled glutamate receptor binding, |
| RMARP18320 | SWI/SNF related BAF chromatin remodeling complex subunit ATPase 4(SMARCA4) | GO:0000122~negative regulation of transcription by RNA polymerase II,GO:0001188~RNA polymerase I preinitiation complex assembly,GO:0003407~neural retina development,GO:0006337~nucleosome disassembly,GO:0006338~chromatin remodeling,GO:0006355~regulation of DNA-templated transcription,GO:0006357~regulation of transcription by RNA polymerase II,GO:0007399~nervous system development,GO:0008284~positive regulation of cell population proliferation,GO:0030071~regulation of mitotic metaphase/anaphase transition,GO:0030177~positive regulation of Wnt signaling pathway,GO:0030308~negative regulation of cell growth,GO:0031507~heterochromatin formation,GO:0043923~positive regulation by host of viral transcription,GO:0045582~positive regulation of T cell differentiation,GO:0045596~negative regulation of cell differentiation,GO:0045597~positive regulation of cell differentiation,GO:0045663~positive regulation of myoblast differentiation,GO:0045815~transcription initiation-coupled chromatin remodeling,GO:0045892~negative regulation of DNA-templated transcription,GO:0045893~positive regulation of DNA-templated transcription,GO:0045944~positive regulation of transcription by RNA polymerase II,GO:0051091~positive regulation of DNA-binding transcription factor activity,GO:0060766~negative regulation of androgen receptor signaling pathway,GO:0070316~regulation of G0 to G1 transition,GO:0120162~positive regulation of cold-induced thermogenesis,GO:1901838~positive regulation of transcription of nucleolar large rRNA by RNA polymerase I,GO:1902459~positive regulation of stem cell population maintenance,GO:1902661~positive regulation of glucose mediated signaling pathway,GO:1902895~positive regulation of miRNA transcription,GO:2000045~regulation of G1/S transition of mitotic cell cycle,GO:2000781~positive regulation of double-strand break repair,GO:2000819~regulation of nucleotide-excision repair, | GO:0000776~kinetochore,GO:0000785~chromatin,GO:0001650~fibrillar center,GO:0005615~extracellular space,GO:0005634~nucleus,GO:0005654~nucleoplasm,GO:0005694~chromosome,GO:0005730~nucleolus,GO:0016020~membrane,GO:0016363~nuclear matrix,GO:0016514~SWI/SNF complex,GO:0016586~RSC-type complex,GO:0032991~protein-containing complex,GO:0071564~npBAF complex,GO:0071565~nBAF complex,GO:0140092~bBAF complex,GO:0140288~GBAF complex, | GO:0001164~RNA polymerase I core promoter sequence-specific DNA binding,GO:0001221~transcription coregulator binding,GO:0002039~p53 binding,GO:0003677~DNA binding,GO:0003682~chromatin binding,GO:0003713~transcription coactivator activity,GO:0003714~transcription corepressor activity,GO:0003723~RNA binding,GO:0004386~helicase activity,GO:0005515~protein binding,GO:0005524~ATP binding,GO:0008094~ATP-dependent activity, acting on DNA,GO:0016787~hydrolase activity,GO:0030957~Tat protein binding,GO:0031492~nucleosomal DNA binding,GO:0042393~histone binding,GO:0050681~nuclear androgen receptor binding,GO:0070182~DNA polymerase binding,GO:0140658~ATP-dependent chromatin remodeler activity,GO:0140750~nucleosome array spacer activity, |
| RMARP19202 | SDS3 homolog, SIN3A corepressor complex component(suds3) | GO:0000122~negative regulation of transcription by RNA polymerase II,GO:0010468~regulation of gene expression, | GO:0005634~nucleus,GO:0005654~nucleoplasm,GO:0070822~Sin3-type complex, | GO:0042826~histone deacetylase binding, |
| RMARP19241 | Novel gene | N/A | N/A | N/A |
| RMARP19528 | checkpoint with forkhead and ring finger domains(chfr) | GO:0000209~protein polyubiquitination,GO:0006511~ubiquitin-dependent protein catabolic process,GO:0007093~mitotic cell cycle checkpoint signaling,GO:0016567~protein ubiquitination,GO:0031398~positive regulation of protein ubiquitination,GO:0031648~protein destabilization,GO:0032436~positive regulation of proteasomal ubiquitin-dependent protein catabolic process,GO:0044779~meiotic spindle checkpoint signaling,GO:0044818~mitotic G2/M transition checkpoint, | GO:0005634~nucleus,GO:0016605~PML body, | GO:0000166~nucleotide binding,GO:0004842~ubiquitin-protein transferase activity,GO:0005515~protein binding,GO:0046872~metal ion binding,GO:0061630~ubiquitin protein ligase activity, |
| RMARP20117 | Sorcs2 | N/A | N/A | N/A |
| RMARP20188 | SH2B adaptor protein 3(SH2B3) | GO:0001780~neutrophil homeostasis,GO:0008285~negative regulation of cell population proliferation,GO:0035162~embryonic hemopoiesis,GO:0035556~intracellular signal transduction,GO:0035702~monocyte homeostasis,GO:0035855~megakaryocyte development,GO:0036016~cellular response to interleukin-3,GO:0038163~thrombopoietin-mediated signaling pathway,GO:0043409~negative regulation of MAPK cascade,GO:0046426~negative regulation of receptor signaling pathway via JAK-STAT,GO:0048821~erythrocyte development,GO:0051898~negative regulation of phosphatidylinositol 3-kinase/protein kinase B signal transduction,GO:0060218~hematopoietic stem cell differentiation,GO:0070100~negative regulation of chemokine-mediated signaling pathway,GO:0090331~negative regulation of platelet aggregation,GO:1900235~negative regulation of Kit signaling pathway, | GO:0005886~plasma membrane, | GO:0005068~transmembrane receptor protein tyrosine kinase adaptor activity,GO:0005173~stem cell factor receptor binding,GO:0030159~signaling receptor complex adaptor activity,GO:1990782~protein tyrosine kinase binding, |
| RMARP20945 | nephronectin(npnt) | GO:0007155~cell adhesion,GO:0030154~cell differentiation, | GO:0005576~extracellular region,GO:0016020~membrane, | GO:0005509~calcium ion binding, |
| RMARP20947 | glutathione S-transferase, C-terminal domain containing(gstcd) | N/A | GO:0005737~cytoplasm, | N/A |
| RMARP21320 | FP085421.1 (Clone-based gene) | N/A | N/A | N/A |
| RMARP21568 | FAT atypical cadherin 4(fat4) | GO:0007155~cell adhesion,GO:0007156~homophilic cell adhesion via plasma membrane adhesion molecules, | GO:0005886~plasma membrane, | GO:0005509~calcium ion binding, |
| RMARP22311 | nitric oxide synthase 1(nos1) | GO:0001889~liver development,GO:0006809~nitric oxide biosynthetic process,GO:0007210~serotonin receptor signaling pathway,GO:0007263~nitric oxide mediated signal transduction,GO:0021517~ventral spinal cord development,GO:0048668~collateral sprouting,GO:0060218~hematopoietic stem cell differentiation, | GO:0042383~sarcolemma,GO:0043197~dendritic spine, | GO:0004517~nitric-oxide synthase activity,GO:0005516~calmodulin binding,GO:0010181~FMN binding,GO:0016491~oxidoreductase activity,GO:0020037~heme binding,GO:0046872~metal ion binding,GO:0050660~flavin adenine dinucleotide binding,GO:0050661~NADP binding, |
| RMARP22312 | REST corepressor 3(RCOR3) | GO:0006357~regulation of transcription by RNA polymerase II,GO:0045892~negative regulation of DNA-templated transcription, | GO:0000118~histone deacetylase complex,GO:0005634~nucleus,GO:0005654~nucleoplasm,GO:0005667~transcription regulator complex,GO:0005829~cytosol, | GO:0003714~transcription corepressor activity,GO:0005515~protein binding, |
| RMARP22675 | vacuolar protein sorting 13 homolog A(vps13a) | GO:0006869~lipid transport, | GO:0005811~lipid droplet, | N/A |
| RMARP22795 | sarcoglycan epsilon(SGCE) | GO:0007160~cell-matrix adhesion,GO:0007517~muscle organ development, | GO:0005737~cytoplasm,GO:0005794~Golgi apparatus,GO:0005856~cytoskeleton,GO:0005886~plasma membrane,GO:0016010~dystrophin-associated glycoprotein complex,GO:0016012~sarcoglycan complex,GO:0030425~dendrite,GO:0032590~dendrite membrane,GO:0042383~sarcolemma, | GO:0005509~calcium ion binding, |
| RMARP22919 | adherens junction associated protein 1(Ajap1) | GO:0001953~negative regulation of cell-matrix adhesion,GO:0007155~cell adhesion,GO:0030860~regulation of polarized epithelial cell differentiation,GO:0061045~negative regulation of wound healing, | GO:0005886~plasma membrane,GO:0005912~adherens junction,GO:0009898~cytoplasmic side of plasma membrane,GO:0009986~cell surface,GO:0016323~basolateral plasma membrane,GO:0016324~apical plasma membrane,GO:0044291~cell-cell contact zone, | GO:0008013~beta-catenin binding,GO:0019904~protein domain specific binding,GO:0044877~protein-containing complex binding, |
| RMARP22924 | fer (fps/fes related) tyrosine kinase(fer) | GO:0006935~chemotaxis,GO:0007155~cell adhesion,GO:0038095~Fc-epsilon receptor signaling pathway,GO:0043304~regulation of mast cell degranulation, | GO:0005737~cytoplasm,GO:0005856~cytoskeleton,GO:0005886~plasma membrane, | GO:0004713~protein tyrosine kinase activity,GO:0004715~non-membrane spanning protein tyrosine kinase activity,GO:0005524~ATP binding,GO:0008289~lipid binding,GO:0016301~kinase activity, |
| RMARP23179 | alpha-1,3-mannosyl-glycoprotein 4-beta-N-acetylglucosaminyltransferase A(MGAT4A) | GO:0006487~protein N-linked glycosylation,GO:0006491~N-glycan processing,GO:0046487~glyoxylate metabolic process, | GO:0000139~Golgi membrane,GO:0005576~extracellular region,GO:0005777~peroxisome,GO:0005783~endoplasmic reticulum,GO:0005793~endoplasmic reticulum-Golgi intermediate compartment,GO:0005795~Golgi stack,GO:0016020~membrane, | GO:0008375~acetylglucosaminyltransferase activity,GO:0008453~alanine-glyoxylate transaminase activity,GO:0008454~alpha-1,3-mannosylglycoprotein 4-beta-N-acetylglucosaminyltransferase activity,GO:0042803~protein homodimerization activity,GO:0046872~metal ion binding, |
| RMARP23309 | nucleoredoxin like 2(nxnl2) | GO:0007600~sensory perception,GO:0007601~visual perception,GO:0007608~sensory perception of smell,GO:0045494~photoreceptor cell maintenance, | N/A | N/A |
| RMARP23367 | adaptor related protein complex 1 associated regulatory protein(ap1ar) | GO:0034315~regulation of Arp2/3 complex-mediated actin nucleation,GO:0048203~vesicle targeting, trans-Golgi to endosome,GO:1900025~negative regulation of substrate adhesion-dependent cell spreading,GO:2000146~negative regulation of cell motility, | GO:0005768~endosome,GO:0005829~cytosol, | GO:0019894~kinesin binding,GO:0035650~AP-1 adaptor complex binding, |
| RMARP23368 | TRAF-interacting protein with forkhead-associated domain(tifa) | GO:0002753~cytoplasmic pattern recognition receptor signaling pathway,GO:0043123~positive regulation of canonical NF-kappaB signal transduction,GO:0045087~innate immune response,GO:0051260~protein homooligomerization, | GO:0005737~cytoplasm, | N/A |
| RMARP23533 | Sin3A associated protein 30kDa(sap30) | GO:0006355~regulation of DNA-templated transcription, | GO:0000118~histone deacetylase complex,GO:0005634~nucleus, | GO:0003677~DNA binding,GO:0003712~transcription coregulator activity,GO:0046872~metal ion binding, |
| RMARP23809 | sorting nexin 2 (snx2) | N/A | N/A | N/A |
| RMARP23809 | sorting nexin 1(SNX1) | GO:0006886~intracellular protein transport,GO:0031623~receptor internalization,GO:0034498~early endosome to Golgi transport,GO:0072673~lamellipodium morphogenesis, | GO:0005764~lysosome,GO:0005768~endosome,GO:0005794~Golgi apparatus,GO:0005829~cytosol,GO:0010008~endosome membrane,GO:0030027~lamellipodium,GO:0030905~retromer, tubulation complex,GO:0031901~early endosome membrane, | GO:0005154~epidermal growth factor receptor binding,GO:0005158~insulin receptor binding,GO:0035091~phosphatidylinositol binding,GO:0042803~protein homodimerization activity,GO:0046982~protein heterodimerization activity,GO:1990459~transferrin receptor binding,GO:1990460~leptin receptor binding, |
| RMARP23875 | mitogen-activated protein kinase 3(Mapk3) | GO:0000165~MAPK cascade,GO:0006351~DNA-templated transcription,GO:0006468~protein phosphorylation,GO:0006796~phosphate-containing compound metabolic process,GO:0006915~apoptotic process,GO:0006974~DNA damage response,GO:0007165~signal transduction,GO:0007166~cell surface receptor signaling pathway,GO:0007254~JNK cascade,GO:0008286~insulin receptor signaling pathway,GO:0009636~response to toxic substance,GO:0009887~animal organ morphogenesis,GO:0010759~positive regulation of macrophage chemotaxis,GO:0014032~neural crest cell development,GO:0014044~Schwann cell development,GO:0016310~phosphorylation,GO:0019233~sensory perception of pain,GO:0030278~regulation of ossification,GO:0030509~BMP signaling pathway,GO:0030641~regulation of cellular pH,GO:0030878~thyroid gland development,GO:0031281~positive regulation of cyclase activity,GO:0031663~lipopolysaccharide-mediated signaling pathway,GO:0032206~positive regulation of telomere maintenance,GO:0032212~positive regulation of telomere maintenance via telomerase,GO:0032496~response to lipopolysaccharide,GO:0032872~regulation of stress-activated MAPK cascade,GO:0034198~cellular response to amino acid starvation,GO:0034614~cellular response to reactive oxygen species,GO:0035556~intracellular signal transduction,GO:0038083~peptidyl-tyrosine autophosphorylation,GO:0038133~ERBB2-ERBB3 signaling pathway,GO:0042473~outer ear morphogenesis,GO:0042552~myelination,GO:0042770~signal transduction in response to DNA damage,GO:0043330~response to exogenous dsRNA,GO:0045727~positive regulation of translation,GO:0045944~positive regulation of transcription by RNA polymerase II,GO:0046697~decidualization,GO:0048009~insulin-like growth factor receptor signaling pathway,GO:0048538~thymus development,GO:0050804~modulation of chemical synaptic transmission,GO:0051090~regulation of DNA-binding transcription factor activity,GO:0051216~cartilage development,GO:0051403~stress-activated MAPK cascade,GO:0051493~regulation of cytoskeleton organization,GO:0060020~Bergmann glial cell differentiation,GO:0060324~face development,GO:0060425~lung morphogenesis,GO:0060440~trachea formation,GO:0061308~cardiac neural crest cell development involved in heart development,GO:0065003~protein-containing complex assembly,GO:0070371~ERK1 and ERK2 cascade,GO:0070374~positive regulation of ERK1 and ERK2 cascade,GO:0070498~interleukin-1-mediated signaling pathway,GO:0070849~response to epidermal growth factor,GO:0071260~cellular response to mechanical stimulus,GO:0071276~cellular response to cadmium ion,GO:0071356~cellular response to tumor necrosis factor,GO:0072584~caveolin-mediated endocytosis,GO:0090170~regulation of Golgi inheritance,GO:0097237~cellular response to toxic substance,GO:0098792~xenophagy,GO:0120041~positive regulation of macrophage proliferation,GO:1904262~negative regulation of TORC1 signaling,GO:1904355~positive regulation of telomere capping,GO:1904417~positive regulation of xenophagy,GO:2000641~regulation of early endosome to late endosome transport,GO:2000657~negative regulation of apolipoprotein binding, | GO:0005634~nucleus,GO:0005635~nuclear envelope,GO:0005654~nucleoplasm,GO:0005737~cytoplasm,GO:0005739~mitochondrion,GO:0005769~early endosome,GO:0005770~late endosome,GO:0005794~Golgi apparatus,GO:0005829~cytosol,GO:0005856~cytoskeleton,GO:0005886~plasma membrane,GO:0005901~caveola,GO:0005925~focal adhesion,GO:0031143~pseudopodium,GO:0032991~protein-containing complex,GO:0098978~glutamatergic synapse, | GO:0001784~phosphotyrosine residue binding,GO:0004672~protein kinase activity,GO:0004674~protein serine/threonine kinase activity,GO:0004705~JUN kinase activity,GO:0004707~MAP kinase activity,GO:0005515~protein binding,GO:0005524~ATP binding,GO:0019902~phosphatase binding,GO:0042802~identical protein binding,GO:0097110~scaffold protein binding,GO:0106310~protein serine kinase activity,GO:0140297~DNA-binding transcription factor binding, |
| RMARP23882 | mitochondrial ribosomal protein L40(MRPL40) | GO:0009653~anatomical structure morphogenesis,GO:0032543~mitochondrial translation, | GO:0005634~nucleus,GO:0005730~nucleolus,GO:0005739~mitochondrion,GO:0005743~mitochondrial inner membrane,GO:0005761~mitochondrial ribosome,GO:0005762~mitochondrial large ribosomal subunit, | GO:0003723~RNA binding,GO:0005515~protein binding, |
| RMARP23883 | chromosome 26 C22orf39 homolog(C26H22orf39) | GO:1900272~negative regulation of long-term synaptic potentiation, | GO:0043083~synaptic cleft,GO:0045202~synapse, | N/A |
| RMARP23895 | Guanine nucleotide binding protein (G protein), beta polypeptide 1-like (gnb1l) | N/A | N/A | N/A |
| RMARP23960 | complement C2(C2) | GO:0006508~proteolysis,GO:0006956~complement activation,GO:0006958~complement activation, classical pathway,GO:0007584~response to nutrient,GO:0009617~response to bacterium,GO:0032496~response to lipopolysaccharide,GO:0045087~innate immune response,GO:0097066~response to thyroid hormone,GO:2000427~positive regulation of apoptotic cell clearance, | GO:0005576~extracellular region,GO:0005601~classical-complement-pathway C3/C5 convertase complex,GO:0005615~extracellular space,GO:0070062~extracellular exosome, | GO:0004252~serine-type endopeptidase activity,GO:0005515~protein binding,GO:0046872~metal ion binding, |
| RMARP24439 | ELAV like neuron-specific RNA binding protein 2(elavl2) | N/A | GO:0005737~cytoplasm,GO:0005938~cell cortex,GO:1990904~ribonucleoprotein complex, | GO:0003723~RNA binding, |
| RMARP25279 | microsomal glutathione S-transferase 2(mgst2) | GO:0006691~leukotriene metabolic process,GO:0019370~leukotriene biosynthetic process, | GO:0005635~nuclear envelope,GO:0005783~endoplasmic reticulum,GO:0005789~endoplasmic reticulum membrane,GO:0016020~membrane, | GO:0004364~glutathione transferase activity,GO:0004464~leukotriene-C4 synthase activity,GO:0004602~glutathione peroxidase activity,GO:0008047~enzyme activator activity, |
| RMARP25280 | RAB11B, member RAS oncogene family, b(rab11bb) | GO:0045054~constitutive secretory pathway,GO:0045055~regulated exocytosis, | GO:0005794~Golgi apparatus,GO:0055037~recycling endosome, | GO:0000166~nucleotide binding,GO:0003924~GTPase activity,GO:0005525~GTP binding, |
| RMARP25288 | N(alpha)-acetyltransferase 15, NatA auxiliary subunit(naa15) | N/A | GO:0005737~cytoplasm,GO:0031415~NatA complex, | GO:0010698~acetyltransferase activator activity, |
| RMARP25402 | Rap guanine nucleotide exchange factor 2(rapgef2) | GO:0007264~small GTPase-mediated signal transduction,GO:0007399~nervous system development,GO:0030154~cell differentiation, | GO:0005737~cytoplasm,GO:0005768~endosome,GO:0005770~late endosome,GO:0005886~plasma membrane,GO:0048471~perinuclear region of cytoplasm,GO:0070161~anchoring junction, | GO:0005085~guanyl-nucleotide exchange factor activity,GO:0005096~GTPase activator activity, |
| RMARP25404 | sperm mitochondria-associated cysteine-rich protein(Smcp) | GO:0007339~binding of sperm to zona pellucida,GO:0007341~penetration of zona pellucida,GO:0030317~flagellated sperm motility, | GO:0005737~cytoplasm,GO:0005739~mitochondrion,GO:0005741~mitochondrial outer membrane, | N/A |
| RMARP25518 | MAM domain containing 2(mamdc2) | N/A | GO:0016020~membrane, | N/A |
| RMARP25647 | CCR4-NOT transcription complex subunit 2(CNOT2) | GO:0000122~negative regulation of transcription by RNA polymerase II,GO:0000289~nuclear-transcribed mRNA poly(A) tail shortening,GO:0001829~trophectodermal cell differentiation,GO:0006355~regulation of DNA-templated transcription,GO:0006357~regulation of transcription by RNA polymerase II,GO:0006417~regulation of translation,GO:0010606~positive regulation of cytoplasmic mRNA processing body assembly,GO:0031047~regulatory ncRNA-mediated gene silencing,GO:0033147~negative regulation of intracellular estrogen receptor signaling pathway,GO:2000036~regulation of stem cell population maintenance, | GO:0000932~P-body,GO:0005634~nucleus,GO:0005654~nucleoplasm,GO:0005737~cytoplasm,GO:0005829~cytosol,GO:0005886~plasma membrane,GO:0016020~membrane,GO:0030014~CCR4-NOT complex,GO:0030015~CCR4-NOT core complex, | GO:0001222~transcription corepressor binding,GO:0003712~transcription coregulator activity,GO:0004535~poly(A)-specific ribonuclease activity,GO:0005515~protein binding, |
| RMARP25655 | Dmx-like 1(dmxl1) | GO:0007035~vacuolar acidification, | GO:0043291~RAVE complex, | N/A |
| RMARP25844 | sorbin and SH3 domain containing 2(SORBS2) | GO:0007219~Notch signaling pathway, | GO:0005886~plasma membrane,GO:0030425~dendrite,GO:0043025~neuronal cell body,GO:0045202~synapse, | GO:0005515~protein binding, |
| RMARP25863 | acyl-CoA synthetase long chain family member 6(acsl6) | GO:0000038~very long-chain fatty acid metabolic process,GO:0001676~long-chain fatty acid metabolic process,GO:0006631~fatty acid metabolic process,GO:0035338~long-chain fatty-acyl-CoA biosynthetic process, | GO:0005783~endoplasmic reticulum,GO:0016020~membrane, | GO:0004467~long-chain fatty acid-CoA ligase activity,GO:0005524~ATP binding,GO:0016874~ligase activity,GO:0047676~arachidonate-CoA ligase activity, |
| RMARP25876 | kinase insert domain receptor(kdr) | GO:0001525~angiogenesis,GO:0001568~blood vessel development,GO:0007169~cell surface receptor protein tyrosine kinase signaling pathway,GO:0007275~multicellular organism development,GO:0016477~cell migration,GO:0030154~cell differentiation,GO:0030335~positive regulation of cell migration,GO:0043408~regulation of MAPK cascade,GO:0045446~endothelial cell differentiation,GO:0045766~positive regulation of angiogenesis,GO:0048010~vascular endothelial growth factor receptor signaling pathway, | GO:0005768~endosome,GO:0005769~early endosome,GO:0005783~endoplasmic reticulum,GO:0005886~plasma membrane,GO:0016020~membrane,GO:0043235~receptor complex,GO:0070161~anchoring junction, | GO:0004713~protein tyrosine kinase activity,GO:0005004~GPI-linked ephrin receptor activity,GO:0005005~transmembrane-ephrin receptor activity,GO:0005006~epidermal growth factor receptor activity,GO:0005007~fibroblast growth factor receptor activity,GO:0005008~hepatocyte growth factor receptor activity,GO:0005009~insulin receptor activity,GO:0005010~insulin-like growth factor receptor activity,GO:0005011~macrophage colony-stimulating factor receptor activity,GO:0005018~platelet-derived growth factor alpha-receptor activity,GO:0005019~platelet-derived growth factor beta-receptor activity,GO:0005020~stem cell factor receptor activity,GO:0005021~vascular endothelial growth factor receptor activity,GO:0005524~ATP binding,GO:0008288~boss receptor activity,GO:0019838~growth factor binding,GO:0036332~placental growth factor receptor activity,GO:0038062~protein tyrosine kinase collagen receptor activity,GO:0046872~metal ion binding,GO:0060175~brain-derived neurotrophic factor receptor activity, |
| RMARP25877 | Novel gene | N/A | N/A | N/A |
| RMARP25889 | WW and C2 domain containing 2(wwc2) | GO:0008285~negative regulation of cell population proliferation, | GO:0005737~cytoplasm,GO:0005829~cytosol, | N/A |
| RMARP25931 | KN motif and ankyrin repeat domains 4(KANK4) | GO:0030837~negative regulation of actin filament polymerization, | GO:0005737~cytoplasm,GO:0005829~cytosol,GO:0005856~cytoskeleton,GO:0015630~microtubule cytoskeleton, | GO:0005515~protein binding, |
| RMARP25989 | high mobility group 20B(hmg20b) | GO:0010468~regulation of gene expression, | GO:0005634~nucleus, | GO:0003677~DNA binding, |
| RMARP27133 | Von Willebrand factor A domain containing 5A (vwa5a) | N/A | N/A | N/A |
| RMARP27743 | Novel gene | N/A | N/A | N/A |
| RMARP27744 | Novel gene | N/A | N/A | N/A |
| RMARP28256 | teneurin transmembrane protein 3(tenm3) | GO:0007165~signal transduction, | GO:0005886~plasma membrane,GO:0016020~membrane,GO:0042995~cell projection, | GO:0005509~calcium ion binding, |
| RMARP28281 | abhydrolase domain containing 18(ABHD18) | N/A | GO:0005576~extracellular region,GO:0005739~mitochondrion, | N/A |
| RMARP28282 | Novel gene | N/A | N/A | N/A |
| RMARP28283 | La ribonucleoprotein domain family member 1B(larp1b) | GO:0045727~positive regulation of translation,GO:0048255~mRNA stabilization, | GO:0005829~cytosol,GO:0010494~cytoplasmic stress granule, | GO:0000339~RNA cap binding,GO:0003723~RNA binding, |
| RMARP28288 | jade family PHD finger 1(jade1) | GO:0006357~regulation of transcription by RNA polymerase II,GO:0006915~apoptotic process, | GO:0000123~histone acetyltransferase complex,GO:0005634~nucleus,GO:0005694~chromosome,GO:0005856~cytoskeleton,GO:0042995~cell projection, | GO:0046872~metal ion binding, |
| RMARP28290 | sodium channel and clathrin linker 1(sclt1) | GO:0034220~monoatomic ion transmembrane transport,GO:0045162~clustering of voltage-gated sodium channels,GO:0060271~cilium assembly, | GO:0005814~centriole, | N/A |
| RMARP28293 | cyclin dependent kinase like 3(CDKL3) | GO:0000278~mitotic cell cycle,GO:0030517~negative regulation of axon extension,GO:0036211~protein modification process,GO:0050775~positive regulation of dendrite morphogenesis,GO:0051726~regulation of cell cycle,GO:0097484~dendrite extension, | GO:0005634~nucleus,GO:0005737~cytoplasm,GO:0005829~cytosol, | GO:0004672~protein kinase activity,GO:0004674~protein serine/threonine kinase activity,GO:0004693~cyclin-dependent protein serine/threonine kinase activity,GO:0005515~protein binding,GO:0005524~ATP binding,GO:0016301~kinase activity,GO:0017018~myosin phosphatase activity,GO:0106310~protein serine kinase activity, |
| RMARP28332 | CDC28 protein kinase regulatory subunit 2(cks2) | GO:0051301~cell division, | N/A | GO:0016301~kinase activity,GO:0016538~cyclin-dependent protein serine/threonine kinase regulator activity,GO:0035368~selenocysteine insertion sequence binding, |
| RMARP29550 | Uncharacterised gene | N/A | N/A | N/A |
| RMARP29729 | RNA binding protein gene with multiple splicing(Rbpms) | GO:0000381~regulation of alternative mRNA splicing, via spliceosome,GO:0006979~response to oxidative stress,GO:0045893~positive regulation of DNA-templated transcription,GO:0060395~SMAD protein signal transduction,GO:0065003~protein-containing complex assembly, | GO:0000932~P-body,GO:0005634~nucleus,GO:0005654~nucleoplasm,GO:0005737~cytoplasm,GO:0005829~cytosol,GO:0010494~cytoplasmic stress granule,GO:0016020~membrane, | GO:0003713~transcription coactivator activity,GO:0003723~RNA binding,GO:0003729~mRNA binding,GO:0003730~mRNA 3'-UTR binding,GO:0036002~pre-mRNA binding,GO:0042802~identical protein binding,GO:0042803~protein homodimerization activity,GO:0060090~molecular adaptor activity,GO:0097157~pre-mRNA intronic binding,GO:1990715~mRNA CDS binding, |
| RMARP29941 | Reverse transcriptase domain-containing protein (A0A674PH45_TAKRU) | N/A | N/A | GO:0003824~catalytic activity, |
| RMARP30391 | platelet derived growth factor receptor like(pdgfrl) | N/A | GO:0005576~extracellular region, | GO:0042802~identical protein binding, |
| RMARP30759 | Protein phosphatase 4 catalytic subunit (ppp4c) | N/A | N/A | N/A |
| RMARP30912 | multiple PDZ domain protein(mpdz) | GO:0120192~tight junction assembly, | GO:0005737~cytoplasm,GO:0005886~plasma membrane,GO:0005923~bicellular tight junction,GO:0016324~apical plasma membrane,GO:0045177~apical part of cell, | N/A |
| RMARP31755 | Novel gene | N/A | N/A | N/A |
| RMARP31838 | LSM4 homolog, U6 small nuclear RNA and mRNA degradation associated(LSM4) | GO:0000387~spliceosomal snRNP assembly,GO:0000398~mRNA splicing, via spliceosome,GO:0000956~nuclear-transcribed mRNA catabolic process,GO:0006396~RNA processing,GO:0033962~P-body assembly, | GO:0000932~P-body,GO:0005681~spliceosomal complex,GO:0005688~U6 snRNP,GO:0005829~cytosol,GO:0016020~membrane,GO:0046540~U4/U6 x U5 tri-snRNP complex,GO:0071005~U2-type precatalytic spliceosome,GO:0097526~spliceosomal tri-snRNP complex,GO:0120115~Lsm2-8 complex, | GO:0003723~RNA binding,GO:0017070~U6 snRNA binding,GO:0042731~PH domain binding, |
| RMARP32318 | phosphoglucomutase 5(pgm5) | GO:0005975~carbohydrate metabolic process,GO:0014706~striated muscle tissue development,GO:0030239~myofibril assembly, | GO:0005829~cytosol,GO:0005912~adherens junction,GO:0030055~cell-substrate junction,GO:0042383~sarcolemma, | GO:0000287~magnesium ion binding,GO:0004614~phosphoglucomutase activity,GO:0046872~metal ion binding, |
| RMARP32325 | tight junction protein 2(TJP2) | GO:0034109~homotypic cell-cell adhesion,GO:0035633~maintenance of blood-brain barrier,GO:0045216~cell-cell junction organization,GO:0046037~GMP metabolic process,GO:0046710~GDP metabolic process,GO:0050892~intestinal absorption,GO:0090557~establishment of endothelial intestinal barrier,GO:0090559~regulation of membrane permeability,GO:0098609~cell-cell adhesion,GO:0150105~protein localization to cell-cell junction,GO:1905605~positive regulation of blood-brain barrier permeability, | GO:0005634~nucleus,GO:0005654~nucleoplasm,GO:0005829~cytosol,GO:0005886~plasma membrane,GO:0005912~adherens junction,GO:0005923~bicellular tight junction,GO:0044291~cell-cell contact zone, | GO:0004385~guanylate kinase activity,GO:0005515~protein binding,GO:0019904~protein domain specific binding,GO:0030674~protein-macromolecule adaptor activity,GO:0044325~transmembrane transporter binding,GO:0045296~cadherin binding,GO:0050839~cell adhesion molecule binding,GO:1990782~protein tyrosine kinase binding, |
| RMARP33200 | protein phosphatase 3, catalytic subunit, alpha isozyme(ppp3ca) | GO:0033173~calcineurin-NFAT signaling cascade,GO:0097720~calcineurin-mediated signaling, | GO:0005634~nucleus,GO:0005737~cytoplasm,GO:0005829~cytosol,GO:0005955~calcineurin complex,GO:0016020~membrane, | GO:0004721~phosphoprotein phosphatase activity,GO:0004723~calcium-dependent protein serine/threonine phosphatase activity,GO:0005516~calmodulin binding,GO:0017018~myosin phosphatase activity,GO:0033192~calmodulin-dependent protein phosphatase activity,GO:0046872~metal ion binding,GO:0140791~histone H2AXS140 phosphatase activity,GO:0180004~RNA polymerase II CTD heptapeptide repeat Y1 phosphatase activity,GO:0180005~RNA polymerase II CTD heptapeptide repeat T4 phosphatase activity,GO:0180006~RNA polymerase II CTD heptapeptide repeat S2 phosphatase activity,GO:0180007~RNA polymerase II CTD heptapeptide repeat S5 phosphatase activity,GO:0180008~RNA polymerase II CTD heptapeptide repeat S7 phosphatase activity,GO:1990439~MAP kinase serine/threonine phosphatase activity, |
| RMARP33287 | N-acylsphingosine amidohydrolase 1(ASAH1) | GO:0006631~fatty acid metabolic process,GO:0006665~sphingolipid metabolic process,GO:0030216~keratinocyte differentiation,GO:0045892~negative regulation of DNA-templated transcription,GO:0046512~sphingosine biosynthetic process,GO:0046513~ceramide biosynthetic process,GO:0046514~ceramide catabolic process,GO:0050810~regulation of steroid biosynthetic process,GO:0062098~regulation of programmed necrotic cell death,GO:0071356~cellular response to tumor necrosis factor, | GO:0005576~extracellular region,GO:0005615~extracellular space,GO:0005634~nucleus,GO:0005764~lysosome,GO:0016020~membrane,GO:0043202~lysosomal lumen,GO:0070062~extracellular exosome,GO:1904724~tertiary granule lumen,GO:1904813~ficolin-1-rich granule lumen, | GO:0003714~transcription corepressor activity,GO:0005515~protein binding,GO:0016787~hydrolase activity,GO:0016811~hydrolase activity, acting on carbon-nitrogen (but not peptide) bonds, in linear amides,GO:0016922~nuclear receptor binding,GO:0017040~N-acylsphingosine amidohydrolase activity,GO:0017064~fatty acid amide hydrolase activity, |
| RMARP34326 | tankyrase, TRF1-interacting ankyrin-related ADP-ribose polymerase(tnks) | GO:1904108~protein localization to ciliary inversin compartment, | GO:0097543~ciliary inversin compartment, | GO:0003950~NAD+-protein poly-ADP-ribosyltransferase activity,GO:0016757~glycosyltransferase activity, |
| RMARP34327 | metallothionein 1X(MT1X) | GO:0006882~intracellular zinc ion homeostasis,GO:0010038~response to metal ion,GO:0010273~detoxification of copper ion,GO:0036018~cellular response to erythropoietin,GO:0045926~negative regulation of growth,GO:0071276~cellular response to cadmium ion,GO:0071280~cellular response to copper ion,GO:0071294~cellular response to zinc ion, | GO:0005634~nucleus,GO:0005737~cytoplasm, | GO:0005515~protein binding,GO:0008270~zinc ion binding,GO:0046872~metal ion binding, |
| RMARP34709 | FXYD domain-containing ion transport regulator 6-like(LOC101063646) | GO:0006811~monoatomic ion transport,GO:0043269~regulation of monoatomic ion transport,GO:2000649~regulation of sodium ion transmembrane transporter activity, | GO:0016020~membrane, | GO:0017080~sodium channel regulator activity, |
| RMARP35111 | B cell scaffold protein with ankyrin repeats 1 (bank1) | N/A | N/A | N/A |
| RMARP35976 | RasGEF domain family member 1B(RASGEF1B) | GO:0007264~small GTPase-mediated signal transduction,GO:0007265~Ras protein signal transduction, | GO:0005769~early endosome,GO:0005770~late endosome,GO:0005886~plasma membrane,GO:0030496~midbody, | GO:0005085~guanyl-nucleotide exchange factor activity,GO:0005515~protein binding, |
| RMARP36017 | neuropeptide FF receptor 1(NPFFR1) | GO:0007186~G protein-coupled receptor signaling pathway,GO:0007218~neuropeptide signaling pathway,GO:0032870~cellular response to hormone stimulus, | GO:0005886~plasma membrane,GO:0005929~cilium, | GO:0004930~G protein-coupled receptor activity,GO:0005515~protein binding,GO:0008188~neuropeptide receptor activity,GO:0042277~peptide binding, |
| RMARP36282 | transmembrane protein 241(TMEM241) | GO:0055085~transmembrane transport,GO:1990569~UDP-N-acetylglucosamine transmembrane transport,GO:1990570~GDP-mannose transmembrane transport, | GO:0005794~Golgi apparatus,GO:0016020~membrane, | GO:0005458~GDP-mannose transmembrane transporter activity,GO:0005462~UDP-N-acetylglucosamine transmembrane transporter activity,GO:0005515~protein binding,GO:0015297~antiporter activity, |
| RMARP36418 | doublecortin like kinase 1(DCLK1) | GO:0001764~neuron migration,GO:0006338~chromatin remodeling,GO:0006468~protein phosphorylation,GO:0007399~nervous system development,GO:0007417~central nervous system development,GO:0009615~response to virus,GO:0016197~endosomal transport,GO:0021952~central nervous system projection neuron axonogenesis,GO:0030900~forebrain development,GO:0034504~protein localization to nucleus,GO:0035082~axoneme assembly,GO:0035556~intracellular signal transduction,GO:0048675~axon extension,GO:0048812~neuron projection morphogenesis,GO:0048813~dendrite morphogenesis,GO:0060041~retina development in camera-type eye,GO:0150052~regulation of postsynapse assembly,GO:1900181~negative regulation of protein localization to nucleus, | GO:0005737~cytoplasm,GO:0005886~plasma membrane,GO:0014069~postsynaptic density,GO:0043005~neuron projection,GO:0098978~glutamatergic synapse, | GO:0004672~protein kinase activity,GO:0004674~protein serine/threonine kinase activity,GO:0004676~3-phosphoinositide-dependent protein kinase activity,GO:0004677~DNA-dependent protein kinase activity,GO:0004679~AMP-activated protein kinase activity,GO:0004694~eukaryotic translation initiation factor 2alpha kinase activity,GO:0004711~ribosomal protein S6 kinase activity,GO:0005515~protein binding,GO:0005524~ATP binding,GO:0008017~microtubule binding,GO:0035175~histone H3S10 kinase activity,GO:0035402~histone H3T11 kinase activity,GO:0035403~histone H3T6 kinase activity,GO:0035979~histone H2AXS139 kinase activity,GO:0044022~histone H3S28 kinase activity,GO:0044023~histone H4S1 kinase activity,GO:0044024~histone H2AS1 kinase activity,GO:0044025~histone H2BS14 kinase activity,GO:0072354~histone H3T3 kinase activity,GO:0072371~histone H2AS121 kinase activity,GO:0072518~Rho-dependent protein serine/threonine kinase activity,GO:0106310~protein serine kinase activity,GO:0140823~histone H2BS36 kinase activity,GO:0140855~histone H3S57 kinase activity,GO:0140857~histone H3T45 kinase activity,GO:1990244~histone H2AT120 kinase activity, |
| RMARP37056 | transmembrane serine protease 9(tmprss9) | GO:0006508~proteolysis, | GO:0016020~membrane, | GO:0004252~serine-type endopeptidase activity,GO:0008236~serine-type peptidase activity, |

## References

DeVore, J. L., Shine, R., & Ducatez, S. (2021). Spatial ecology of cane toads (Rhinella marina) in their native range: a radiotelemetric study from French Guiana. *Sci Rep*, *11*(1), 11817. <https://doi.org/10.1038/s41598-021-91262-8>

Easteal, S. (1981). The history of introductions of Bufo marinus (Amphibia: Anura); a natural experiment in evolution. *Biological Journal of the Linnean Society*, *16*(2), 93-113. <https://doi.org/10.1111/j.1095-8312.1981.tb01645.x>

Mittan‐Moreau, C. S., Kelehear, C., Toledo, L. F., Bacon, J., Guayasamin, J. M., Snyder, A., & Zamudio, K. R. (2022). Cryptic lineages and standing genetic variation across independent cane toad introductions. *Molecular Ecology*, *31*(24), 6440-6456. <https://doi.org/10.1111/mec.16713>

Slade, R. W., & Moritz, C. (1998). Phylogeography of Bufo marinus from its natural and introduced ranges. *Proc Biol Sci*, *265*(1398), 769-777. <https://doi.org/10.1098/rspb.1998.0359>

Turvey, N. (2013). *Cane toads: a tale of sugar, politics and flawed science*. Sydney University Press.

Zug, G. R., & Zug, P. B. (1979). The marine toad, Bufo marinus : a natural history resumé of native populations. <https://doi.org/https://doi.org/10.5479/si.00810282.284>
